# Fluorescent Activity-Based Probe To Image and Inhibit Factor XIa Activity in Human Plasma

**DOI:** 10.1021/acs.jmedchem.2c00845

**Published:** 2023-03-10

**Authors:** Sylwia Modrzycka, Sonia Kołt, Ty E. Adams, Stanisław Potoczek, James A. Huntington, Paulina Kasperkiewicz, Marcin Drąg

**Affiliations:** † Department of Chemical Biology and Bioimaging, Faculty of Chemistry, Wrocław University of Science and Technology, Wybrzeże Wyspiańskiego 27, 50-370 Wrocław, Poland; ‡ Department of Haematology, Cambridge Institute for Medical Research, University of Cambridge, The Keith Peters Building, Hills Road, Cambridge CB2 0XY, U.K.; § Department of Haematology, Blood Neoplasms, and Bone Marrow Transplantation, Wrocław Medical University, Pasteura 1, 50-367 Wrocław, Poland

## Abstract

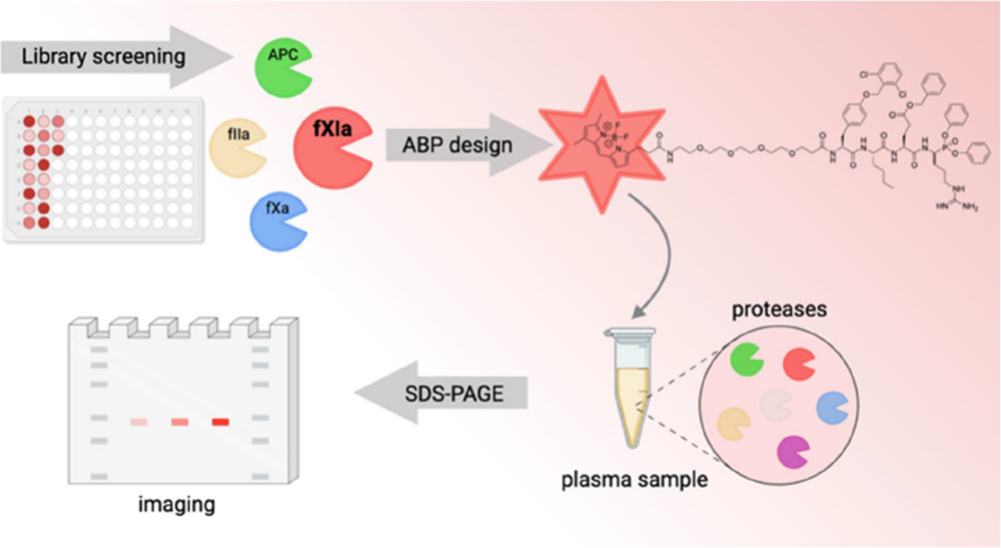

Anticoagulation therapy is a mainstay of the treatment of thrombotic disorders; however, conventional anticoagulants trade antithrombotic benefits for bleeding risk. Factor (f) XI deficiency, known as hemophilia C, rarely causes spontaneous bleeding, suggesting that fXI plays a limited role in hemostasis. In contrast, individuals with congenital fXI deficiency display a reduced incidence of ischemic stroke and venous thromboembolism, indicating that fXI plays a role in thrombosis. For these reasons, there is intense interest in pursuing fXI/factor XIa (fXIa) as targets for achieving antithrombotic benefit with reduced bleeding risk. To obtain selective inhibitors of fXIa, we employed libraries of natural and unnatural amino acids to profile fXIa substrate preferences. We developed chemical tools for investigating fXIa activity, such as substrates, inhibitors, and activity-based probes (ABPs). Finally, we demonstrated that our ABP selectively labels fXIa in the human plasma, making this tool suitable for further studies on the role of fXIa in biological samples.

## Introduction

**Figure 1:**
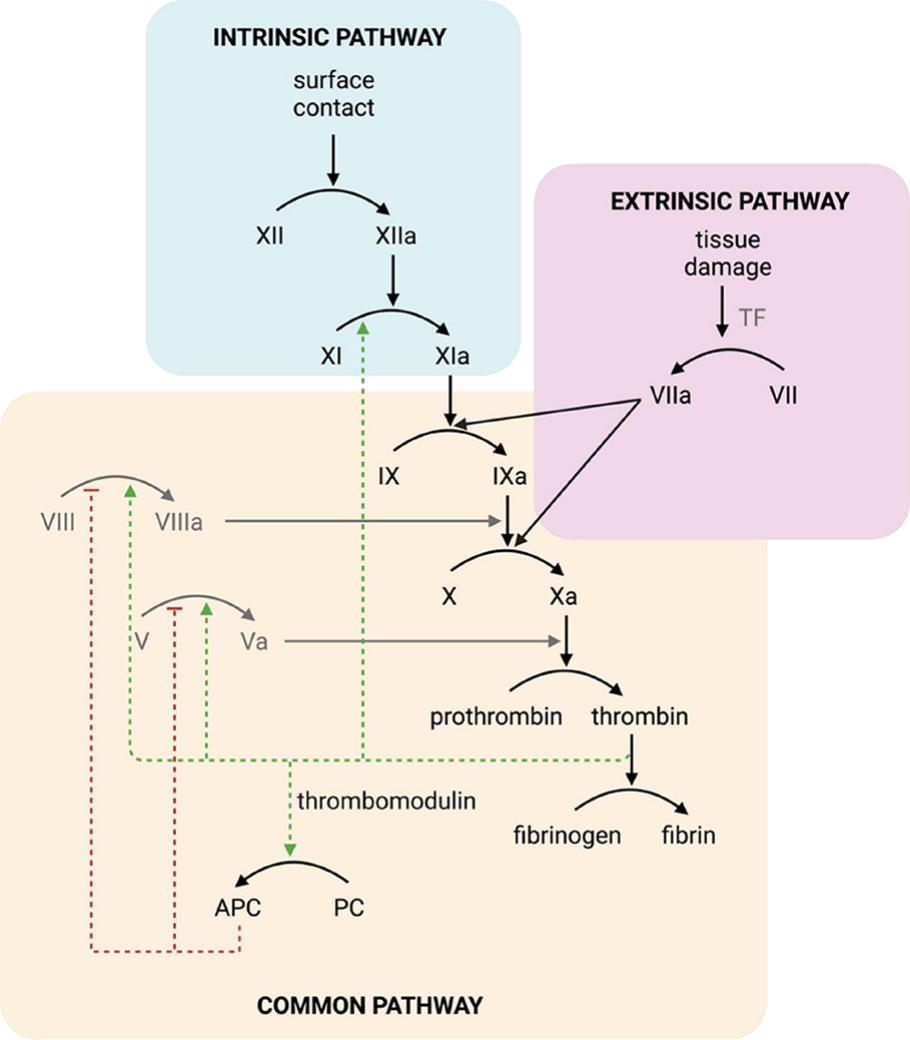
Simplified scheme showing the blood coagulation system. The cascade can be induced by two stimuli—exposure of blood to tissue factor (TF) in the subendothelial space (extrinsic pathway) or through contact with certain surfaces (intrinsic pathway)—before merging into the common pathway. Protein cofactors are indicated in gray. Red arrows indicate inhibition, and green arrows indicate activation in the positive feedback loop.

Hemostasis (blood coagulation) is a highly regulated process triggered by damage to the endothelial cell lining of the vasculature or by foreign or other negatively charged particles, resulting in a cascade of proteolytic activation events (Figure 1).^1,2^ This “coagulation cascade” involves several reactions in which zymogens of serine proteases are sequentially activated, ultimately resulting in the formation of thrombin, which deposits fibrin and platelets to form a blood clot.^2^ Normal hemostasis (extrinsic pathway) is triggered by the exposure of blood proteins to the subendothelial space, which is decorated by tissue factor (TF), which binds to fVIIa, leading to the activation of fX and fIX.^2,3^ Alternatively, the exposure of plasma proteins to negatively charged particles (e.g., kaolin and polyphosphates) supports the autoactivation of fXII, resulting in the conversion of fXI to factor XIa (fXIa), which in turn activates fIX. Both pathways converge on the formation of the prothrombinase complex, composed of factor Xa (fXa) and fVa, leading to thrombin generation and clot formation.^2,4,5^ Thrombin participates in several positive feedback loops to stimulate its own formation, including the direct activation of fXI.^6,7^ Thrombin also participates in a negative feedback loop when bound to endothelial cell surfaces via thrombomodulin, promoting the activation of protein C.^8^ Activated protein C (APC) proteolytically inactivates fVa and fVIIIa, attenuating thrombin and fXa generation.^9^ All procoagulant and anticoagulant factors operate together to maintain a balance between clotting and blood circulation. Perturbations affecting this balance may lead to severe disorders such as hemophilia^10^ and thrombosis.^11^ The ability to monitor individual coagulation factor activities, such as fXIa, in complex biological environments would improve our understanding of their functions in both physiological and pathophysiological conditions.

Congenital fXI deficiency, known as hemophilia C, is a rare condition in the general population (1 per million) but is relatively common among Ashkenazi Jews with a frequency up to 8%.^12,13^ Compared to fVIII deficiency (hemophilia A) or fIX deficiency (hemophilia B), people with hemophilia C rarely exhibit spontaneous bleeding but instead bleed excessively after injury or surgery, implying that fXI plays a limited role in normal hemostasis.^14,15^ However, elevated fXI activity is associated with an increased risk of ischemic stroke,^6,16^ deep venous thrombosis (DVT),^17,18^ and myocardial infarction (MI).^19^ These data suggest that fXI plays an important role in thromboembolic diseases, which are leading causes of mortality, responsible for one in four deaths worldwide.^20^ All currently available anticoagulants act by inhibiting one or more components of the extrinsic or common pathways. Since these pathways are crucial for the initiation of clot formation and generating the thrombin burst required for hemostasis, drugs targeting them significantly increase bleeding risk.^21,22^ FXIa is an interesting target from the contact (intrinsic) pathway that appears to contribute to the development of thrombosis but plays a minor role in hemostasis. Recent clinical studies have demonstrated that fXIa may be a potential alternative therapeutic target for achieving antithrombotic benefit with lower bleeding risk than other treatments.^22,23^

Despite the intense interest in the role of fXI in hemostasis and thrombosis, its physiological and pathophysiological functions are poorly understood compared with those of other coagulation proteases. This is partly due to its structural complexity. FXI circulates in the blood as a homodimer composed of two 80 kDa subunits, which is a unique configuration among coagulation proteases. Each subunit contains four repeats called apple domains and a trypsin-like catalytic domain.^10,24^ FXI is converted to fXIa by cleavage of the Arg369-Ile370 bond, either by fXIIa in the early stage of the intrinsic pathway or by thrombin in the amplification loop.^25−27^ The sole function of fXIa is thought to be activation of fIX through cleavage at two sites in its activation peptide: 142Lys-Leu-Thr-Arg-Ala-Glu-Thr-Val149 and 177Asp-Phe-Thr-Arg-Val-Val-Gly-Gly184 (P4-P4′).^28,29^ So far, the substrate specify profile for fXIa has been determined based on the recognition motif in physiological substrates^30,31^ and the crystal structure of the fXIa catalytic domain.^7^ However, the previously obtained substrate preferences were based only on natural amino acids, which limits the development of selective chemical tools able to distinguish fXIa from other proteases involved in the coagulation cascade.

In this work, we used the hybrid combinatorial substrate library (HyCoSuL) approach^32^ to identify sequences with high activity and selectivity for fXIa. This library contains a combination of natural and unnatural amino acids, which allows more extensive exploration of the chemical space around the protease active site. We compared the fXIa substrate specificity profile with previously obtained data for APC, thrombin, and fXa.^33^ Based on these findings, we designed and synthesized substrates and assessed the catalytic efficiency of fXIa cleavage and specificity relative to other coagulation factors. The most selective substrate was then converted into an inhibitor and a novel activity-based probe (ABP) with BODIPY FL fluorophore. Finally, we demonstrated that our fluorescent ABP selectively labeled fXIa from a mixture of purified coagulation factors and in the complex environment of human plasma, supporting its use in studying fXIa activity in normal hemostasis and thrombosis.

## Results

### Substrate Specificity of fXIa at the P1 Position

**Figure 2:**
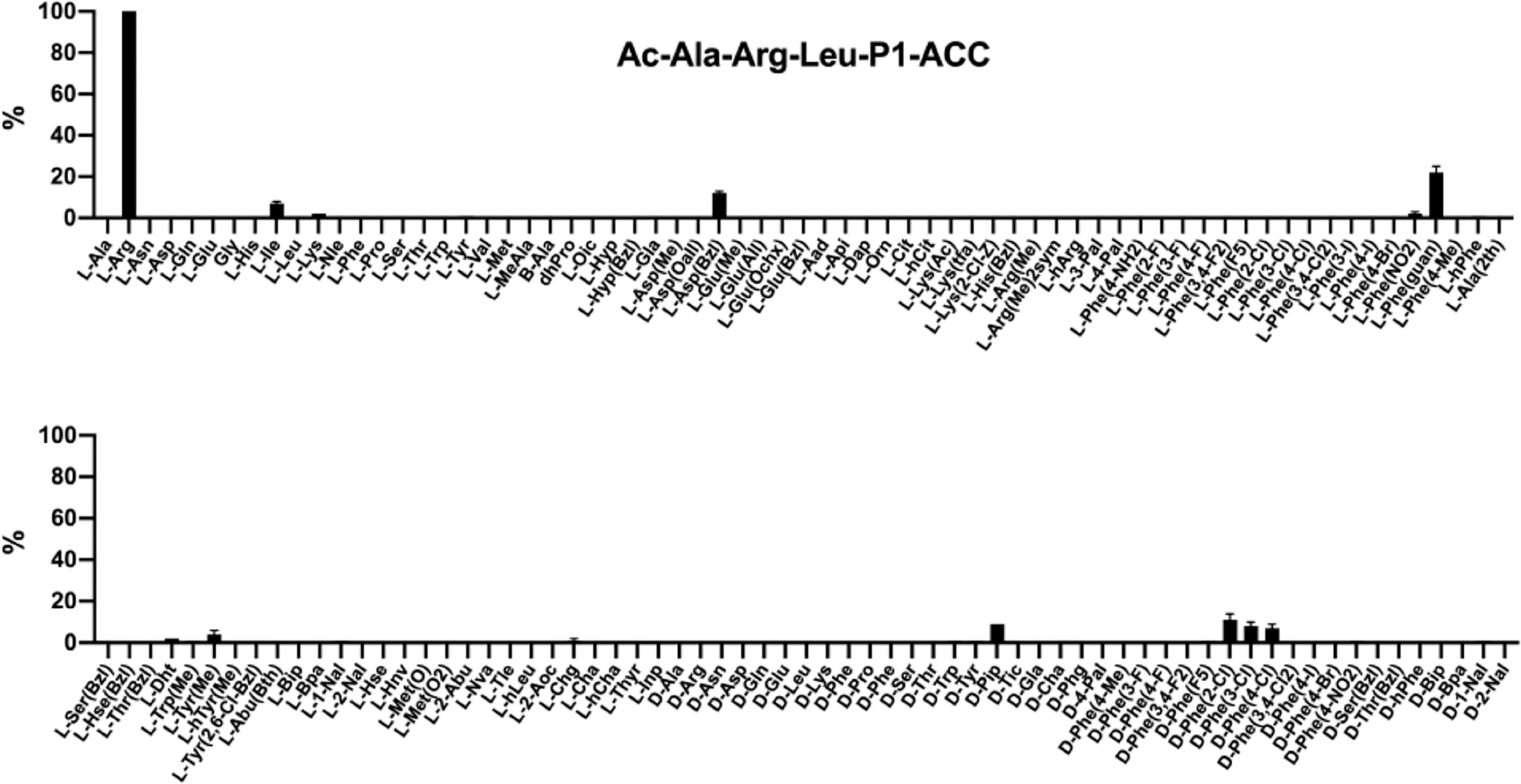
Substrate specificity profile of fXIa at the P1 position. The preference was determined using a defined library treated with fXIa. The substrate hydrolysis rate was measured as an increase in fluorescence over time (RFU/s) for 30 min (λ_ex_ = 355 nm, λ_em_ = 460 nm). The substrate specificity profile was established by setting the highest RFU/s value to 100% and adjusting other results accordingly. The average relative activity is presented as a percentage of that of the best-recognized amino acid (*n* = 2, where *n* represents the number of independent experiments).

The specificity of fXIa at the P1 position was probed using the same library as in our previous studies on APC, thrombin, and fXa^33^—a tailored library of fluorogenic substrates with a constant P4-P2 motif and various amino acid residues at the P1 position. The general structure of the library was Ac-Ala-Arg-Leu-P1-ACC (P1 was an individual natural or unnatural amino acid, ACC was a fluorescent tag (7-amino-4-carbamoylmethylcoumarin), and Ac was an acetyl group) (Figures 2 and S1).^34^ Using this library, we demonstrated that basic l-Arg (100%) was the best recognized natural amino acid at this position. This is in agreement with a crystal structure of the fXIa catalytic domain, in which the Asp189 residue serves as the recognition site to accommodate a guanidine group of arginine.^7^ Additionally, the preference for arginine at the P1 position was also previously reported in two physiologically relevant fXIa substrates, the cleavage sequences in fIX (Lys-Leu-Thr-Arg, Glu-Phe-Ser-Arg).^30,31^ We also found that the phenylalanine derivative with a guanidine group in the *para* position l-Phe(guan) (22%) was also recognized, but its activity was five times lower than that of l-Arg. The fXIa S1 subsite tolerated several other amino acid residues (mostly phenylalanine derivatives). Based on that observation, we speculate that the fXIa S1 pocket has dual properties. It is able to bind basic amino acid side chains; however, it can also accommodate large, hydrophobic derivatives. The analysis of the P1 position demonstrated that in addition to l-amino acids, fXIa also recognizes several d-enantiomers. To increase our chances of finding selective sequences, we compared the fXIa substrate specificity at the P1 positions with the profiles of other serine proteases present in the coagulation cascade (APC, thrombin, and fXa), which we previously characterized using the same approach.^33^ Interestingly, all four proteases exhibited similar preferences at the P1 position; however, APC, thrombin, and fXa interacted exclusively with positively charged l-Arg.

### Substrate Specificity of fXIa at the P4-P2 Positions

**Figure 3:**
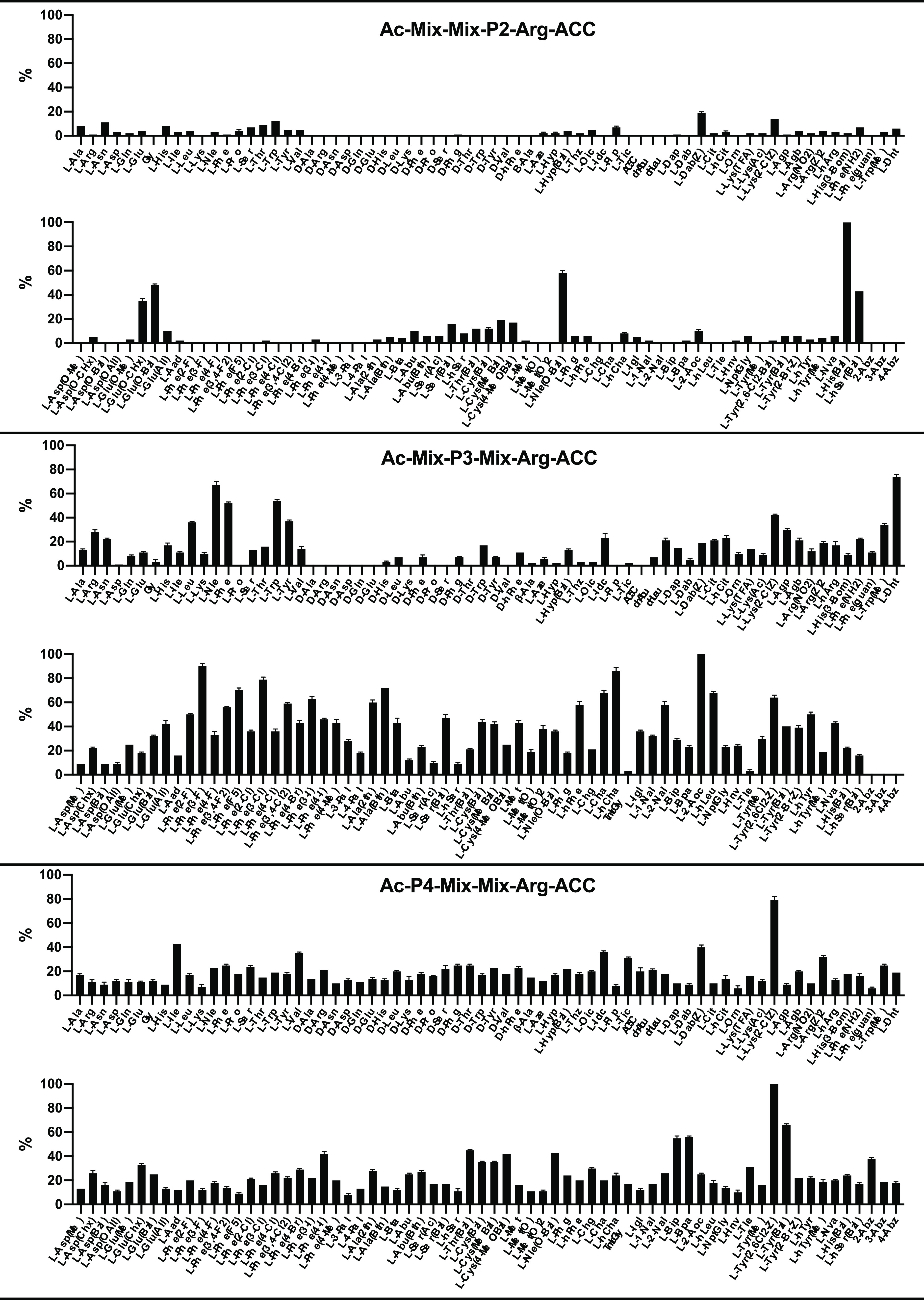
Substrate specificity profile of fXIa at the P4-P2 positions. The HyCoSuL library was treated with fXIa, and the substrate hydrolysis rate was measured as an increase in fluorescence over time (RFU/s) for 30 min (λ_ex_ = 355 nm, λ_em_ = 460 nm). The substrate specificity profile was established by setting the highest RFU/s value to 100% and adjusting other results accordingly. The average relative activity is presented as a percentage of that of the best-recognized amino acid (*n* = 2, where *n* represents the number of independent experiments).

To obtain better insight into the architecture of the fXIa S4-S2 pockets, in the next step of our study, we used the HyCoSuL approach developed by our group.^32^ Since fXIa preferentially accommodates l-Arg in the S1 pocket, we employed the previously described P1-Arg combinatorial library.^34−36^ This library consists of three tetrapeptide sublibraries (Ac-P4-Mix-Mix-Arg-ACC, Ac-Mix-P3-Mix-Arg-ACC, and Ac-Mix-Mix-P2-Arg-ACC). HyCoSuL library contains a natural and a large pool of unnatural amino acid residues at the investigated position (P4, P3, or P2) and an equimolar mixture of natural amino acids at remaining positions (Mix). The screening data allowed us to obtain a highly detailed picture of the active site preferences of fXIa (Figures 3 and S2).

We found that the fXIa S2 pocket exhibited extremely narrow substrate specificity. The relative activity of all natural amino acids was under 15%. d-Amino acids were completely ignored by fXIa, indicating that the S2 pocket is stereochemically specific. Aminobenzoic acid residues (2-Abz, 3-Abz, 4-Abz) and all phenylalanine derivatives also did not seem to be tolerated at this position. Interestingly, the only amino acids that were well accommodated in this subsite were those with a bulky benzyl group in their structure, namely, l-glutamic-acid-gamma-benzyl ester (l-Glu(Bzl), 48%), 6-benzyloxy-l-norleucine (l-Nle(O-Bzl), 58%), benzyl-l-histidine (l-His(Bzl), 100%), and benzyl-l-homoserine (l-hSer(Bzl), 43%). More importantly, on this basis, it was also possible to distinguish fXIa from APC, thrombin, and fXa since they reject such derivatives.

The substrate specificity profile of fXIa at the P3 position was significantly different from those of APC, thrombin, and fXa. The well-recognized natural amino acids were the bulky l-Phe (52%), l-Trp (54%), and l-Tyr (37%), as well as the aliphatic structures of l-Leu (36%) and l-Nle (67%), which is consistent with the natural cleavage sequences in the fXIa substrate, factor IX.^28,29^ From the pool of unnatural analogs, a strong and selective preference was evident for l-2-aminooctanoic acid (l-2-Aoc, 100%) and l-dihydrotryptophan (l-Dht, 74%). We also observed that side-chain elongation enhanced substrate processing and its selectivity as in the case of l-homoleucine (l-hLeu, 68%), l-homophenylalanine (l-hPhe, 58%), and l-homocyclohexylalanine (l-hCha, 86%). Moreover, phenylalanine derivatives, mainly those with halogen substitutions in the meta position (Phe(3-F) (90%) and Phe(3-Cl) (79%)), had some of the highest relative activities in the case of fXIa, while they were poorly recognized by other coagulation factors.

The fXIa S4 pocket had broad substrate specificity but not for natural amino acids. Here, fXIa effectively recognized only branched aliphatic residues of l-Ile (43%) and l-Val (35%). We observed that this pocket could bind bulky amino acid residues with a benzyl group, namely, benzyloxycarbonyl-l-2,4-diaminobutyric acid (l-Dab(Z), 40%), 2-chlorobenzyloxycarbonyl-l-lysine (l-Lys(2-ClZ), 79%), benzyl-l-threonine (l-Thr(Bzl), 45%), and benzyl-l-tyrosine (l-Tyr(Bzl), 66%) with the champion 2,6-dichlorobenzyl-l-tyrosine (l-Tyr(2,6-Cl_2_-Z), 100%) being exclusively recognized by fXIa. Interestingly, all three enzymes other than fXIa ignored the aminobenzoic acid residues at this position, which also distinguished the specificity of fXIa from those of other coagulation proteases.

### Design and Kinetic Analysis of fXIa Selective Substrates

**Figure 4:**
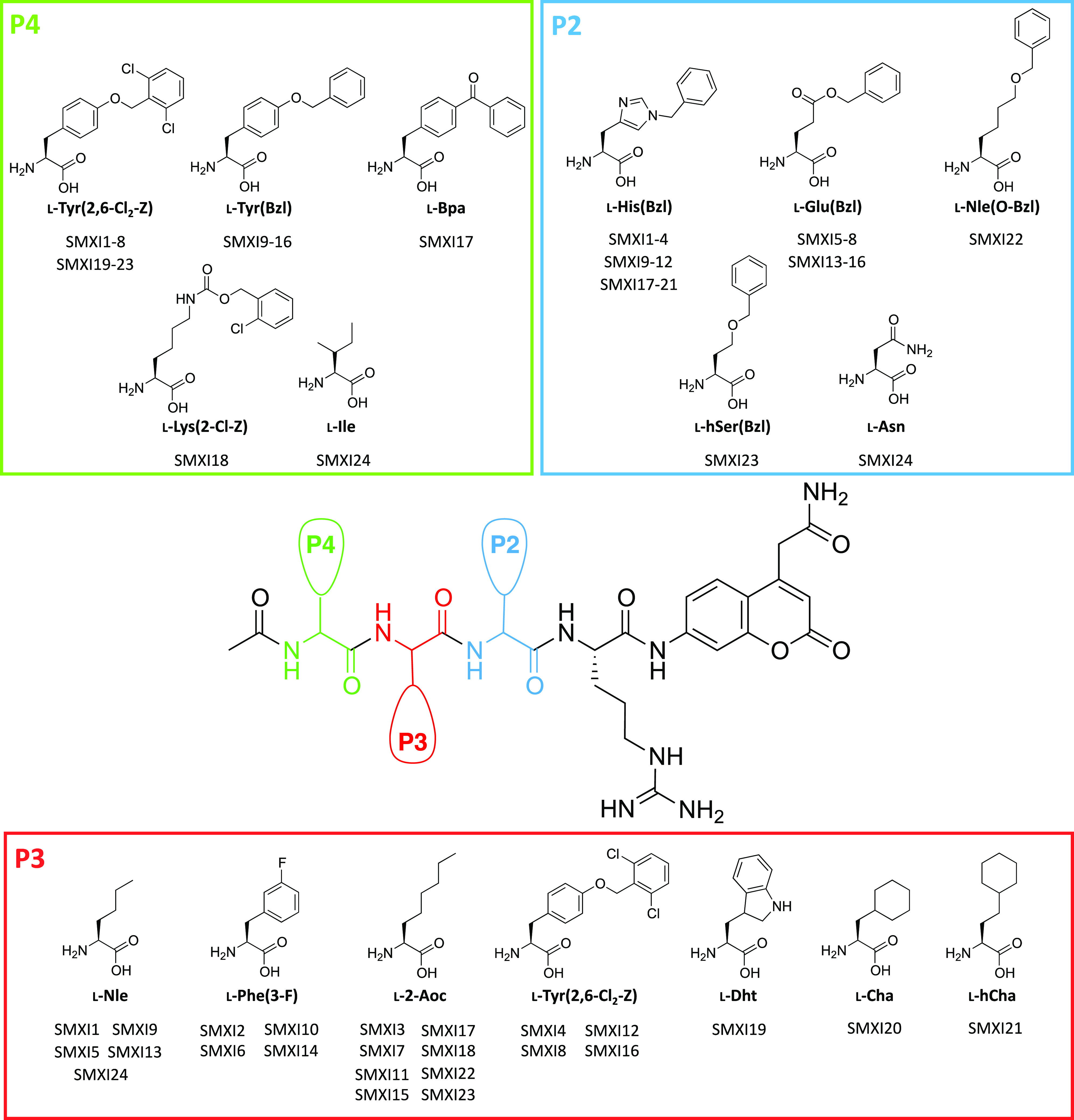
Design of fXIa ACC-labeled substrates. A general structure of an ACC-labeled substrate and amino acid residues selected for the synthesis of fXIa selective substrates based on library screening. Substrate sequences are presented in the supplemental section (Table S1).

**Figure 5:**
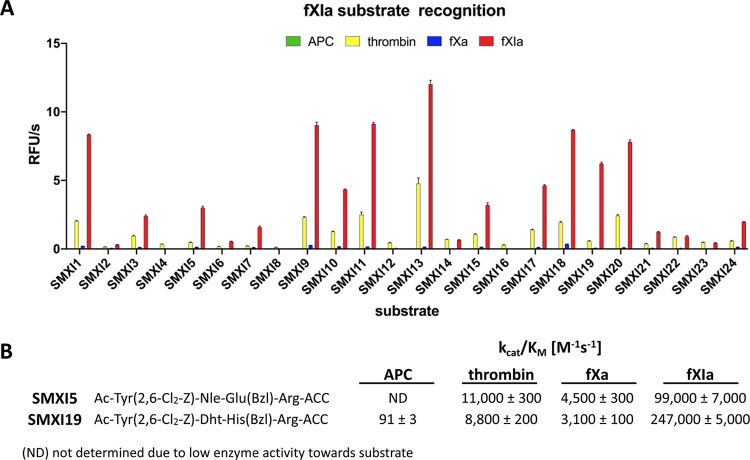
Analysis of fXIa substrates. (A) Substrate screening results with APC, thrombin, fXa, and fXIa. The rate of substrate hydrolysis as relative fluorescence units per second (RFU/s) was monitored for 30 min (λ_ex_ = 355 nm, λ_em_ = 460 nm). The data are presented as the mean values ± s.d.; *n* = 3, where *n* represents the number of independent experiments. Substrate sequences are presented in the supplemental section (Table S1). (B) Kinetic parameters (*k*_cat_/*K*_M_) of the most selective fXIa substrates toward four coagulation factors. The data represent the mean values ± s.d.; *n* = 3, where *n* is the number of independent experiments.

To validate the library screening results and to develop active and selective fXIa substrates, we combined the results from the P1 library screening with the HyCoSuL approach. To prevent cross-reactivity with other coagulation factors, we selected amino acid residues preferred only by fXIa and synthesized 23 (SMXI1-SMXI23) fluorogenic substrates with various P4-P2 regions (natural and unnatural amino acids), P1 l-Arg, ACC as a fluorophore and an acetylated N-terminus (Figure 4). As a reference, we synthesized one substrate (SMXI24) with only natural amino acid residues. At the P2 position, we selected amino acids with a benzyl group (l-His(Bzl), l-Glu(Bzl), l-Nle(O-Bzl), and l-hSer(Bzl)); at P3, we chose the aliphatic amino acids l-Nle, l-2-Aoc, l-Cha, and l-hCha and the bulky amino acids l-Phe(3-F), l-Tyr(2,6-Cl_2_-Z), and l-Dht; and at the P4 position, we incorporated the tyrosine and lysine derivatives (l-Tyr(2,6-Cl_2_-Z), l-Tyr(Bzl), l-Bpa, and l-Lys(2-Cl-Z)) (Figure 4). To assess selectivity over other coagulation factors, we performed an initial screening of all sequences with fXIa, APC, thrombin, and fXa (Figure 5A). For the most promising substrates, we performed a detailed kinetic analysis (*k*_cat_, *K*_M_, *k*_cat_/*K*_M_) (Figure 5B).

Our initial substrate screening showed that the exchange of only one amino acid at the P2 position, l-His(Bzl) (SMXI3) for l-Glu(Bzl) (SMXI7), l-Nle(O-Bzl) (SMXI22), or l-hSer(Bzl) (SMXI23), caused a significant decrease in activity (1.5-, 2.6-, and 5.5-fold, respectively) (Figure 5A). These results were in line with the HyCoSuL data where l-His(Bzl) was the best amino acid residue. Surprisingly, we noticed that the S3 subsite preference for longer aliphatic amino acid residues over their shorter analogs was not reflected in our substrate screening. Substrate analysis confirmed that sequences with l-Nle (SMXI1) or l-Cha (SMXI20) at the P3 position were more efficiently cleaved than those with l-2-Aoc (SMXI3) or l-hCha (SMXI21) with as much as a sixfold increase in overall activity. These findings revealed that the S3 pocket is small and plays an important role in substrate interactions. Additionally, tetrapeptides with halide-substituted phenylalanine or tyrosine analogs at the P3 position demonstrated little to no activity although library screening suggested that these amino acids were even better recognized than norleucine. We speculate that this is probably due to subsite cooperativity, which always needs to be considered when designing new peptide-based substrates. Moreover, we discovered that at the P4 position, l-Tyr(Bzl) (SMXI13) was more active on fXIa than l-Tyr(2,6-Cl_2_-Z) (SMXI5) (fourfold); however, the l-Tyr(2,6-Cl_2_-Z) residue showed significant selectivity over fXIa, reducing the cross-reactivity with other coagulation factors. Based on the substrate screening, we selected two lead sequences with the highest selectivity ratio toward fXIa, SMXI5 (Ac-Tyr(2,6-Cl_2_-Z)-Nle-Glu(Bzl)-Arg-ACC), and SMXI19 (Ac-Tyr(2,6-Cl_2_-Z)-Dht-His(Bzl)-Arg-ACC) for detailed kinetic evaluation (Figure 5B). The catalytic rates obtained for all investigated proteases demonstrated that SMXI19 was highly selective toward fXIa (*k*_cat_/*K*_M_ = 247,000 ± 5000 M^–1^ s^–1^) and was 28- and 80-fold better hydrolyzed by fXIa than by thrombin (*k*_cat_/*K*_M_ = 8800 ± 200 M^–1^ s^–1^) and fXa (*k*_cat_/*K*_M_ = 3100 ± 100 M^–1^ s^–1^), respectively. Furthermore, SMXI19 displayed a very high *k*_cat_/*K*_M_ selectivity ratio and almost no detectable hydrolysis when tested with APC (*k*_cat_/*K*_M_ = 91 ± 3 M^–1^ s^–1^). SMXI5 was also poorly recognized by APC, thrombin, and fXa, making both of these structures good candidates for conversion to inhibitors and ABPs.

### Development and Evaluation of fXIa Biotinylated ABPs

**Figure 6:**
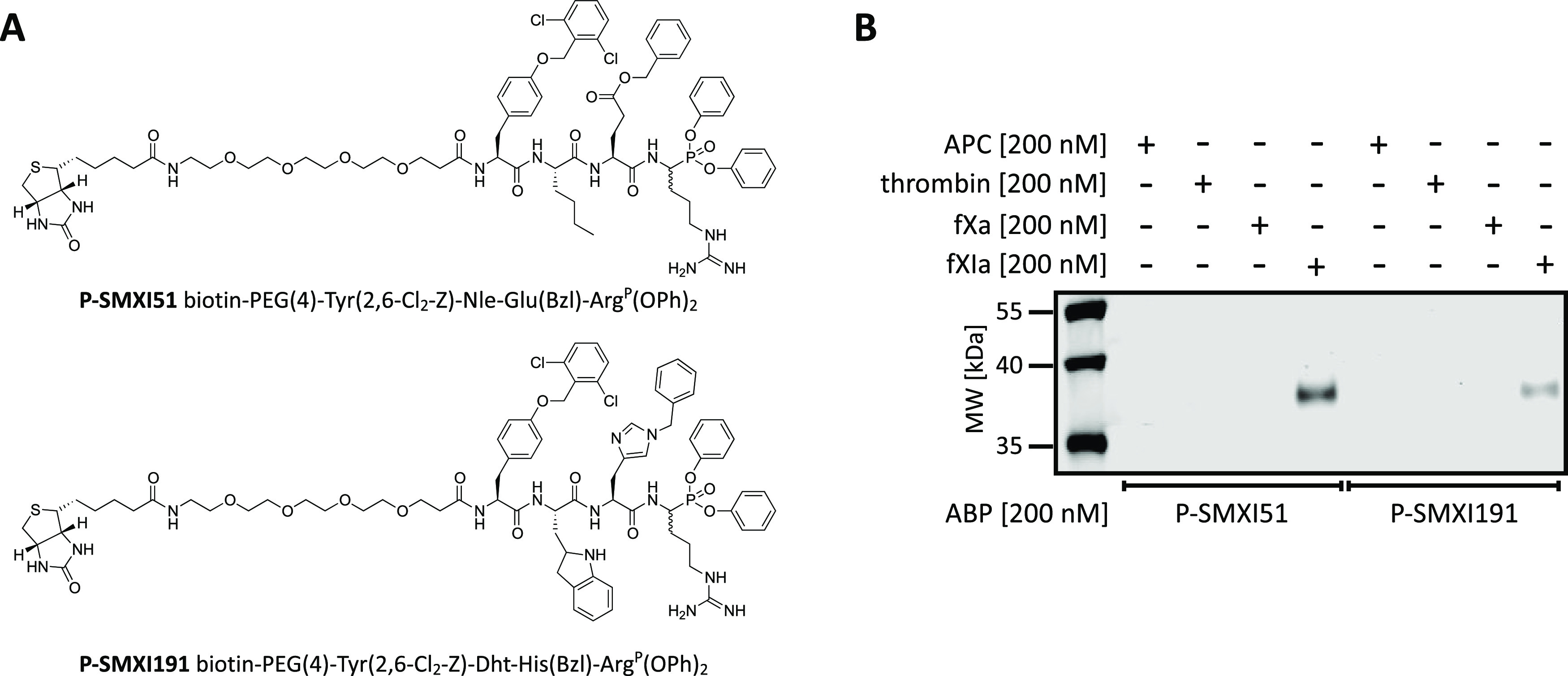
Biotinylated ABPs. (A) Structure of a biotinylated ABP designed for fXIa. (B) Labeling of purified coagulation factors (APC, thrombin, fXa, and fXIa) using two biotinylated ABPs. The enzymes (200 nM) were incubated separately with each probe (the probe/enzyme ratio was 1) for 30 min at 37 °C. Samples were then subjected to SDS–PAGE analysis, transferred to a membrane, incubated with fluorescent streptavidin Alexa Fluor 647 conjugate, and detected at 658 nm using an Azure Biosystems Sapphire Biomolecular Imager. The results are representative of at least three replicates.

Biotin-tagged ABPs are chemical tools frequently used in biological research since they are effective in isolating a protein of interest by affinity enrichment/purification on appropriate beads^37,38^ and have been successfully used to selectively label the active forms of various proteases in complex samples.^39−42^ The fXIa-selective substrate sequences SMXI5 and SMXI19 were used as scaffolds to create first-generation fXIa ABPs. With a mix of solid- and solution-phase methods, we synthesized two biotin-labeled probes: P-SMXI51 (biotin-PEG(4)-Tyr(2,6-Cl_2_-Z)-Nle-Glu(Bzl)-Arg^P^(OPh)_2_) and P-SMXI191 (biotin-PEG(4)-Tyr(2,6-Cl_2_-Z)-Dht-His(Bzl)-Arg^P^(OPh)_2_) (Figure 6A and Scheme S1). Both probes were equipped with the same affinity tag (biotin). We used polyethylene glycol (PEG(4)) as a linker to separate the peptide sequence from the biotin tag and improve the solubility of our probes. We used a diphenyl phosphonate as an electrophilic warhead since it is known to covalently bind in the active site of serine proteases.^43^ Next, we characterized the labeling of our biotinylated ABPs by incubating them separately with four purified coagulation factors, namely, APC, thrombin, fXa, and fXIa for 30 min (Figures 6B and S3). The probe/enzyme ratio was kept constant at 1:1. Next, we performed SDS–PAGE, nitrocellulose transfer and visualization with streptavidin conjugated with a fluorophore. Western blot analysis of P-SMXI51 and P-SMXI191 showed only one signal from a protein between 35 and 40 kDa, corresponding to a fXIa monomer. This result indicated a very high degree of selectivity of both ABPs toward fXIa. We observed that P-SMXI51 was more potent than P-SMXI191, making this sequence promising for use in further studies.

### FXIa Fluorescent ABP and Inhibitor Design and Characteristics

**Figure 7:**
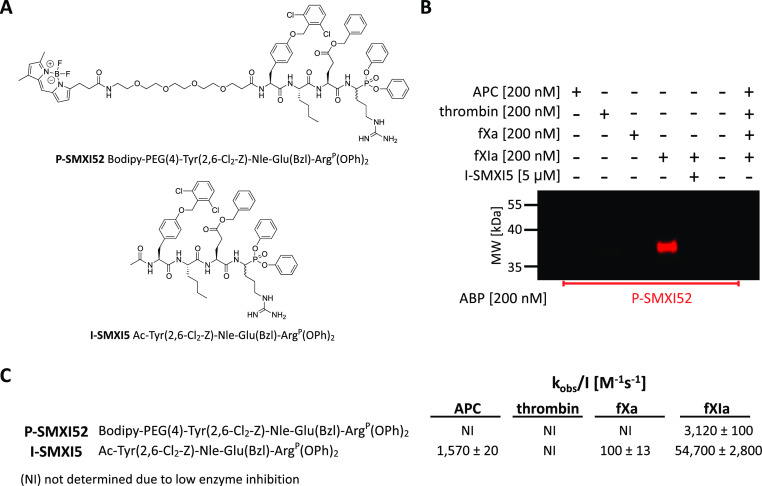
Fluorescent ABP and inhibitor. (A) Structures of fluorescent ABP and inhibitor designed for fXIa. (B) Labeling of purified coagulation factors (APC, thrombin, fXa, and fXIa) using fluorescent ABPs and fXIa inhibition. The enzymes (200 nM) were incubated separately with the probe (the probe/enzyme ratio was 1) for 30 min at 37 °C. Additionally, fXIa (200 nM) was incubated with its inhibitor (final inhibitor concentration 5 μM) for 60 min prior to probe addition (the probe/enzyme ratio was 1, 30 min). Samples were then subjected to electrophoresis and membrane transfer. Visualization was performed with a 488 nm laser using an Azure Biosystems Sapphire Biomolecular Imager. The results are representative of at least three replicates. (C) Kinetic parameters (*k*_obs_/*I*) of fluorescent ABP and inhibitor determined in the presence of four coagulation factors. The data represent the mean values ± s.d.; *n* = 3, where *n* is the number of independent experiments.

To create a fluorescent ABP, we exchanged the biotin tag for the BODIPY FL fluorophore. We synthesized a second-generation ABP with the general structure of BODIPY-PEG(4)-Tyr(2,6-Cl_2_-Z)-Nle-Glu(Bzl)-Arg^P^(OPh)_2_ (P-SMXI52) in addition to nonfluorescent version of the I-SMXI5 inhibitor (Ac-Tyr(2,6-Cl_2_-Z)-Nle-Glu(Bzl)-Arg^P^(OPh)_2_) (Figure 7A and Schemes S2 and S3). To evaluate the selectivity of our chemical tools, P-SMXI52 was incubated with fXIa and three other relevant serine proteases from the coagulation pathway (APC, thrombin, and fXa) at a 1:1 probe/enzyme ratio for 30 min (Figures 7B and S4). As controls, we used the probe and enzymes alone. One sample of fXIa was also preinhibited with 5 μM I-SMXI5 for 60 min prior to probe addition. After SDS–PAGE and western blot analysis, the membrane was scanned using the 488 nm channel. We observed only one fluorescent band generated by fXIa labeling and no off-target signals. Additionally, preblocking the active site of fXIa with the cold I-SMXI5 inhibitor completely inhibited the fluorescent signal. These results revealed that our ABP selectively binds to the fXIa active site of fXIa, supporting the use of P-SMXI52 for the detection of fXIa activity in complex biological samples.

### Fluorescent ABP and Inhibitor Kinetic Analysis

Apparent second-order rate constants were determined for fXIa inhibition (*k*_obs_(app)/*I*) under pseudo-first-order conditions for P-SMXI52 and I-SMXI5 in a concentration-dependent manner using the optimal substrate (Figure 7C). Taking into account the *K*_M_ of the substrate, we then calculated the substrate-independent *k*_obs_/*I* parameter. We observed that the exchange of the N-terminal acetyl for the BODIPY FL fluorophore resulted in a marked decrease in the *k*_obs_/*I* value from 54,700 ± 2800 M^–1^ s^–1^ for I-SMXI5 to 3120 ± 100 M^–1^ s^–1^ for P-SMXI52. One of the reasons may be the steric hindrance in the pockets distant from the enzyme active site caused by the large size of the BODIPY FL tag. However, P-SMXI52 retained its selectivity, as indicated by western blot and kinetic analysis of other enzymes from the coagulation pathway, making this sequence the first reported selective fluorescent ABP designed for fXIa.

### FXIa Detection in Human Plasma

**Figure 8:**
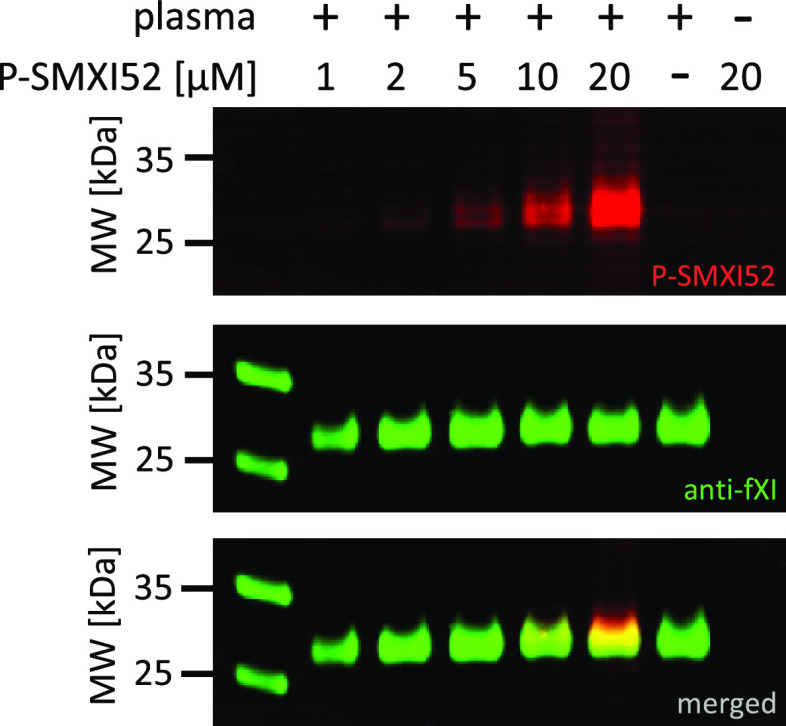
Probe concentration optimization assay. Human plasma was incubated with fluorescent ABP at various probe concentrations ranging from 1 to 20 μM for 60 min at 37 °C. Samples were then subjected to SDS–PAGE analysis, transferred to a membrane, immunostained with antibody, and imaged using an Azure Biosystems Sapphire Biomolecular Imager at 488 nm for BODIPY detection and at 658 nm for antibody detection. The results are representative of at least three replicates.

Previous labeling with purified coagulation factors showed that P-SMXI52 was potent for the detection of fXIa and displayed no off-target activity against other investigated proteases (APC, thrombin, and fXa). Thus, we assessed the utility of our probe in a more complex biological sample. For this purpose, we selected human plasma in which all blood coagulation factors are present.^44,45^ We incubated EDTA-chelated plasma with fluorescent ABP (P-SMXI52) in the 1–20 μM range for 1 h (Figures 8 and S5), followed by SDS–PAGE, protein transfer to nitrocellulose and immunostaining with antifXI. We observed a clear band starting from 2 μM ABP, which remained selective even at a high probe concentration (20 μM). These results demonstrated that P-SMXI52 bound to fXIa as confirmed by costaining with the antifXI antibody and could be used to distinguish fXIa from other coagulation factors in the complex system of human plasma.

## Discussion and Conclusions

Thrombotic diseases are a leading cause of preventable morbidity and mortality in developing and developed countries and are responsible for approximately 18 million deaths worldwide each year.^20,46^ Anticoagulation therapy is a mainstay of the prevention and treatment of thrombotic disorders; however, conventional anticoagulants (high- and low-molecular-weight heparins, vitamin K antagonists (VKAs), and direct fXa and thrombin inhibitors) trade antithrombotic benefits for bleeding risk.^21,47−49^ Therefore, there remains an unmet need for efficacious anticoagulant agents with reduced bleeding risk.^29,48,50^ Recent clinical studies implicate intrinsic pathway proteases located upstream in the coagulation cascade as alternative targets for safer anticoagulation. Among these proteases, fXI is of particular interest.^51,52^ To develop a better understanding of its role in both physiological and pathophysiological conditions and to aid in the development of novel therapeutic strategies, we created a set of potent and selective chemical tools for the easy labeling and detection of fXIa activity in biological samples.

The fXIa substrate specificity profile has been previously determined based on the recognition motif in physiological substrates^30,31^ and the crystal structure of the fXIa catalytic domain.^7^ However, these profiles include only natural amino acids, which significantly limits the development of selective chemical tools that can distinguish fXIa from other coagulation proteases. Therefore, in this work, we applied a defined (P1) library and the HyCoSuL (P4-P2) approach including a large collection of unnatural amino acids, which allowed a more extensive exploration of the chemical space in the P4-P1 positions. Previous studies reported that the fXIa S1 pocket is wide, deep, highly conserved and able to interact exclusively with positively charged arginine,^7,30,31^ which is also observed in the case of other serine proteases from the coagulation cascade.^33^ We found that in addition to arginine, the fXIa S1 subsite could also accommodate an unnatural phenylalanine derivative with a guanidine group in the *para* position, that is, l-Phe(guan) and several other amino acid residues (mostly phenylalanine derivatives). Our HyCoSuL screening showed that fXIa exhibited extremely narrow substrate specificity at the P2 position. The most preferred amino acid residues were those with a bulky benzyl group in their structure with l-His(Bzl) and l-Glu(Bzl) being the best hit in terms of activity and selectivity. The fXIa S3 subsite preferences were significantly different from those of APC, thrombin, and fXa, especially in the case of unnatural amino acids. The selection of l-Nle and l-Dht for the construction of tetrapeptide substrates allowed us to reduce cross-reactivity with other coagulation proteases. In the substrate sequence, the most preferred amino acid at the P4 position was l-Tyr(Bzl); however, l-Tyr(2,6-Cl_2_-Z) was the tyrosine derivative almost exclusively recognized by fXIa.

Equipped with a detailed picture of fXIa active site preferences, we synthesized a selection of fluorogenic tetrapeptide substrates better hydrolyzed by fXIa than by other coagulation factors. All designed selective substrates shared structural similarities, such as bulky tyrosine and lysine derivatives at the P4 position, aliphatic and bulky amino acids at the P3 positions, amino acids with a benzyl group in the P2 position, and arginine at the P1 position. We created two selective fXIa substrates (SMXI5, Ac-Tyr(2,6-Cl_2_-Z)-Nle-Glu(Bzl)-Arg-ACC and SMXI19, Ac-Tyr(2,6-Cl_2_-Z)-Dht-His(Bzl)-Arg-ACC), which displayed very high *k*_cat_/*K*_M_ selectivity ratios when tested with APC, thrombin, and fXa.

With recent evidence suggesting that the intrinsic pathway may play a significant role in thrombosis, fXIa has emerged as a promising target for novel anticoagulants, and focus has shifted to developing its selective inhibitors.^48,53^ Several pharmacologic strategies that aim to target fXIa have been discovered thus far, including antisense oligonucleotides (ASOs) and monoclonal antibodies (MAbs), which block fXIa activation or activity, and aptamers and small molecules (polypeptides, peptidomimetic active site inhibitors, polymeric GAGs, and their saccharide mimetics, nonpolymeric, and nonsaccharide GAG mimetics), which block the active site or induce allosteric modulation of the protease.^29,48,54,55^ Most of these agents are in early discovery and development phase; however, some of them have reached clinical trials. IONIS-416858, a specific fXI ASO was used in patients undergoing total knee arthroplasty and proved to be safe and effective against venous thrombosis with a limited risk of bleeding compared to the treatment with enoxaparin, the common pathway inhibitor.^48,54^ Other examples of compounds in advanced clinical trials are small molecule inhibitors, which include JNJ-70033093,^56^ BAY 2433334,^57^ as well as antibodies, such as osocimab,^58^ abelacimab,^59^ and xisomab.^52^

In this report, we describe the design and synthesis of P-SMXI152 (BODIPY-PEG(4)-Tyr(2,6-Cl_2_-Z)-Nle-Glu(Bzl)-Arg^P^(OPh)_2_), the first fluorescent ABP that is selective for fXIa over other coagulation proteases. We tested the utility of our ABP using kinetic assays and simple SDS–PAGE analysis with a set of purified coagulation factors and confirmed that ABP was selective for fXIa and not recognized by other coagulation factors, particularly thrombin, since it has the general trend of being a more active protease.^60^ In the last stage of this research, we used human plasma, a complex mixture of proteases, to test the selectivity of our fXIa ABP in a more biological setting. We incubated our fluorescent probe with the plasma sample and observed a clear band that was confirmed by antibody staining to be fXIa.

In summary, in this work, we provided in-depth profiling of fXIa substrate preferences at the P4-P1 positions. We developed a set of potent and selective chemical tools for fXIa investigation, such as substrates, inhibitors, and biotinylated and fluorescent ABPs. Current research focuses on understanding the biological role of the intrinsic system, and our tools may prove useful in helping to unveil fXIa functions in health and disease. Compounds described in this work can be applied for the pharmacological knockdown of fXIa and potentially provide safer anticoagulation while minimizing the risk of bleeding. Thus, they may be a viable alternative to vitamin K antagonists, heparins, and direct thrombin and fXa inhibitors. These compounds can also be used to monitor and visualize the level of fXIa in both physiological and pathophysiological conditions. In future, they may contribute as diagnostic tools and facilitate the choice of appropriate therapy for diseases like thrombosis, hemophilia C, ischemic stroke, myocardial infarction, and many others.

## Experimental Section

### Reagents

All chemical and biological reagents were purchased from commercial suppliers and used without further purification. Rink amide RA resin (particle size 200–300 mesh, loading 0.74 mM/g), 2-chlorotrityl chloride resin (particle size 100–200 mesh, loading 1.60 mM/g), Fmoc-6-ahx-OH, biotin, 1-[bis(dimethylamino)-methylene]-1*H*-1,2,3-triazolo[4,5-*b*]pyridinium 3-oxid hexafluorophosphate (HATU, peptide grade), piperidine (PIP, peptide grade), diisopropylcarbodiimide (DICI, peptide grade), *O*-benzotriazole-*N*,*N*,*N*′,*N*′-tetramethyluronium hexafluorophosphate (HBTU, peptide grade), and trifluoroacetic acid (TFA, purity 99%) were purchased from Iris Biotech GmbH (Marktredwitz, Germany). Fmoc-protected amino acids (purity >98%) were purchased from various suppliers: Iris Biotech GmbH, Creosalus (Louisville, KY, USA), P3 BioSystems (Louisville, KY, USA), and Bachem (Torrance, CA, USA). Triisopropylsilane (TIPS, purity 99%), 2,4,6-trimethylpyridine (2,4,6-collidine, peptide grade), and 2,2,2-trifluoroethanol (TFE) were all purchased from Sigma–Aldrich (Poznan, Poland). *N*,*N*-Diisopropylethylamine (DIPEA, peptide grade) was purchased from VWR International (Gdansk, Poland). P_2_O_5_ (phosphorus pentoxide, purity 98%) was purchased from Avantor (Gliwice, Poland). *N*-Hydroxybenzotriazole (HOBt, monohydrate, purity >98%) was purchased from Creosalus. The following solvents were purchased from Avantor: *N*,*N*′-dimethylformamide (DMF, peptide grade), dichloromethane (DCM, pure for analysis), methanol (MeOH, pure for analysis), diethyl ether (Et_2_O, pure for analysis), acetonitrile (ACN, HPLC grade), and AcOH (acetic acid, purity 99%). Streptavidin Alexa Fluor 647 conjugate (S21374) was purchased from Life Technologies (Eugene, OR, USA). The BODIPY FL fluorophore was purchased from Lumiprobe GmbH (Hannover, Germany). AntifXI antibody (sheep, polyclonal, PAHFXI-S) was purchased from Haematologic Technologies Inc. (Essex Junction, VT, USA).

Peptide substrates, ABPs, and inhibitor were purified by HPLC (Waters M600 solvent delivery module, Waters M2489 detector system, semipreparative Wide Pore C8 Discovery column (25 cm × 21.2 mm, 10 μm), Waters sp z.o.o., Warszawa, Poland). The solvent composition was as follows: phase A (water: 0.1% TFA) and phase B (acetonitrile: 0.1% TFA); gradient, from 95% A to 5% A over a period of 35 min; flow rate 10 mL/min. The purity of each compound was confirmed with an analytical HPLC system using a Discovery Bio Wide Pore C8 analytical column (25 cm × 4.6 mm, 5 μm). The solvent composition was as follows: water:0.1% TFA for phase A and acetonitrile:0.1% TFA for phase B; gradient, from 95% A to 5% A over a period of 15 min; flow rate 1 mL/min. The purity of all compounds was ≥95%. The molecular weight of each compound was confirmed by high-resolution mass spectrometry on a WATERS LCT premier XE with electrospray ionization (ESI) and a time-of-flight (TOF) module. For P-SMXI51, P-SMXI52, and I-SMXI5, the NMR analysis was performed using the Bruker Avance Neo spectrometer 600 MHz.

### Library Synthesis

Detailed protocols for the synthesis of the combinatorial library with Arg at the P1 position^32,35^ and the defined library Ac-Ala-Arg-Leu-P1-ACC are provided elsewhere.^34^ The synthesis of the fluorogenic leaving group ACC (7-amino-4-carbamoylmethylcoumarin) was carried out according to the method described by Maly et al.^61^

### Enzyme Preparation

Protein C, factor X, and prothrombin were purified from fresh frozen plasma and then activated as previously described.^33^

### Expression and Purification of Recombinant Human Factor XI

Full-length cDNA for human factor XI was ligated into the cloning site of a mammalian expression vector (pCEP4) containing the cytomegalovirus promoter. HEK293 cells (5 × 10^7^) expressing the Epstein–Barr virus nuclear antigen (EBNA) were transfected with 40 μg of factor XI/pCEP4 using lipofectamine 2000 (Invitrogen) according to the manufacturer’s instruction. Transfected cells were grown in DMEM with 10% fetal bovine serum and Pen/Strep/G418 (100 U/mL penicillin, 100 μg/mL streptomycin, 0.25 μg/mL amphotericin B, and 250 μg/mL G418) for 48 h and then switched to the same medium containing 200 μg/mL of hygromycin B. Media was exchanged every 48–96 h and hygromycin B resistant clones were transferred to 24-well tissue culture plates on day 10 to 14 of selection, and culture supernatants were tested for factor XI activity in a modified activated partial thromboplastin time assay (described below). Adherent clones expressing the highest level of recombinant protein were expanded into 525cm^2^ triple flasks. After reaching confluence, cells were washed with phosphate-buffered saline and 100 mL of CD-CHO serum free media (Thermofisher, 10743029) supplemented with Pen/Strep/G418 and 50 μg/mL hygromycin B was added to each flask. Media was harvested between 72 and 216 h in serum free expression media, and conditioned media was supplemented with benzamidine to 5 mmol/L and stored at −20 °C. Three liters of conditioned media was dialyzed against 50 mmol/L sodium acetate, pH 5.2, 150 mmol/L NaCl, 1 mmol/L EDTA, and loaded onto a 5 mL HiTrap SP HP cation exchange column (GE Healthcare). No factor XI activity was detected in the flow through by clotting assay (described below). The column was eluted with a linear NaCl gradient (150 to 1000 mmol/L), and factor XI containing fractions were pooled and dialyzed against 20 mmol/L Tris–HCl, pH 7.4, 100 mmol/L NaCl (Tris-buffered saline [TBS]). Dialysate was loaded onto a 5 mL HiTrap Heparin Sepharose affinity column (GE Healthcare) equilibrated with TBS and eluted with a linear NaCl gradient (100 to 1000 mmol/L). Factor XI containing fractions were pooled and concentrated to a final volume of 500 μL using an Vivaspin-20 concentrator (Sartorius). The preparation was then passed over a Superdex 200 16/60 gel filtration column (GE Healthcare) equilibrated with TBS, and 2 mL fractions were collected. Samples of each fraction were run on a 10% polyacrylamide-sodium dodecyl sulfate (SDS) gel under nonreducing conditions followed by staining with Coomassie brilliant blue. Fractions with pure factor XI were pooled, concentrated, and stored at −70 °C. Protein concentration was determined by measuring absorbance at 280 nm using an extinction coefficient (1%) for factor XI of 13.4.

### Plasma Assay for Factor XI Activity

Conditioned serum-free media and fractions from purification procedures were screened for factor XI activity by a modified activated partial thromboplastin time (aPTT) assay. Fifty microliters of human factor XI-deficient plasma (Haematologic Technologies) were mixed with 50 μL of the solution to be assayed and 50 μL of Thrombosil aPTT reagent. The mixture was incubated for 5 min at 37 °C, 50 μL of 25 mmol/L CaCl_2_ was added, and the time to fibrin clot formation was determined using a StartMax coagulometer (Stago). The factor XI concentration of undiluted pooled normal human plasma was considered to represent 100% activity (1 U factor XI/mL).

### Preparation and Activity of Recombinant fXIa

Purified recombinant human factor XI (250 μg/mL) was supplemented with 2.5 μg/mL human factor XIIa (BioVision) in TBS and incubated at 37 °C. Activation was confirmed by demonstrating complete conversion of the single chain zymogen to the two-chain active form on Coomassie blue-stained SDS-polyacrylamide gels run under reducing conditions. Factor XIIa was neutralized by the addition of corn trypsin inhibitor (CTI). Kinetic parameters for the cleavage of S-2366 (Chromogenix) by fXIa were determined. Briefly, 20 μL of fXIa at 5 μg/mL in TBS with 0.1% BSA (TBSA) was mixed with 75 μL of TBSA and 5 μL of 1.0 mg/mL CTI and incubated for 20 min at room temperature. The mixture was diluted to 900 μL with TBSA, and 100 μL of chromogenic substrate S-2366 at varying concentrations (50 to 1000 μmol/L final concentration) was added. Cleavage of S-2366 was followed by measuring the change in absorbance at 405 nm with a SpectraMax spectrophotometer (Molecular Devices). Michaelis–Menten constants (*K*_M_ and *V*_max_) for the cleavage of the chromogenic substrates were determined by standard methods. The value for *V*_max_ was converted to nanomolar *para*-nitroanaline (pNA) generated/s using an extinction coefficient for pNA of 9800 optical density (OD) units (405 nm)/mol pNA. Turn-over number (*k*_cat_) was calculated from the ratio of *V*_max_ to enzyme concentration.

### Kinetic Studies

All kinetic assays were performed using a spectrofluorometer (Molecular Devices SpectraMax Gemini XPS) on 96-well, white, flat bottom, nontreated plates (Corning). The parameters were as follows: excitation/emission wavelength, 355/460 nm (cutoff, 455 nm); varying concentrations of substrates, ABPs, inhibitor, and enzymes. The assay buffer (20 mM Tris-base, 150 mM NaCl, 5 mM CaCl_2_, pH 7.4) was prepared at room temperature, and the enzyme kinetic studies were performed at 37 °C. All enzymes were preincubated in assay buffer for 15 min at 37 °C before addition to the wells. Each assay was repeated at least twice, and the data represent the average of these repetitions. The obtained results were analyzed using SoftMax (Molecular Devices), GraphPad Prism, and Microsoft Excel software.

### Characterization of fXIa P1 Substrate Specificity

To determine fXIa preferences at the P1 position, we used the Ac-Ala-Arg-Leu-P1-ACC library of 133 individual fluorogenic substrates.^34^ The assay conditions were as follows: 0.5 μL of each library substrate was placed in one well, and 99.5 μL of preincubated enzyme (15 min, 37 °C) was added. The final library concentration was 100 μM, and the enzyme concentration was 8 nM. The release of ACC was measured for 30 min (λ_ex_ = 355 nm, λ_em_ = 460 nm), but only the linear portion of each progress curve was used to determine the substrate hydrolysis rate (RFU/s, relative fluorescence unit per second). The substrate specificity profile was established by setting the highest RFU/s value to 100% and adjusting the other results accordingly.

### Characterization of fXIa P4-P2 Substrate Specificity

The fXIa substrate specificity profile at the P4-P2 positions was determined using the HyCoSuL P1-Arg library comprising over 100 natural and unnatural amino acids in each position of three sublibraries (P4, P3, and P2).^32,35^ The libraries were each screened as follows: 1 μL of substrate and 99 μL of preincubated fXIa (15 min, 37 °C) with a final substrate mixture concentration of 100 μM and a final fXIa concentration of 8 nM. The substrate cleavage assay was carried out for 30 min (λ_ex_ = 355 nm, λ_em_ = 460 nm), and the linear part of each progress curve was used to determine the substrate hydrolysis rate. The results from screening analysis were based on the obtained RFU/s values for each sublibrary with the best recognized amino acid at each position set to 100% and other amino acids normalized accordingly.

### Synthesis of Individual Substrates

ACC-labeled fXIa tetrapeptide substrates were synthesized and purified as described elsewhere.^33^

### Screening of Individual Substrates

All potentially selective fXIa substrates were tested for their selectivity against four coagulation factors, APC, thrombin, fXa, and fXIa at a concentration of 1 μM. The assay conditions were as follows: 1 μL of each substrate was placed in separate wells on the plate followed by the addition of 99 μL of preincubated enzyme (15 min, 37 °C) in assay buffer. To obtain reliable results and a robust fluorescence signal, the final enzyme concentration was 120 nM for APC, 20 nM for thrombin, 70 nM for fXa, and 5 nM for fXIa. Substrate hydrolysis was measured for 30 min (λ_ex_ = 355 nm, λ_em_ = 460 nm), and the linear part of each progress curve was used to determine the substrate hydrolysis rate (RFU/s). The obtained RFU/s were then adjusted to represent the value corresponding to the same enzyme activity.

### Determination of Kinetic Parameters (*k*_cat_, *K*_M_, and *k*_cat_/*K*_M_) for Individual Substrates

The kinetic parameters (*k*_cat_, *K*_M_, and *k*_cat_/*K*_M_) of selected ACC-labeled substrates were determined using Michaelis–Menten nonlinear regression according to the protocol described by Poreba et al.^62^ Each substrate was serially diluted until the eighth well to obtain substrate concentrations ranging from 1.16 nM to 111 μM depending on the substrate used. Next, each enzyme was preincubated (15 min, 37 °C) in assay buffer to a final enzyme concentration of 425 nM for APC, 70 nM for thrombin, 200 nM for fXa, and 5 nM for fXIa and was added to the wells containing eight different substrate concentrations. The release of ACC was measured for 30 min (λ_ex_ = 355 nm, λ_em_ = 460 nm). The linear part of each progression curve was used to calculate the kinetic parameters using GraphPad Prism and Microsoft Excel software.

### Synthesis of the Inhibitor and ABPs

Irreversible inhibitor, biotin-labeled, and fluorescently labeled probes for fXIa were synthesized and purified as described previously for APC, thrombin, and fXa by Modrzycka et al.^33^

### Determination of Inhibition Kinetics (*k*_obs_/*I*) for the Inhibitor and Fluorescently Labeled ABP

The *k*_obs_/*I* parameters for APC, thrombin, fXa, and fXIa were measured under pseudo-first-order conditions. Inhibitor and ABP were serially diluted in assay buffer until the seventh well to obtain concentrations ranging from 308 nM to 50 μM. Then, 20 μL of selected substrate (154 μM SMA5 for APC, 100 μM SMA4 for thrombin, 50 μM SMII18 for fXa, and 100 μM SMX1 for fXIa)^33^ was added to the wells containing 20 μL of seven different ABP/inhibitor concentrations. Next, 60 μL of APC, thrombin, fXa, or fXIa (at a concentration of 10 nM) preincubated at 37 °C was added, and the fluorescence increase over time was measured (λ_ex_ = 355 nm, λ_em_ = 460 nm) for 30 min. The *k*_obs_/*I* parameters were calculated in GraphPad Prism and Microsoft Excel software.^41^

### Detection of fXIa with ABPs Based on SDS–PAGE Analysis

To determine biotin-labeled ABPs selectivity, four purified coagulation factors (APC, thrombin, fXa, and fXIa) with a constant concentration of 200 nM were incubated separately with each probe (the probe/enzyme ratio was 1) in assay buffer for 30 min at 37 °C. Each enzyme was incubated with each probe in a volume of 40 μL followed by reduction with 20 μL of 3 × SDS/DTT for 5 min at 95 °C. The first well was loaded with 0.5 μL of the protein marker PageRuler Prestained Protein Ladder (Thermo Scientific), and then 10 μL of each sample was run on a 12% (w/v) 15-well gel. SDS–PAGE separation was performed at 200 V for 39 min followed by a wet tank transfer to a nitrocellulose membrane (0.2 μm, Bio-Rad) at 10 V for 60 min. The membrane was blocked with 2.5% BSA in TBS-T (Tris-buffered saline with 0.1% (v/v) Tween 20) for 60 min at room temperature. Next, the membrane was incubated with fluorescent streptavidin Alexa Fluor 647 conjugate (dilution 1:10,000 in TBS-T with 1% BSA) for 60 min. The biotin-labeled ABPs were detected at 658 nm using an Azure Biosystems Sapphire Biomolecular Imager and Azure Spot Analysis software.

For fluorescently labeled probe sample preparation, electrophoresis and membrane transfer were performed as described above. When testing the inhibitor utility, 200 nM fXIa was incubated with inhibitor (final inhibitor concentration 5 μM) for 60 min prior to probe addition. After the membrane was blocked with 2.5% BSA in TBS-T (60 min), the labeled proteins were directly imaged with a 488 nM laser using an Azure Biosystems Sapphire Biomolecular Imager and Azure Spot Analysis software.

### Detection of fXIa in Human Plasma

Human plasma (collected in tubes containing anticoagulant EDTA) was isolated from whole blood as described elsewhere^33^ and incubated with a fluorescently labeled probe at probe concentrations ranging from 1 to 20 μM. Incubation was carried out in assay buffer for 60 min at 37 °C. Plasma was incubated with the probe in a total volume of 40 μL (20 μL of plasma and 20 μL of probe) followed by reduction with 20 μL of 3 × SDS/DTT for 5 min at 95 °C. The first well was loaded with 0.5 μL of the protein marker PageRuler Prestained Protein Ladder (Thermo Scientific), and then 5 μL of each sample was run on a 12% (w/v) 15-well gel. SDS–PAGE separation was performed at 200 V for 39 min followed by transfer to a nitrocellulose membrane (0.2 μm, Bio-Rad) at 10 V for 60 min. The membrane was blocked with 2.5% BSA in TBS-T for 60 min at room temperature. Next, the membrane was treated with sheep antihuman polyclonal fXI antibody (Haematologic Technologies Inc., PAHFXI-S, 1:1000) for 7 h followed by incubation with Alexa Fluor 680 donkey antisheep secondary antibody (Life Technologies, A21102, 2:10,000) for 1 h (both at room temperature). The membrane was then scanned using an Azure Biosystems Sapphire Biomolecular Imager and Azure Spot Analysis software for fXIa at 488 nm (for BODIPY detection) and 658 nm (for antibody detection).

### Safety Statement

No unexpected or unusually high safety hazards were encountered.

## Supporting Information Available

Substrate specificity profiles for APC, thrombin, fXa, and fXIa; ABPs and inhibitor synthesis; purity and MS analysis for substrates, inhibitor, and activity-based probes; NMR analysis of P-SMXI51, P-SMXI52, and I-SMXI5; and full-size blots (PDF)
Molecular formula strings (CSV)

## Abbreviations Used

ABP: activity-based probe
ACC: 7-amino-4-carbamoylmethylcoumarin
APC: activated protein C
fXa: factor Xa
fXIa: factor XIa
HyCoSuL: hybrid combinatorial substrate library
RFU: relative fluorescence unit

## References

[ref1] Furie B.; Furie B. C. The molecular basis of blood coagulation. Cell 1988, 53, 505–518. 10.1016/0092-8674(88)90567-3. 3286010

[ref2] Chaudhry R.; Usama S. M.; Babiker H. M. In StatPearls, 2022. 29489185

[ref3] Mackman N.; Tilley R. E.; Key N. S. Role of the extrinsic pathway of blood coagulation in hemostasis and thrombosis. Arterioscler., Thromb., Vasc. Biol. 2007, 27, 1687–1693. 10.1161/ATVBAHA.107.141911. 17556654

[ref4] Palta S.; Saroa R.; Palta A. Overview of the coagulation system. Indian J. Anaesth. 2014, 58, 515–523. 10.4103/0019-5049.144643. 25535411 PMC4260295

[ref5] Schmaier A. H. Physiologic activities of the contact activation system. Thromb. Res. 2014, 133, S41–S44. 10.1016/j.thromres.2014.03.018. 24759141 PMC4004333

[ref6] Lowenberg E. C.; Meijers J. C.; Monia B. P.; Levi M. Coagulation factor XI as a novel target for antithrombotic treatment. J. Thromb. Haemostasis 2010, 8, 2349–2357. 10.1111/j.1538-7836.2010.04031.x. 20727068

[ref7] Emsley J.; McEwan P. A.; Gailani D. Structure and function of factor XI. Blood 2010, 115, 2569–2577. 10.1182/blood-2009-09-199182. 20110423 PMC4828079

[ref8] Griffin J. H. Blood coagulation. The thrombin paradox. Nature 1995, 378, 337–338. 10.1038/378337a0. 7477366

[ref9] Esmon C. T. The protein C pathway. Chest 2003, 124, 26S–32S. 10.1378/chest.124.3_suppl.26s. 12970121

[ref10] Mohammed B. M.; Matafonov A.; Ivanov I.; Sun M. F.; Cheng Q.; Dickeson S. K.; Li C.; Sun D.; Verhamme I. M.; Emsley J.; Gailani D. An update on factor XI structure and function. Thromb. Res. 2018, 161, 94–105. 10.1016/j.thromres.2017.10.008. 29223926 PMC5776729

[ref11] Raskob G. E.; Angchaisuksiri P.; Blanco A. N.; Buller H.; Gallus A.; Hunt B. J.; Hylek E. M.; Kakkar A.; Konstantinides S. V.; McCumber M.; Ozaki Y.; Wendelboe A.; Weitz J. I.; Thrombosis: a major contributor to global disease burden. Arterioscler., Thromb., Vasc. Biol. 2014, 34, 2363–2371. 10.1161/ATVBAHA.114.304488. 25304324

[ref12] Berber E. Molecular characterization of FXI deficiency. Clin. Appl. Thromb./Hemostasis 2011, 17, 27–32. 10.1177/1076029609355587. 20308231

[ref13] Gotovac Jercic K.; Blazekovic A.; Hancevic M.; Bilic E.; Borovecki F. Congenital factor XI deficiency caused by a novel F11 missense variant: a case report. Croat. Med. J. 2020, 61, 62–65. 10.3325/cmj.2020.61.62. 32118380 PMC7063557

[ref14] Gailani D.; Gruber A. Factor XI as a Therapeutic Target. Arterioscler., Thromb., Vasc. Biol. 2016, 36, 1316–1322. 10.1161/ATVBAHA.116.306925. 27174099 PMC4919154

[ref15] Puy C.; Rigg R. A.; McCarty O. J. The hemostatic role of factor XI. Thromb. Res. 2016, 141, S8–S11. 10.1016/S0049-3848(16)30354-1. 27207433 PMC6135087

[ref16] Wang X.; Smith P. L.; Hsu M. Y.; Gailani D.; Schumacher W. A.; Ogletree M. L.; Seiffert D. A. Effects of factor XI deficiency on ferric chloride-induced vena cava thrombosis in mice. J. Thromb. Haemostasis 2006, 4, 1982–1988. 10.1111/j.1538-7836.2006.02093.x. 16961605

[ref17] Meijers J. C.; Tekelenburg W. L.; Bouma B. N.; Bertina R. M.; Rosendaal F. R. High levels of coagulation factor XI as a risk factor for venous thrombosis. N. Engl. J. Med. 2000, 342, 696–701. 10.1056/NEJM200003093421004. 10706899

[ref18] Cushman M.; O’Meara E. S.; Folsom A. R.; Heckbert S. R. Coagulation factors IX through XIII and the risk of future venous thrombosis: the Longitudinal Investigation of Thromboembolism Etiology. Blood 2009, 114, 2878–2883. 10.1182/blood-2009-05-219915. 19617576 PMC2756198

[ref19] Doggen C. J.; Rosendaal F. R.; Meijers J. C. Levels of intrinsic coagulation factors and the risk of myocardial infarction among men: Opposite and synergistic effects of factors XI and XII. Blood 2006, 108, 4045–4051. 10.1182/blood-2005-12-023697. 16931632

[ref20] Wendelboe A. M.; Raskob G. E. Global Burden of Thrombosis: Epidemiologic Aspects. Circ. Res. 2016, 118, 1340–1347. 10.1161/CIRCRESAHA.115.306841. 27126645

[ref21] Yeh C. H.; Hogg K.; Weitz J. I. Overview of the new oral anticoagulants: opportunities and challenges. Arterioscler., Thromb., Vasc. Biol. 2015, 35, 1056–1065. 10.1161/ATVBAHA.115.303397. 25792448

[ref22] Gailani D.; Bane C. E.; Gruber A. Factor XI and contact activation as targets for antithrombotic therapy. J. Thromb. Haemostasis 2015, 13, 1383–1395. 10.1111/jth.13005. 25976012 PMC4516614

[ref23] Muller F.; Gailani D.; Renne T. Factor XI and XII as antithrombotic targets. Curr. Opin. Hematol. 2011, 18, 349–355. 10.1097/MOH.0b013e3283497e61. 21730835 PMC4364027

[ref24] Gailani D.; Smith S. B. Structural and functional features of factor XI. J. Thromb. Haemostasis 2009, 7, 75–78. 10.1111/j.1538-7836.2009.03414.x. 19630773 PMC2849299

[ref25] Walsh P. N.; Baglia F. A.; Jameson B. A. Factor XI: structure-function relationships utilizing monoclonal antibodies protein modification, computational chemistry, and rational synthetic peptide design. Methods Enzymol. 1993, 222, 65–96. 10.1016/0076-6879(93)22008-4. 8412816

[ref26] Gailani D.; Emsley J. Toward a better understanding of factor XI activation. J. Thromb. Haemostasis 2019, 17, 2016–2018. 10.1111/jth.14631. 31797540 PMC8559519

[ref27] Gailani D.; Broze G. J. Jr. Factor XI activation in a revised model of blood coagulation. Science 1991, 253, 909–912. 10.1126/science.1652157. 1652157

[ref28] Schaefer M.; Buchmueller A.; Dittmer F.; Strassburger J.; Wilmen A. Allosteric Inhibition as a New Mode of Action for BAY 1213790, a Neutralizing Antibody Targeting the Activated Form of Coagulation Factor XI. J. Mol. Biol. 2019, 431, 4817–4833. 10.1016/j.jmb.2019.09.008. 31655039

[ref29] Al-Horani R. A.; Afosah D. K. Recent advances in the discovery and development of factor XI/XIa inhibitors. Med. Res. Rev. 2018, 38, 1974–2023. 10.1002/med.21503. 29727017 PMC6173998

[ref30] Katayama K.; Ericsson L. H.; Enfield D. L.; Walsh K. A.; Neurath H.; Davie E. W.; Titani K. Comparison of amino acid sequence of bovine coagulation Factor IX (Christmas Factor) with that of other vitamin K-dependent plasma proteins. Proc. Natl. Acad. Sci. U. S. A. 1979, 76, 4990–4994. 10.1073/pnas.76.10.4990. 291916 PMC413064

[ref31] McRae B. J.; Kurachi K.; Heimark R. L.; Fujikawa K.; Davie E. W.; Powers J. C. Mapping the active sites of bovine thrombin, factor IXa, factor Xa, factor XIa, factor XIIa, plasma kallikrein, and trypsin with amino acid and peptide thioesters: development of new sensitive substrates. Biochemistry 1981, 20, 7196–7206. 10.1021/bi00528a022. 6976185

[ref32] Poreba M.; Salvesen G. S.; Drag M. Synthesis of a HyCoSuL peptide substrate library to dissect protease substrate specificity. Nat. Protoc. 2017, 12, 2189–2214. 10.1038/nprot.2017.091. 28933778

[ref33] Modrzycka S.; Kolt S.; Polderdijk S. G. I.; Adams T. E.; Potoczek S.; Huntington J. A.; Kasperkiewicz P.; Drag M. Parallel imaging of coagulation pathway proteases activated protein C, thrombin, and factor Xa in human plasma. Chem. Sci. 2022, 13, 6813–6829. 10.1039/d2sc01108e. 35774156 PMC9200056

[ref34] Rut W.; Zhang L.; Kasperkiewicz P.; Poreba M.; Hilgenfeld R.; Drag M. Extended substrate specificity and first potent irreversible inhibitor/activity-based probe design for Zika virus NS2B-NS3 protease. Antiviral Res. 2017, 139, 88–94. 10.1016/j.antiviral.2016.12.018. 28034744

[ref35] Kasperkiewicz P.; Poreba M.; Snipas S. J.; Lin S. J.; Kirchhofer D.; Salvesen G. S.; Drag M. Design of a Selective Substrate and Activity Based Probe for Human Neutrophil Serine Protease 4. PLoS One 2015, 10, e0132818 10.1371/journal.pone.0132818. 26172376 PMC4501687

[ref36] Rut W.; Groborz K.; Zhang L.; Modrzycka S.; Poreba M.; Hilgenfeld R.; Drag M. Profiling of flaviviral NS2B-NS3 protease specificity provides a structural basis for the development of selective chemical tools that differentiate Dengue from Zika and West Nile viruses. Antiviral Res. 2020, 175, 104731 10.1016/j.antiviral.2020.104731. 32014497

[ref37] Sanman L. E.; Bogyo M. Activity-based profiling of proteases. Annu. Rev. Biochem. 2014, 83, 249–273. 10.1146/annurev-biochem-060713-035352. 24905783

[ref38] Wright M. H.; Sieber S. A. Chemical proteomics approaches for identifying the cellular targets of natural products. Nat. Prod. Rep. 2016, 33, 681–708. 10.1039/c6np00001k. 27098809 PMC5063044

[ref39] Janiszewski T.; Kolt S.; Kaiserman D.; Snipas S. J.; Li S.; Kulbacka J.; Saczko J.; Bovenschen N.; Salvesen G.; Drag M.; Bird P. I.; Kasperkiewicz P. Noninvasive optical detection of granzyme B from natural killer cells with enzyme-activated fluorogenic probes. J. Biol. Chem. 2020, 295, 9567–9582. 10.1074/jbc.RA120.013204. 32439802 PMC7363135

[ref40] Poreba M.; Rut W.; Vizovisek M.; Groborz K.; Kasperkiewicz P.; Finlay D.; Vuori K.; Turk D.; Turk B.; Salvesen G. S.; Drag M. Selective imaging of cathepsin L in breast cancer by fluorescent activity-based probes. Chem. Sci. 2018, 9, 2113–2129. 10.1039/c7sc04303a. 29719685 PMC5896380

[ref41] Kasperkiewicz P.; Poreba M.; Snipas S. J.; Parker H.; Winterbourn C. C.; Salvesen G. S.; Drag M. Design of ultrasensitive probes for human neutrophil elastase through hybrid combinatorial substrate library profiling. Proc. Natl. Acad. Sci. U. S. A. 2014, 111, 2518–2523. 10.1073/pnas.1318548111. 24550277 PMC3932852

[ref42] Rut W.; Poreba M.; Kasperkiewicz P.; Snipas S. J.; Drag M. Selective Substrates and Activity-Based Probes for Imaging of the Human Constitutive 20S Proteasome in Cells and Blood Samples. J. Med. Chem. 2018, 61, 5222–5234. 10.1021/acs.jmedchem.8b00026. 29806773

[ref43] Powers J. C.; Asgian J. L.; Ekici O. D.; James K. E. Irreversible inhibitors of serine, cysteine, and threonine proteases. Chem. Rev. 2002, 102, 4639–4750. 10.1021/cr010182v. 12475205

[ref44] Suttie J. W. Synthesis of vitamin K-dependent proteins. FASEB J. 1993, 7, 445–452. 10.1096/fasebj.7.5.8462786. 8462786

[ref45] Hunfeld A.; Etscheid M.; Konig H.; Seitz R.; Dodt J. Detection of a novel plasma serine protease during purification of vitamin K-dependent coagulation factors. FEBS Lett. 1999, 456, 290–294. 10.1016/s0014-5793(99)00959-x. 10456326

[ref46] Raskob G. E.; Angchaisuksiri P.; Blanco A. N.; Buller H.; Gallus A.; Hunt B. J.; Hylek E. M.; Kakkar T. L.; Konstantinides S. V.; McCumber M.; Ozaki Y.; Wendelboe A.; Weitz J. I.; Thrombosis: a major contributor to global disease burden. Semin. Thromb. Hemostasis 2014, 40, 724–735. 10.1055/s-0034-1390325. 25302681

[ref47] Franchini M.; Liumbruno G. M.; Bonfanti C.; Lippi G. The evolution of anticoagulant therapy. Blood Transfus. 2016, 14, 175–184. 10.2450/2015.0096-15. 26710352 PMC4781787

[ref48] Szekely O.; Borgi M.; Lip G. Y. H. Factor XI inhibition fulfilling the optimal expectations for ideal anticoagulation. Expert Opin. Emerging Drugs 2019, 24, 55–61. 10.1080/14728214.2019.1591368. 30845846

[ref49] De Caterina R.; Husted S.; Wallentin L.; Andreotti F.; Arnesen H.; Bachmann F.; Baigent C.; Huber K.; Jespersen J.; Kristensen S. D.; Lip G. Y.; Morais J.; Rasmussen L. H.; Siegbahn A.; Verheugt F. W.; Weitz J. I.; General mechanisms of coagulation and targets of anticoagulants (Section I). Position Paper of the ESC Working Group on Thrombosis--Task Force on Anticoagulants in Heart Disease. Thromb. Haemostasis 2013, 109, 569–579. 10.1160/TH12-10-0772. 23447024

[ref50] Fredenburgh J. C.; Gross P. L.; Weitz J. I. Emerging anticoagulant strategies. Blood 2017, 129, 147–154. 10.1182/blood-2016-09-692996. 27780803

[ref51] Weitz J. I.; Fredenburgh J. C. 2017 Scientific Sessions Sol Sherry Distinguished Lecture in Thrombosis: Factor XI as a Target for New Anticoagulants. Arterioscler., Thromb., Vasc. Biol. 2018, 38, 304–310. 10.1161/ATVBAHA.117.309664. 29269514

[ref52] Lorentz C. U.; Verbout N. G.; Wallisch M.; Hagen M. W.; Shatzel J. J.; Olson S. R.; Puy C.; Hinds M. T.; McCarty O. J. T.; Gailani D.; Gruber A.; Tucker E. I. Contact Activation Inhibitor and Factor XI Antibody, AB023, Produces Safe, Dose-Dependent Anticoagulation in a Phase 1 First-In-Human Trial. Arterioscler., Thromb., Vasc. Biol. 2019, 39, 799–809. 10.1161/ATVBAHA.118.312328. 30700130 PMC6494446

[ref53] Mackman N. Triggers, targets and treatments for thrombosis. Nature 2008, 451, 914–918. 10.1038/nature06797. 18288180 PMC2848509

[ref54] Buller H. R.; Bethune C.; Bhanot S.; Gailani D.; Monia B. P.; Raskob G. E.; Segers A.; Verhamme P.; Weitz J. I.; Investigators F.-A. T. Factor XI antisense oligonucleotide for prevention of venous thrombosis. N. Engl. J. Med. 2015, 372, 232–240. 10.1056/NEJMoa1405760. 25482425 PMC4367537

[ref55] Bickmann J. K.; Baglin T.; Meijers J. C. M.; Renne T. Novel targets for anticoagulants lacking bleeding risk. Curr. Opin. Hematol. 2017, 24, 419–426. 10.1097/MOH.0000000000000367. 28731874

[ref56] Weitz J. I.; Strony J.; Ageno W.; Gailani D.; Hylek E. M.; Lassen M. R.; Mahaffey K. W.; Notani R. S.; Roberts R.; Segers A.; Raskob G. E.; Investigators A.-T. Milvexian for the Prevention of Venous Thromboembolism. N. Engl. J. Med. 2021, 385, 2161–2172. 10.1056/NEJMoa2113194. 34780683 PMC9540352

[ref57] Piccini J. P.; Caso V.; Connolly S. J.; Fox K. A. A.; Oldgren J.; Jones W. S.; Gorog D. A.; Durdil V.; Viethen T.; Neumann C.; Mundl H.; Patel M. R.; Auer J.; Hubauer M.; Pandzic S.; Preishuber E.; Primus-Grabscheit C.; Reitgruber D.; Schmalzer F.; Adlbrecht C.; Schober A.; Hajos J.; Keil C.; Schratter A.; Frick M.; Benda M. A.; Mächler M.; Mutschlechner B.; Saely C.; Sprenger L.; Lichtenauer M.; Eber M.; Hoppe U.; Kolbitsch T.; Jirak P. M.; Mirna M.; Schönbauer R.; Bergler-Klein J.; Hengstenberg C.; Stojkovic S.; Scherr D.; Manninger-Wünscher M.; Rohrer U.; Stühlinger M.; Schgoer W.; Schwarzl J.; Pürerfellner H.; Derndorfer M.; Ebner C.; Eder V.; Kollias G.; Sturmberger T.; Sieghartsleitner S.; Vijgen J.; Koopman P.; Dujardin K.; Anné W.; De Ceuninck M.; Tavernier R.; Duytschaever M.; Knecht S.; Missault L.; Vandekerckhove Y.; Rossenbacker T.; Ector B.; Charlier F.; Debruyne P.; Dewilde W.; Janssens L.; Roosen J.; Vankelecom B.; Heidbuchel H.; Delesie M.; Vervoort G.; Rombouts H.; Vanassche T.; Engelen M.; Verhamme P.; Willems R.; Constance C.; Pranno N.; Cox J.; Bata I.; Macle L.; Aguilar M.; Tourigny J. C.; Dubuc M.; Dyrda K.; Guerra P.; Khairy P.; Mondésert B.; Rivard L.; Roy D.; Tadros R.; Talajic M.; Thibault B.; Nault I.; Blier L.; Champagne J.; Molin F.; O’Hara G.; Philippon F.; Plourde B.; Sarrazin J.-F.; Steinberg C.; Coufal Z.; Balazsik D.; Mikulica M.; Zapeca J.; Cermak O.; Drasnar T.; Falc M.; Hornof J.; Racz B.; Weissova D.; Linkova H.; Paskova E.; Petr R.; Sirakova A.; Kettner J.; Benak A.; Holek M.; Podpera I.; Podperova M.; Vancura V.; Jandik T.; Smid J.; Dedek V.; Banik J.; Durdil V.; Hnat T.; Lellouche N.; Rouffiac S.; Taldir G.; Bridonneau V.; Couffon P.; Daudin M.; Hamon C.; Lacaze J.; Quentin A.; Thebault C.; Boiffard E.; Billon O.; Miette F.; Pouliquen H.; Turlotte G.; Gorka H.; Albert F.; Bayle S.; Bensaid R.; Dasoveanu M.; Demichili T.; Dutoiu T.; Khalil C.; Loghin C.; Range G.; Roussel L.; Socié P.; Thuaire C.; Extramiana F.; Algalarrondo V.; Boughanmi H.; El Mansour N.; Mohammad U.; Sellier R.; Elbaz M.; Laperche C.; Maury P.; Kiss R.; Borsanyi T.; Gingl Z.; Polgar B.; Benczur B.; Bodor A.; Hepp T.; Malati E.; Nagy L.; Erdei N.; Kapus J.; Kapus K.; Toth B.; Matoltsy A.; Kiss T.; Merkely B.; Herczeg S.; Kiss O.; Sallo Z.; Toth K.; Habon T.; Rabai M.; Totsimon K.; Zilahi Z.; Bencze G.; Santa J.; Aradi D.; Kelemen B.; Bolognese L.; Nesti M.; Notarstefano P. G.; D’Orazio S.; Cosmi F.; Becattini C.; Agnelli G.; Broccatelli B.; Mosconi M. G.; Paciaroni M.; Urbini C.; Parato V. M.; Notaristefani C.; Scarano M.; Ameri P.; Ghigliotti G.; Guglielmi G.; Lotti R.; Merlo A. C.; Muiesan M. L.; Abondio A.; Berasi C.; Mattiuzzo E.; Mutti C.; Salvetti M.; Pignatelli P.; Menichelli D.; Pastori D.; Tamiya E.; Matsumoto T.; Takabe T.; Yamamoto S.; Yamashita H.; Higashiue S.; Furuya O.; Hiramatsu N.; Kasuga K.; Kojima S.; Komooka M.; Kuroyanagi S.; Matsuura M.; Takemoto T.; Yamamoto S.; Saito K.; Abe T.; Ishida I.; Iwanami Y.; Kataoka S.; Moriyama T.; Murohashi A.; Sasaki A.; Nakamura Y.; Ueno T.; Shimane A.; Hamana T.; Ichibori H.; Inoue T.; Itoh M.; Iwane S.; Kawai H.; Kokawa T.; Masumoto A.; Matsuo K.; Miyata T.; Nakano S.; Oishi S.; Onishi T.; Sawada T.; Saito T.; Shoda M.; Takahashi N.; Takaya T.; Taniguchi Y.; Tsukamoto S.; Tsukishiro Y.; Tsukiyama Y.; Tsunamoto H.; Uzu K.; Yamamoto H.; Yamamoto T.; Yokoi K.; Yoshida C.; Watanabe N.; Betsuyaku T.; Adachi K.; Awane K.; Goto D.; Sakakibara M.; Watanabe M.; Ueno H.; Hiroe Y.; Matsuo K.; Ayata K.; Fukuda K.; Hata Y.; Hashimoto K.; Matsumi H.; Nikaido A.; Okamoto S.; Sime I.; Stirna V.; Reinholde I.; Hansone S.; Kozlovska A.; Romanova J.; Klincare D.; Pontaga N.; Dirmans I.; Kalnins A.; Upite D.; Gersamija A.; Teleznikovs A.; Rozkova N.; Safro J.; Anguera Camós I.; Domenico Dallaglio P.; Salguero Bodes R.; Arnbas F.; Borrego L.; Marco A.; Jimenez J. R.; Gómez-Doblas J. J.; Pérez Cabeza A.; Ferreira Gonzålez I.; Limeres Freire J.; Lopez Grau M.; Viñolas Prat X.; Moreno Weidmann Z.; Guerra Ramos J. M.; Alonso Martin M. C.; Campos Garcia B.; Mogro Carranza J. M.; Mendez Zurita F. J.; Rodriguez Font E.; Gonzales Matos C. E.; García Hernando V.; Lindholm C.-J.; Thulin J.; Wallén H.; Hagwall K.; Eliasson K.; Lundvall M.; Olsson J.; Kjellman B.; Lind M.; Johansson L.; Svedberg N.; Berglund S.; Söderberg J.; Zedigh C.; Mooe T.; Axelsson M.; Binsell E.; Huber D.; Müller C.; Danier I.; Kühne M.; Okamura B.; Schoepfer H.; Simmen C.; Reichlin T.; Chollet L.; Lam A.; Wittmer S.; Rickli H.; Gall C.; Hametner G.; Intorp S.; Luescher D.; Haegeli L.; Berg J. C.; Ebrahimi R.; Auricchio A.; Crljenica C.; Moccetti T.; Monti C.; Pasotti E.; Petrova I.; Rossi M.; Mach F.; Namdar M.; de Groot J.; Proost V.; Neefs J.; Linz D.; van Stipdonk T.; den Uijl D.; Alings M.; Schaap J.; Segers D.; Wouters N.; Bartels L.; Tieleman R.; Pisters R.; de Vries T.; Selig J.; Kuijper A.; Bot P.; Keijzers M.; Verdel G.; Tukkie R.; van den Bos E.; Kauer F.; Oemrawsingh R.; Stevenhagen J.; van Es J.; Lip G.; Gupta D.; Kotalczyk A.; Gunstone A.; Brixey R. D.; Gorog D.; Dinarvand D.; Gue Y.; Kanji R.; Memtsas V.; Senior R.; Bioh G.; Wong Y.-K.; Child N. Safety of the oral factor XIa inhibitor asundexian compared with apixaban in patients with atrial fibrillation (PACIFIC-AF): a multicentre, randomised, double-blind, double-dummy, dose-finding phase 2 study. Lancet 2022, 399, 1383–1390. 10.1016/s0140-6736(22)00456-1. 35385695

[ref58] Weitz J. I.; Bauersachs R.; Becker B.; Berkowitz S. D.; Freitas M. C. S.; Lassen M. R.; Metzig C.; Raskob G. E. Effect of Osocimab in Preventing Venous Thromboembolism Among Patients Undergoing Knee Arthroplasty: The FOXTROT Randomized Clinical Trial. JAMA 2020, 323, 130–139. 10.1001/jama.2019.20687. 31935028 PMC6990695

[ref59] Koch A. W.; Schiering N.; Melkko S.; Ewert S.; Salter J.; Zhang Y.; McCormack P.; Yu J.; Huang X.; Chiu Y. H.; Chen Z.; Schleeger S.; Horny G.; DiPetrillo K.; Muller L.; Hein A.; Villard F.; Scharenberg M.; Ramage P.; Hassiepen U.; Cote S.; DeGagne J.; Krantz C.; Eder J.; Stoll B.; Kulmatycki K.; Feldman D. L.; Hoffmann P.; Basson C. T.; Frost R. J. A.; Khder Y. MAA868, a novel FXI antibody with a unique binding mode, shows durable effects on markers of anticoagulation in humans. Blood 2019, 133, 1507–1516. 10.1182/blood-2018-10-880849. 30692123

[ref60] Mather T.; Oganessyan V.; Hof P.; Huber R.; Foundling S.; Esmon C.; Bode W. The 2.8 A crystal structure of Gla-domainless activated protein C. EMBO J. 1996, 15, 6822–6831. 10.1002/j.1460-2075.1996.tb01073.x. 9003757 PMC452507

[ref61] Maly D. J.; Leonetti F.; Backes B. J.; Dauber D. S.; Harris J. L.; Craik C. S.; Ellman J. A. Expedient solid-phase synthesis of fluorogenic protease substrates using the 7-amino-4-carbamoylmethylcoumarin (ACC) fluorophore. J. Org. Chem. 2002, 67, 910–915. 10.1021/jo016140o. 11856036

[ref62] Poreba M.; Szalek A.; Kasperkiewicz P.; Drag M. Positional scanning substrate combinatorial library (PS-SCL) approach to define caspase substrate specificity. Methods Mol. Biol. 2014, 1133, 41–59. 10.1007/978-1-4939-0357-3_2. 24567093

