# Supplementary material for: Fluorescent Activity-Based Probe To Image and Inhibit Factor XIa Activity in Human Plasma
Source: J Med Chem. 2023 Mar 10;66(6):3785–97. doi: 10.1021/acs.jmedchem.2c00845 (PMC10041521; doi:10.1021/acs.jmedchem.2c00845)
Supplement: Supplementary file 1 — jm2c00845_si_001.pdf [file jm2c00845_si_001.pdf]

Supporting information

Fluorescent activity-based probe to image and inhibit factor XIa activity in human plasma

Sylwia Modrzycka[1], Sonia Kołt[1], Ty E. Adams[2], Stanisław Potoczek[3], James A. Huntington[2], Paulina Kasperkiewicz[1], and Marcin Drąg[1,*]

1 Department of Chemical Biology and Bioimaging, Faculty of Chemistry, Wrocław University of Science and Technology, Wybrzeże Wyspiańskiego 27, 50-370 Wrocław, Poland

2 Department of Haematology, Cambridge Institute for Medical Research, University of Cambridge, The Keith Peters Building, Hills Road, Cambridge, CB2 0XY, United Kingdom

3 Department of Haematology, Blood Neoplasms, and Bone Marrow Transplantation, Wrocław Medical University, Pasteura 1, 50-367 Wrocław, Poland

* corresponding author: marcin.drag@pwr.edu.pl

Table of Contents

1. Substrate specificity profiles for APC, thrombin, fXa, and fXIa S2
2. ABPs and inhibitor synthesis S4
3. Purity and MS analysis of fXIa compounds S7
4. NMR analysis of P-SMXI51, P-SMXI52, and I-SMXI5 S40
5. Full-size blots S48
6. References S49

Substrate specificity profiles for APC, thrombin, fXa, and fXIa

To determine the substrate specificity of fXIa at the P1 position, we used the defined library with the general structure of Ac-Ala-Arg-Leu-P1-ACC containing 133 individual fluorogenic substrates. This tailored library of fluorogenic substrates consists of a fixed P4-P2 motif (Ala-Arg-Leu) and various amino acid residues at the P1 position. The general structure of the library was Ac-Ala-Arg-Leu-P1-ACC, in which ACC was 7-amino-4-carbamoylmethylcoumarin, Ac was an acetyl group, and P1 was an individual natural or unnatural amino acid.

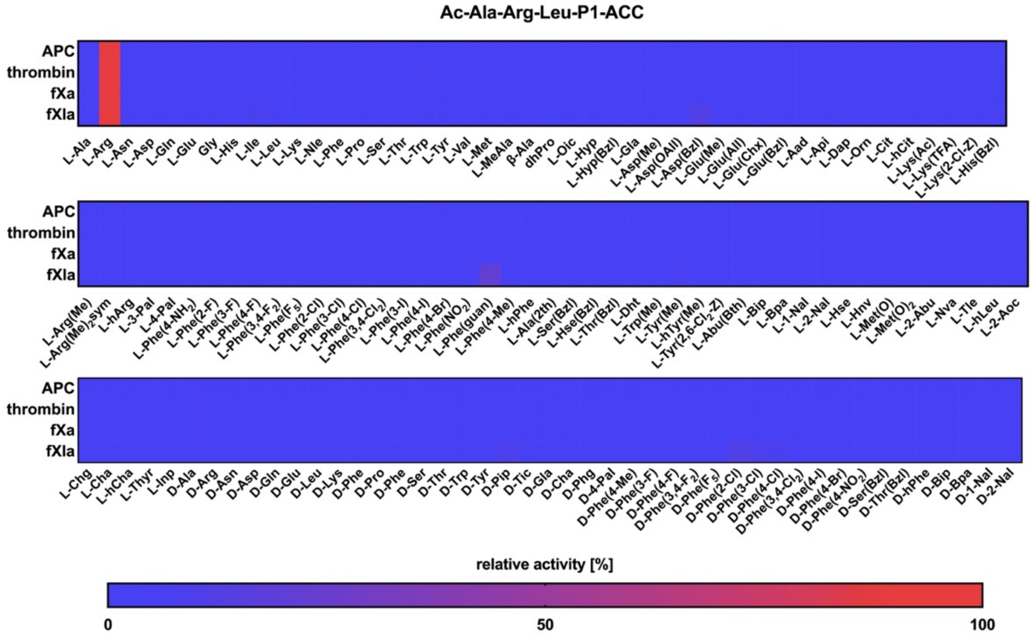

Figure S1. Substrate specificity profiles for APC, thrombin, fXa, and fXIa at the P1 position presented as heatmaps [1].

To determine the binding pocket preferences at the P4-P2 positions we utilized the Hybrid Combinatorial Substrate Library (HyCoSuL) approach. Since fXIa preferentially accommodated arginine in the S1 pocket, we employed the combinatorial library with arginine fixed at the P1 position. This library consisted of three tetrapeptide sublibraries (Ac-P4-Mix-Mix-Arg-ACC, Ac-Mix-P3-Mix-Arg-ACC, Ac-Mix-Mix-P2-Arg-ACC) and contained a natural and a large pool of unnatural amino acids at the investigated position (P4, P3 or P2) (135 amino acids for P2, 136 amino acids for P3 and P4) and an equimolar mixture of natural amino acids (Mix) at the remaining positions (18 natural amino acids and norleucine (cysteine and methionine were not used due to the possibility of oxidation)). Each vial of each sublibrary contains 19*19 = 361 compounds, and the entire P2/P3/P4 sublibrary 19*19*135/136 = 48,735/49,096. The compounds in all three sublibraries are 19*19*135/136*3 = 146,205/147,288.

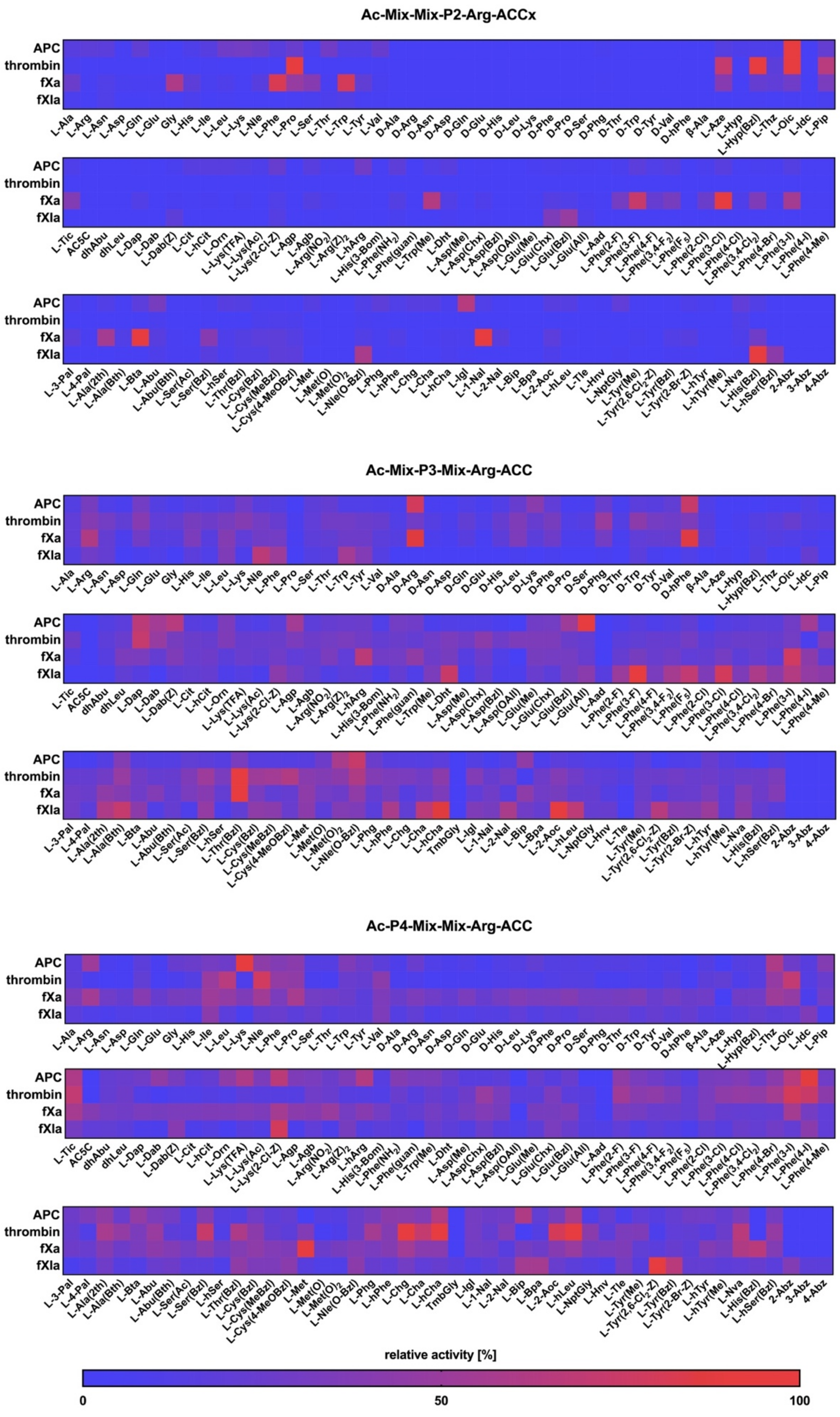

Figure S2. Substrate specificity profiles for APC, thrombin, fXa, and fXIa at the P4-P2 positions presented as heatmaps [1].

ABPs and inhibitor synthesis

The peptide sequences of the best two substrates were utilized to design irreversible ABPs (two biotinylated and one fluorescent) and one inhibitor. In the first step, 100 mg of 2-chlorotrityl resin (for each ABP) was placed in the peptide synthesis vessel and swollen in anhydrous DCM for 30 min, and then, the resin was washed once with DCM. Next, Fmoc-P2-OH (2.5 equiv.) was dissolved in anhydrous DCM, preactivated with DIPEA (3 equiv., 84 mL), added to the resin under an argon atmosphere, and stirred gently for 12 h. After that, the mixture was filtered, the resin was washed with DCM (three times), and the remaining active sites on the 2-chlorotrityl resin were deactivated with DCM/MeOH/DIPEA (%, v/v/v, 80:15:5) solution for 1 h. Next, the mixture was filtered, the resin was washed with DMF (six times), and N-terminal Fmoc-protecting group deprotection was performed using 20% piperidine in DMF (5, 5, and 25 min). After Fmoc-protecting group removal, the P3, P4 amino acids, and Fmoc-PEG(4)-OH were attached to the H_2N-P2-resin with HOBt (2.5 equiv., 60 mg) and DICI (2.5 equiv., 52 mL) as coupling reagents in coupling/deprotection cycles. The biotin tag was coupled to H_2N-PEG(4)-P4-P3-P2-resin using HBTU (2.5 equiv., 152 mg) and DIPEA (2.5 equiv., 70 mL) as coupling reagents in a DMF/DMSO mixture (%, v/v, 50:50). After 3 h, the resin was washed with DMF (six times), DCM (three times), and MeOH (three times) and dried over P_2O_5. The crude peptide was cleaved from the resin with a mixture of DCM/TFE/AcOH (%, v/v/v, 80:10:10). The solution was filtered, concentrated, and lyophilized. Next, the reactive warhead diphenyl phosphonate was synthesized according to a previously described methodology, in which a phosphonate analog of ornithine undergoes a guanidinylation reaction to obtain the final reaction product – (Cbz-Arg$(Boc)_2^P(OPh)_2$) [2]. The Cbz-protecting group was removed using hydrogen and palladium on carbon. The obtained H_2N-Arg$(Boc)_2^P(OPh)_2$ (1 equiv.) was coupled with a peptide sequence biotin-PEG(4)-P4-P3-P2-COOH (1.2 equiv.) with HATU (1.2 equiv.) and 2,4,6-collidine (4 equiv.). The reaction was monitored with analytical HPLC, and after 2 h, the product was extracted to ethyl acetate with 5% $NaHCO_3$, 5% citric acid, and brine. The organic phase was collected, dried over $MgSO_4$, and evaporated. Finally, the side chain amino acid protecting groups were removed with a mixture of TFA/DCM/TIPS (%, v/v/v, 80:15:5). After 30 min, solvents were removed with argon flow, and the obtained product (biotin-PEG(4)-P4-P3-P2-Arg$^P(OPh)_2$) was dissolved in peptide grade DMSO, purified on HPLC, and lyophilized. The ABP purity was confirmed by analytical HPLC and analyzed using HRMS. Additionally, NMR analysis was performed. ABP was then dissolved in peptide grade DMSO to a final concentration of 10 mM and stored at -80 °C until use.

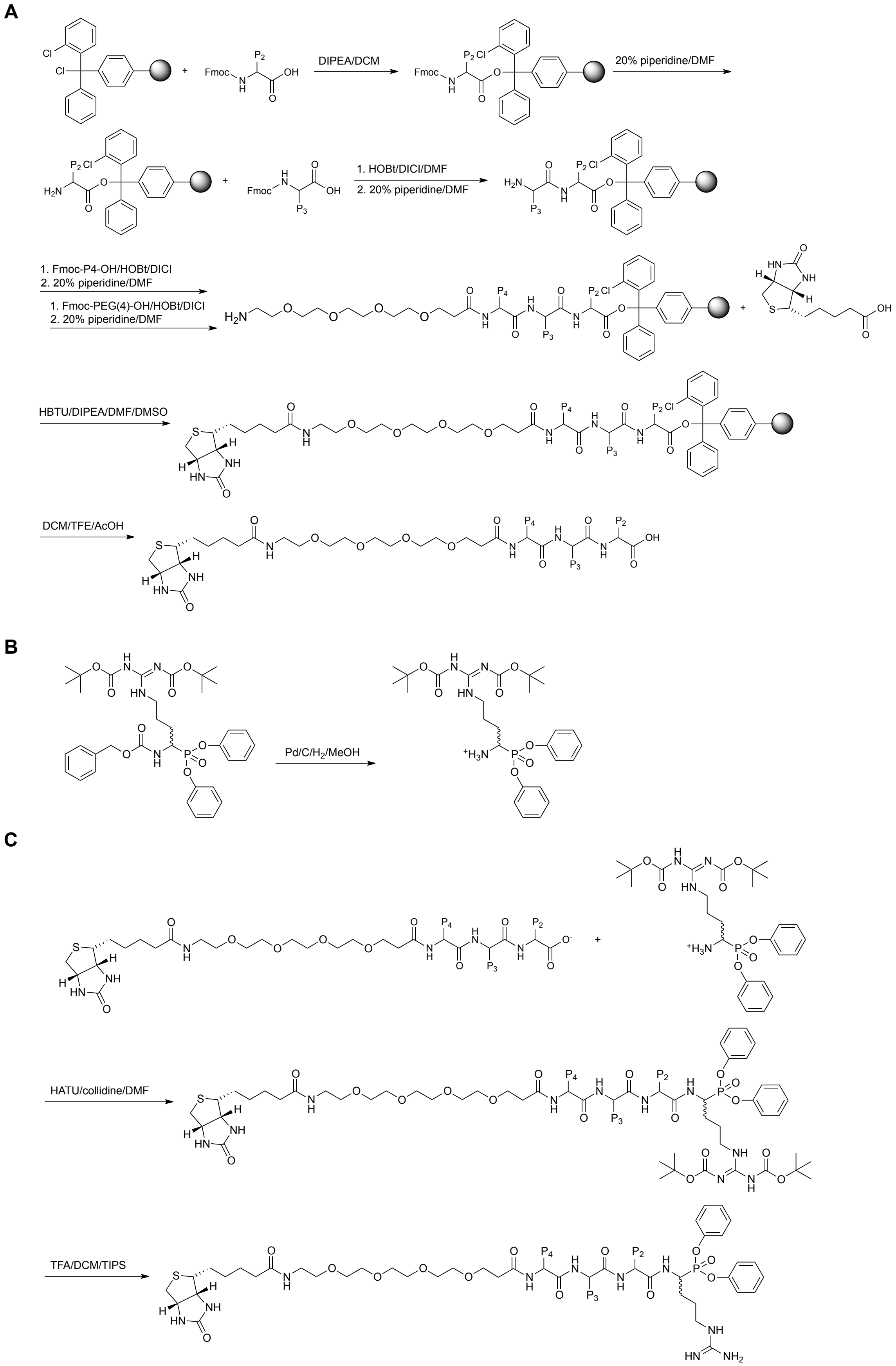

Scheme S1. Synthesis of biotinylated ABPs. (**A**) Synthesis of peptide fragments containing linker and biotin. (**B**) Deprotection of the reactive warhead. (**C**) Coupling of peptide fragments with the reactive warhead.

In the case fluorescently labeled probe, to the amine group of H_2N-PEG(4)-P4-P3-P2-resin, the BODIPY FL fluorophore was coupled. The amount of 2-chlorotrityl resin was reduced to 50 mg. BODIPY FL (0.3 equiv., 9 mg) was dissolved in DMF, preactivated with DIPEA (1.2 equiv., 17 mL), and stirred with the resin overnight. Next, the resin was washed with DMF (six times), DCM (three times), and MeOH (three times) and dried over P_2O_5. The remaining steps were analogous to the synthesis of the biotin-labeled ABPs.

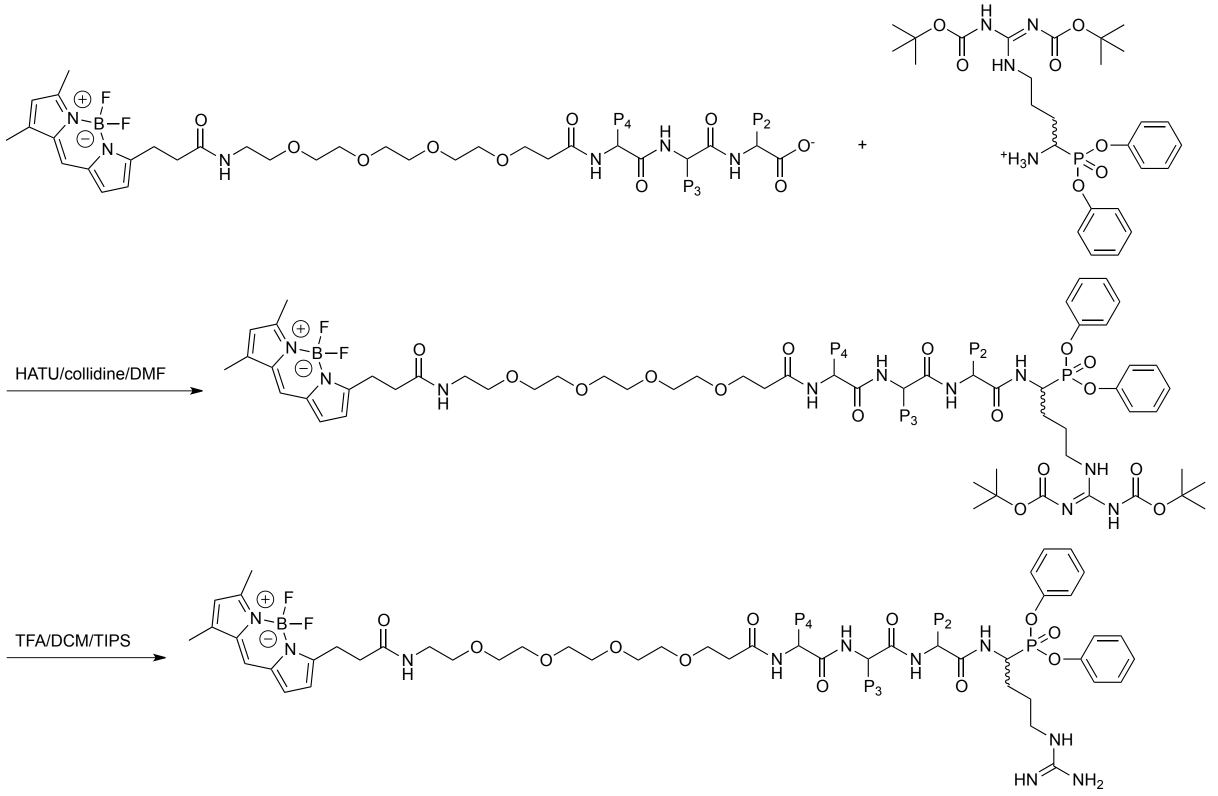

Scheme S2. Synthesis of the fluorescently labeled ABP.

To obtain the inhibitor, the amino group of H_2N-P4-P3-P2-resin was acetylated using 5 equiv. of AcOH, 5 equiv. of HBTU, and 5 equiv. of DIPEA in DMF. After 1 h, the solution was filtered, and the resin was washed with DMF (six times), DCM (three times), and MeOH (three times) and dried over P_2O_5. The remaining steps were analogous to the synthesis of the biotinylated ABPs.

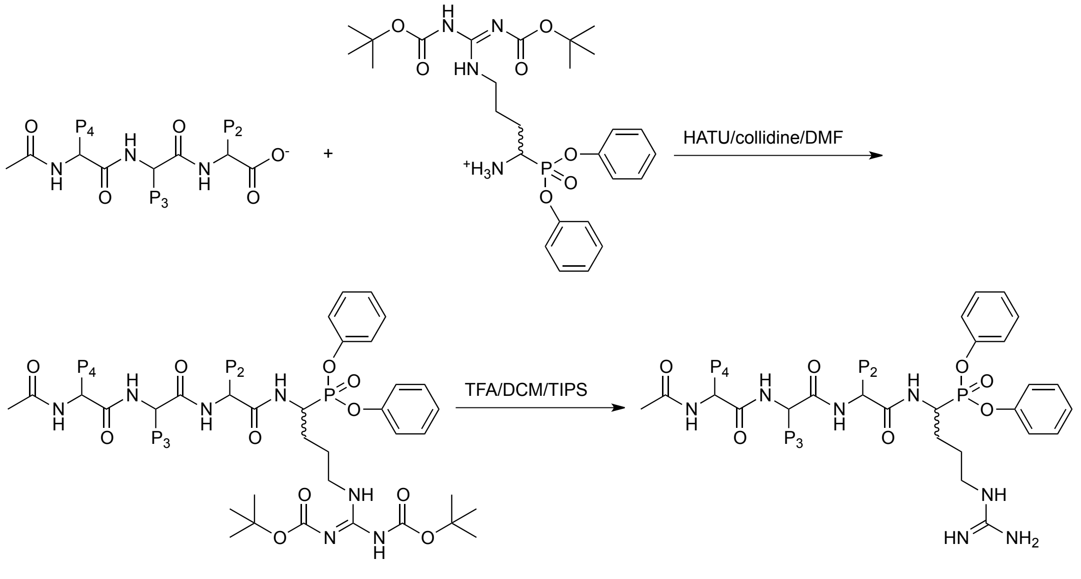

Scheme S3. Synthesis of the inhibitor.

Purity and MS analysis of fXIa compounds

Table S1. Purity and MS analysis of fXIa compounds.

	Structure	$[M+H]^{+/2+}$ calculated	$[M+H]^{+/2+}$ measured	Purity
**SMXI1**	Ac-Tyr(2,6-Cl_2-Z)-Nle-His(Bzl)-Arg-ACC	1078.4109	1078.4105	≥ 99%
**SMXI2**	Ac-Tyr(2,6-Cl_2-Z)-Phe(3-F)-His(Bzl)-Arg-ACC	1130.3859	1130.3857	≥ 99%
**SMXI3**	Ac-Tyr(2,6-Cl_2-Z)-2-Aoc-His(Bzl)-Arg-ACC	1106.4423	1106.4424	≥ 99%
**SMXI4**	Ac-Tyr(2,6-Cl_2-Z)-Tyr(2,6-Cl_2-Z)-His(Bzl)-Arg-ACC	644.6829	644.6832	≥ 99%
**SMXI5**	Ac-Tyr(2,6-Cl_2-Z)-Nle-Glu(Bzl)-Arg-ACC	1070.3945	1070.3940	≥ 99%
**SMXI6**	Ac-Tyr(2,6-Cl_2-Z)-Phe(3-F)-Glu(Bzl)-Arg-ACC	1122.3695	1122.3702	≥ 99%
**SMXI7**	Ac-Tyr(2,6-Cl_2-Z)-2-Aoc-Glu(Bzl)-Arg-ACC	1098.4259	1098.4255	≥ 95%
**SMXI8**	Ac-Tyr(2,6-Cl_2-Z)-Tyr(2,6-Cl_2-Z)-Glu(Bzl)-Arg-ACC	1280.3417	1280.3430	≥ 99%
**SMXI9**	Ac-Tyr(Bzl)-Nle-His(Bzl)-Arg-ACC	1010.4888	1010.4877	≥ 99%
**SMXI10**	Ac-Tyr(Bzl)-Phe(3-F)-His(Bzl)-Arg-ACC	1062.4637	1062.4641	≥ 99%
**SMXI11**	Ac-Tyr(Bzl)-2-Aoc-His(Bzl)-Arg-ACC	1038.5201	1038.5369	≥ 99%
**SMXI12**	Ac-Tyr(Bzl)-Tyr(2,6-Cl_2-Z)-His(Bzl)-Arg-ACC	1218.4371	1218.4377	≥ 95%
**SMXI13**	Ac-Tyr(Bzl)-Nle-Glu(Bzl)-Arg-ACC	1002.4725	1002.4703	≥ 99%
**SMXI14**	Ac-Tyr(Bzl)-Phe(3-F)-Glu(Bzl)-Arg-ACC	1054.4475	1054.4465	≥ 99%
**SMXI15**	Ac-Tyr(Bzl)-2-Aoc-Glu(Bzl)-Arg-ACC	1030.5038	1030.5040	≥ 99%
**SMXI16**	Ac-Tyr(Bzl)-Tyr(2,6-Cl_2-Z)-Glu(Bzl)-Arg-ACC	1210.4208	1210.4214	≥ 99%
**SMXI17**	Ac-Bpa-2-Aoc-His(Bzl)-Arg-ACC	1036.5045	1036.5033	≥ 99%
**SMXI18**	Ac-Lys(2-Cl-Z)-2-Aoc-His(Bzl)-Arg-ACC	1081.5027	1081.5011	≥ 99%
**SMXI19**	Ac-Tyr(2,6-Cl_2-Z)-Dht-His(Bzl)-Arg-ACC	1153.4218	1153.4213	≥ 99%
**SMXI20**	Ac-Tyr(2,6-Cl_2-Z)-Cha-His(Bzl)-Arg-ACC	1118.4423	1118.4417	≥ 99%
**SMXI21**	Ac-Tyr(2,6-Cl_2-Z)-hCha-His(Bzl)-Arg-ACC	1132.4579	1132.4569	≥ 99%
**SMXI22**	Ac-Tyr(2,6-Cl_2-Z)-2-Aoc-Nle(O-Bzl)-Arg-ACC	1098.4623	1098.4618	≥ 97%
**SMXI23**	Ac-Tyr(2,6-Cl_2-Z)-2-Aoc-hSer(Bzl)-Arg-ACC	1070.4310	1070.4296	≥ 99%
**SMXI24**	Ac-Ile-Nle-Asn-Arg-ACC	757.3997	757.3992	≥ 99%
**P-SMXI51**	biotin-PEG(4)-Tyr(2,6-Cl_2-Z)-Nle-Glu(Bzl)-$Arg^P(OPh)_2$	1489.5841	1489.5854	≥ 95%
**P-SMXI191**	biotin-PEG(4)-Tyr(2,6-Cl_2-Z)-Dht-His(Bzl)-$Arg^P(OPh)_2$	786.8096	786.8090	≥ 97%
**P-SMXI52**	BODIPY-PEG(4)-Tyr(2,6-Cl_2-Z)-Nle-Glu(Bzl)-$Arg^P(OPh)_2$	1537.6167	1537.6165	≥ 95%
**I-SMXI5**	Ac-Tyr(2,6-Cl_2-Z)-Nle-Glu(Bzl)-$Arg^P(OPh)_2$	1058.3751	1058.3749	≥ 97%

SMXI1, Ac-Tyr(2,6-Cl_2-Z)-Nle-His(Bzl)-Arg-ACC

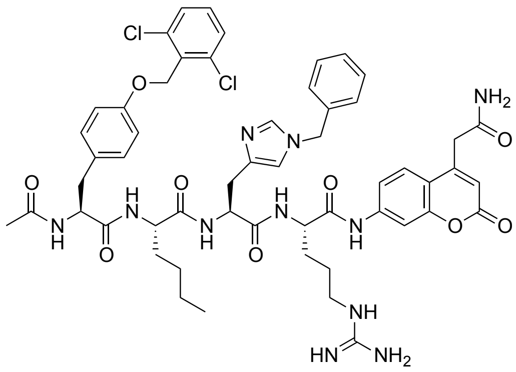

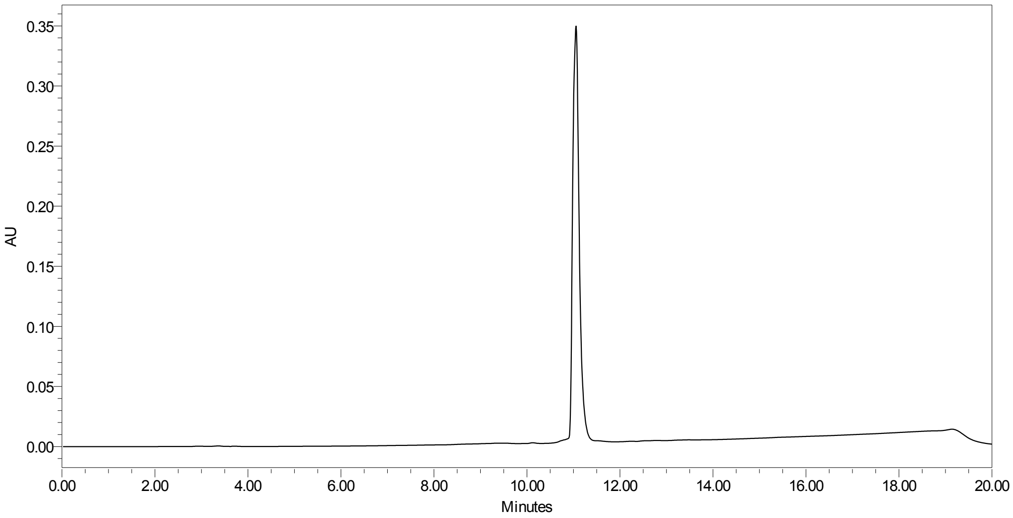

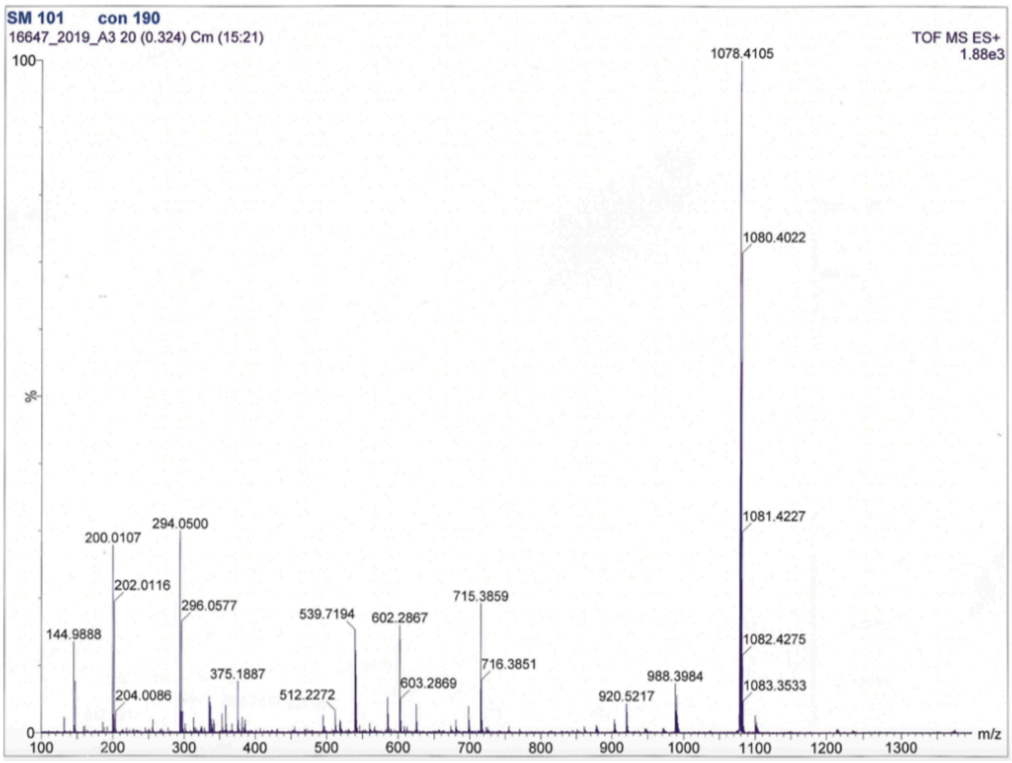

SMXI2, Ac-Tyr(2,6-Cl_2-Z)-Phe(3-F)-His(Bzl)-Arg-ACC

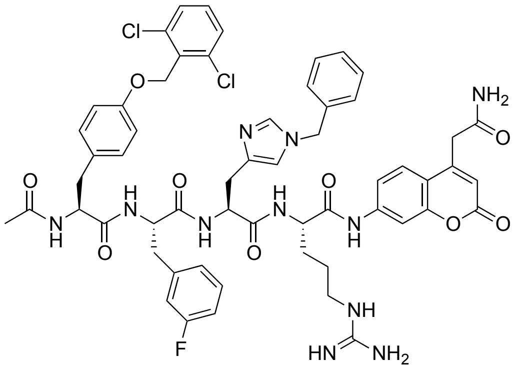

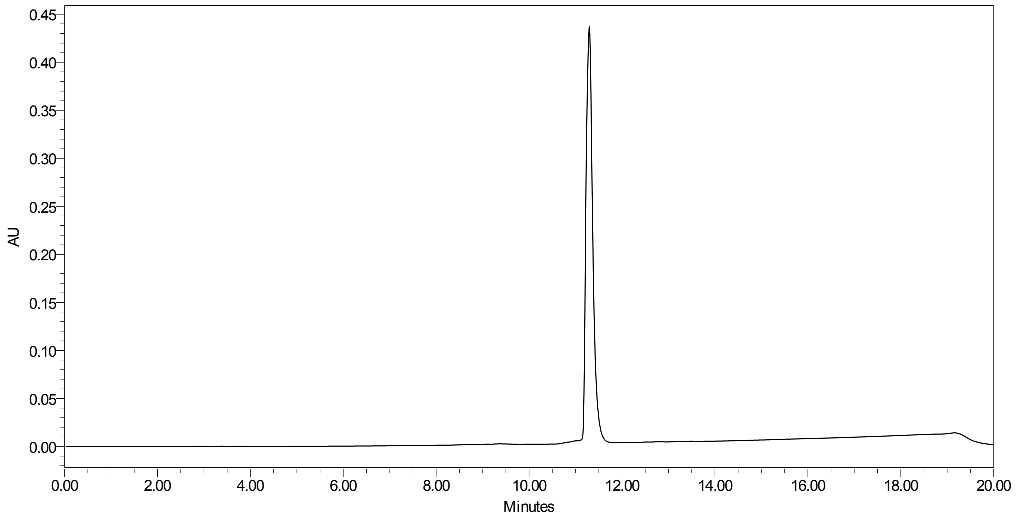

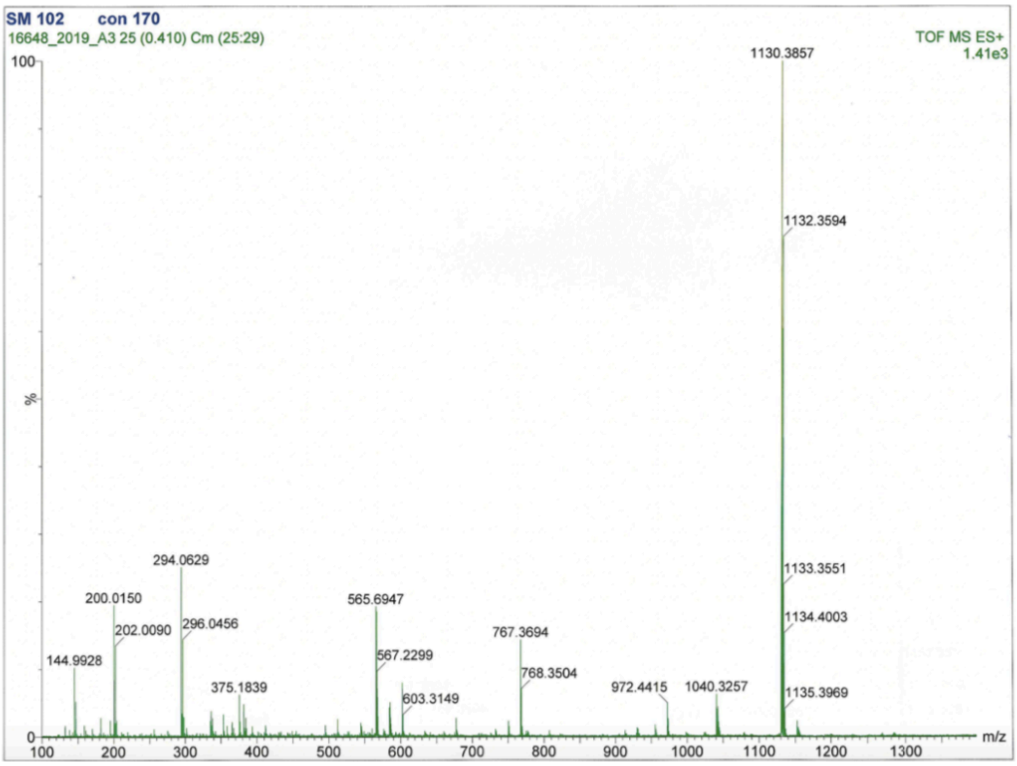

SMXI3, Ac-Tyr(2,6-Cl_2-Z)-2-Aoc-His(Bzl)-Arg-ACC

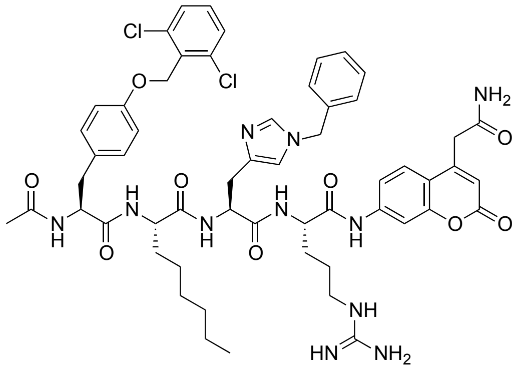

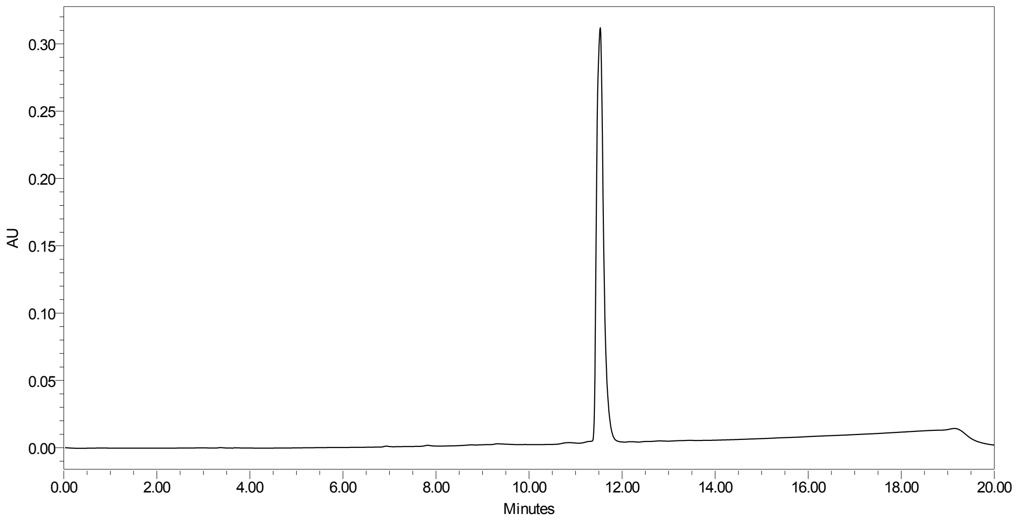

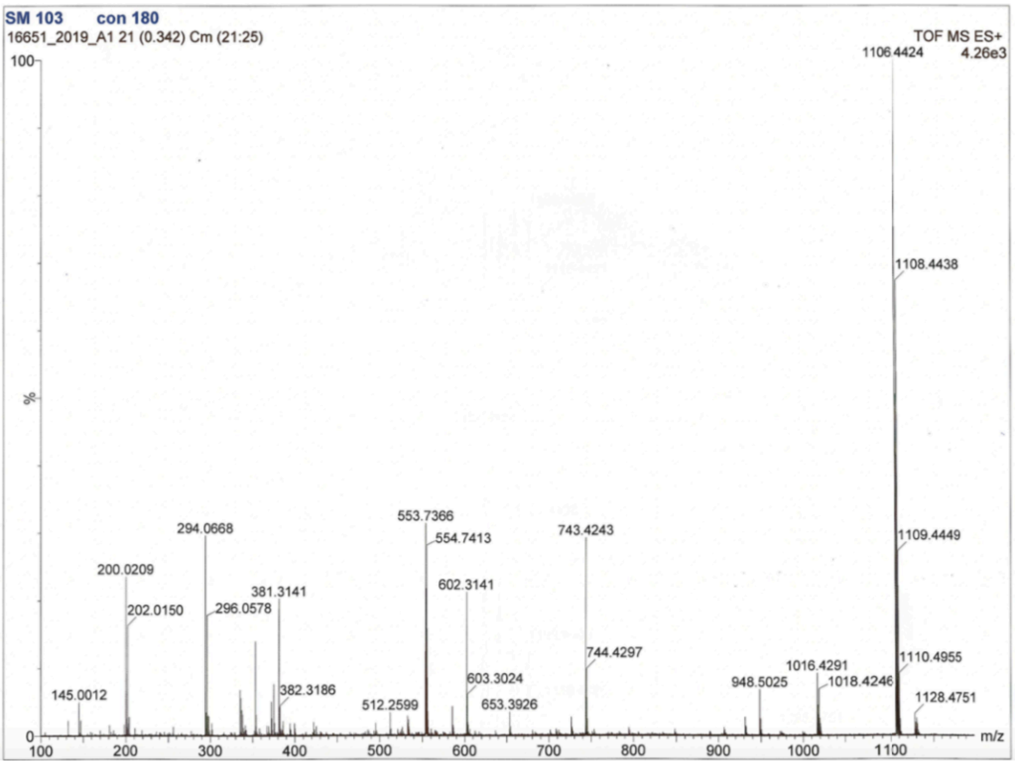

SMXI4, Ac-Tyr(2,6-Cl_2-Z)-Tyr(2,6-Cl_2-Z)-His(Bzl)-Arg-ACC

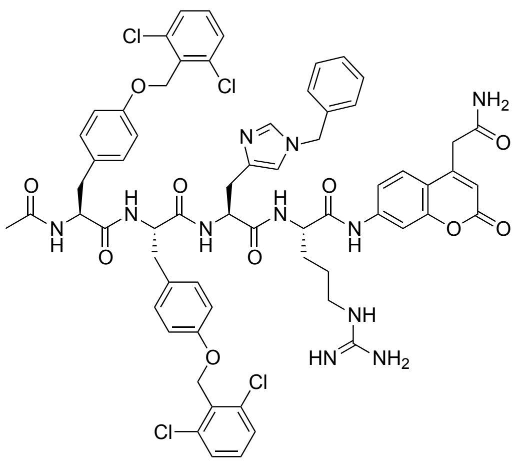

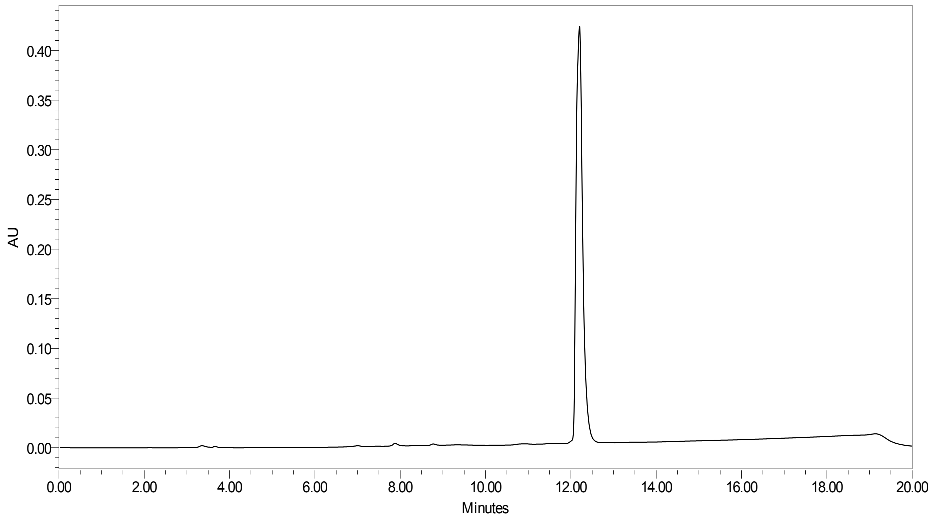

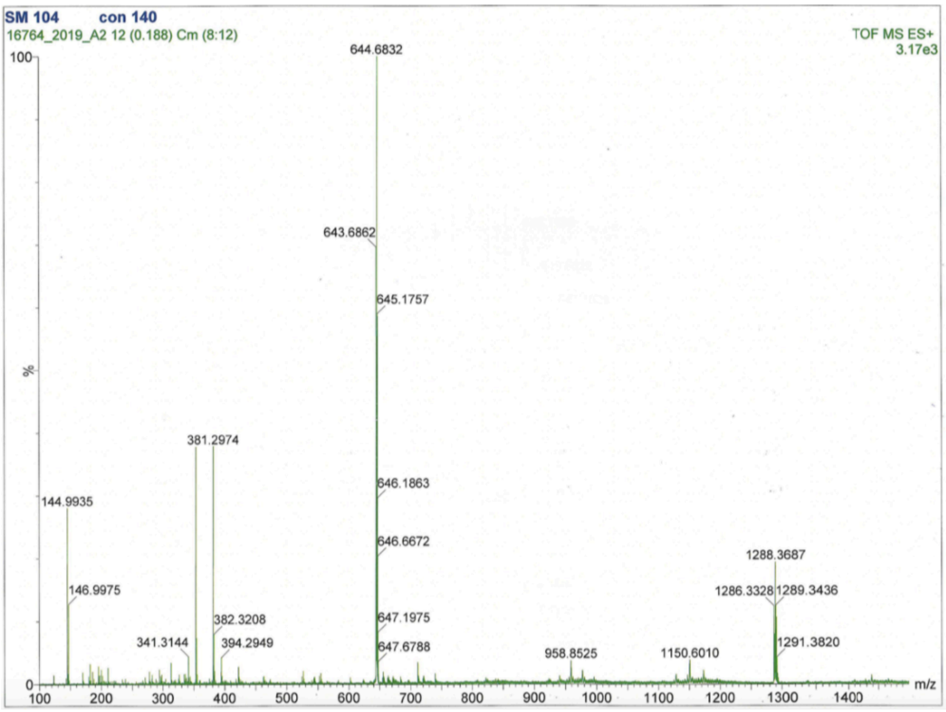

SMXI5, Ac-Tyr(2,6-Cl$_2$-Z)-Nle-Glu(Bzl)-Arg-ACC

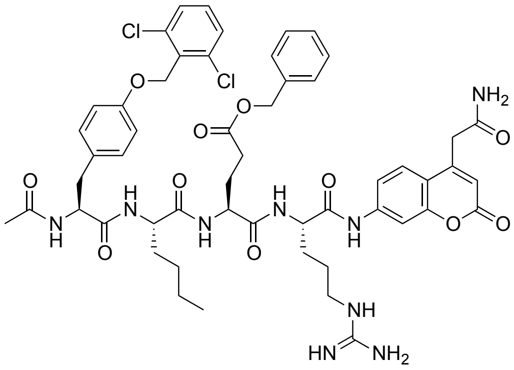

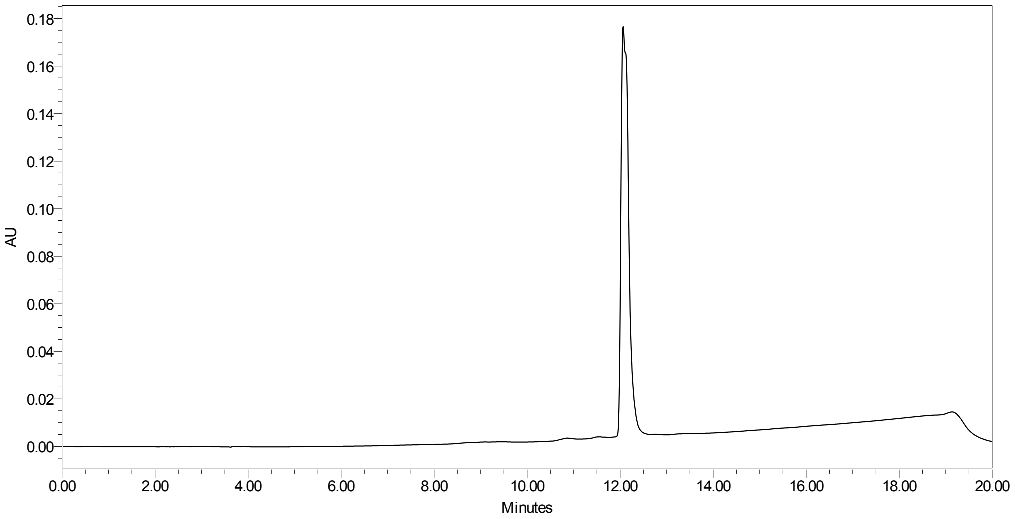

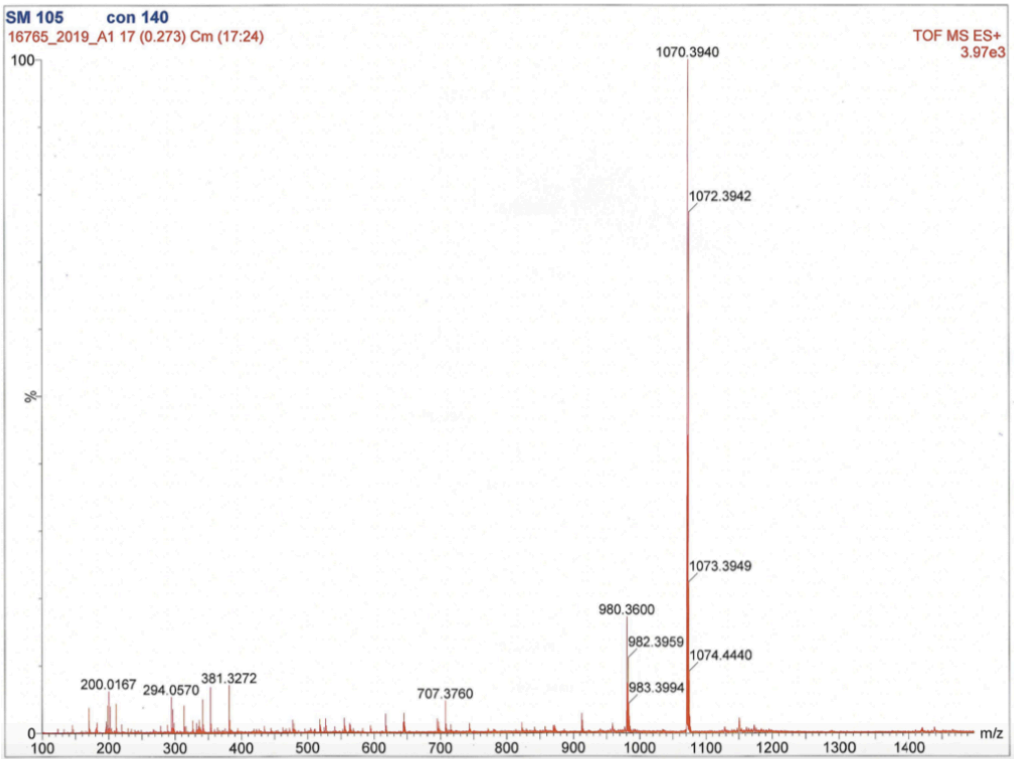

SMXI6, Ac-Tyr(2,6-Cl_2-Z)-Phe(3-F)-Glu(Bzl)-Arg-ACC

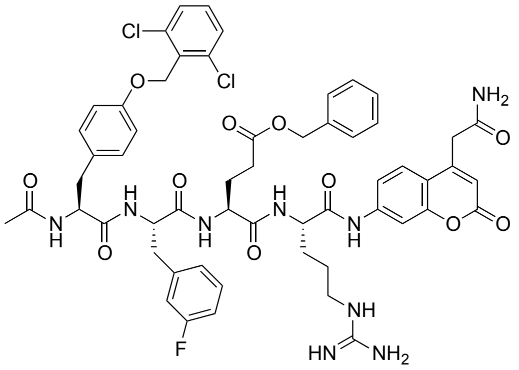

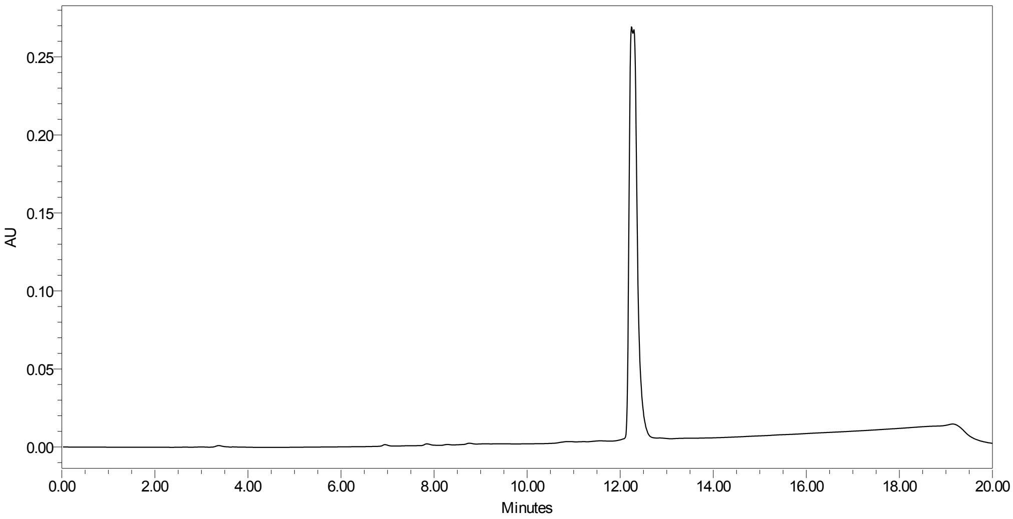

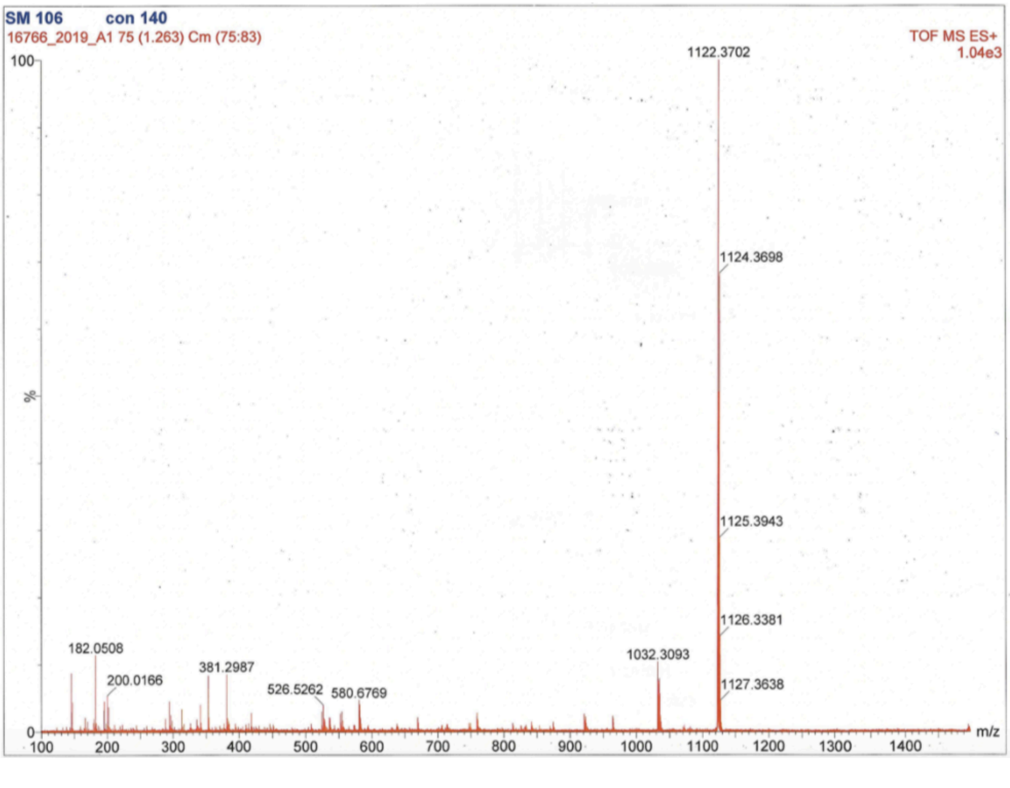

SMXI7, Ac-Tyr(2,6-Cl_2-Z)-2-Aoc-Glu(Bzl)-Arg-ACC

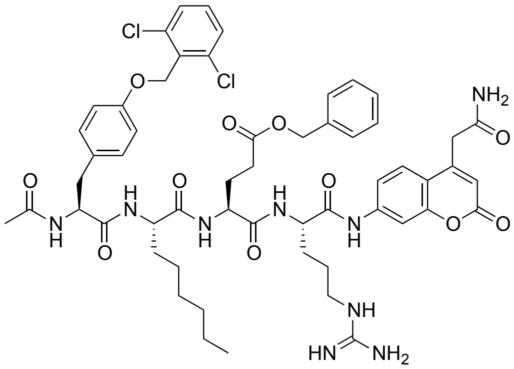

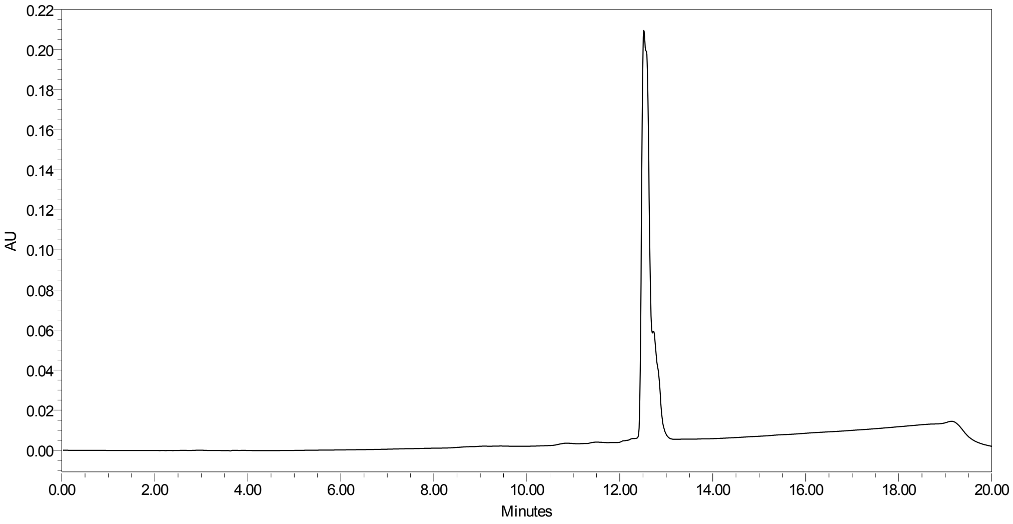

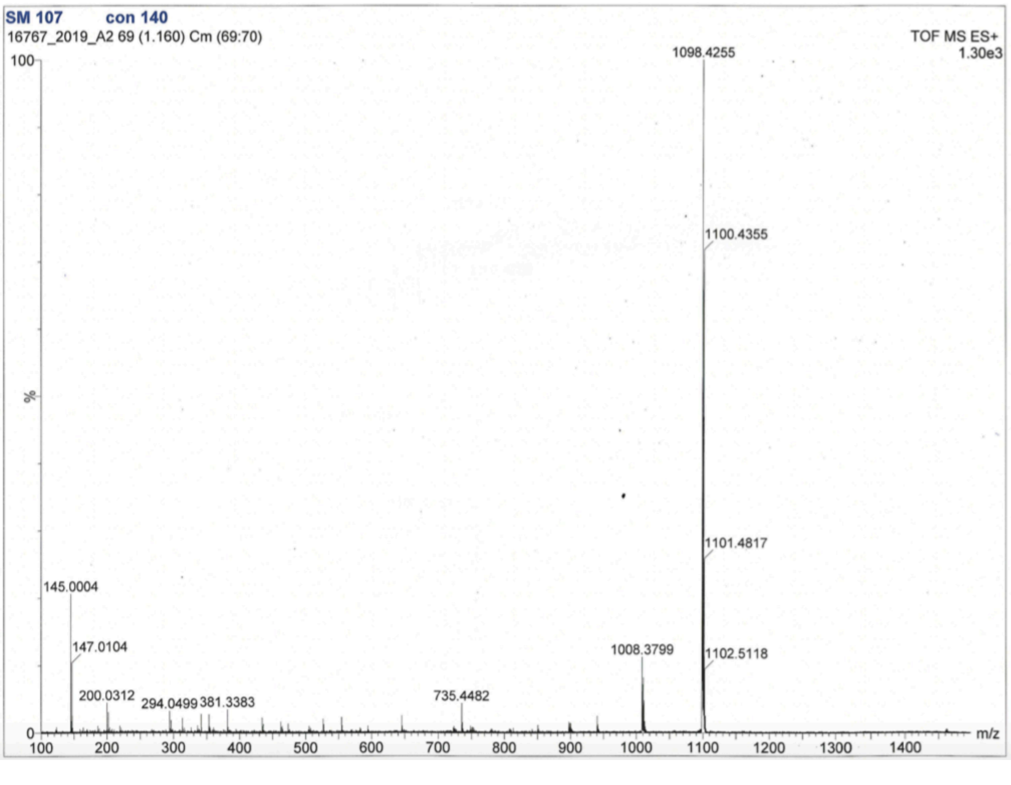

SMXI8, Ac-Tyr(2,6-Cl_2-Z)-Tyr(2,6-Cl_2-Z)-Glu(Bzl)-Arg-ACC

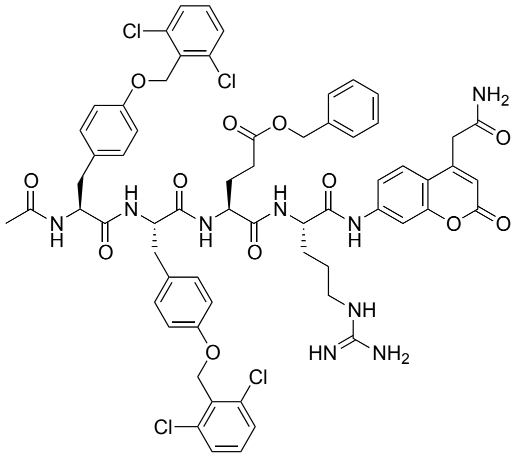

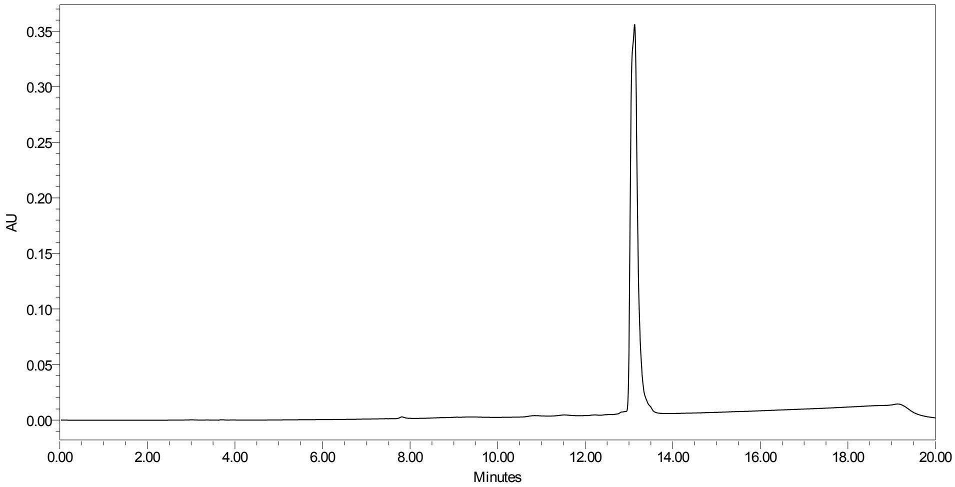

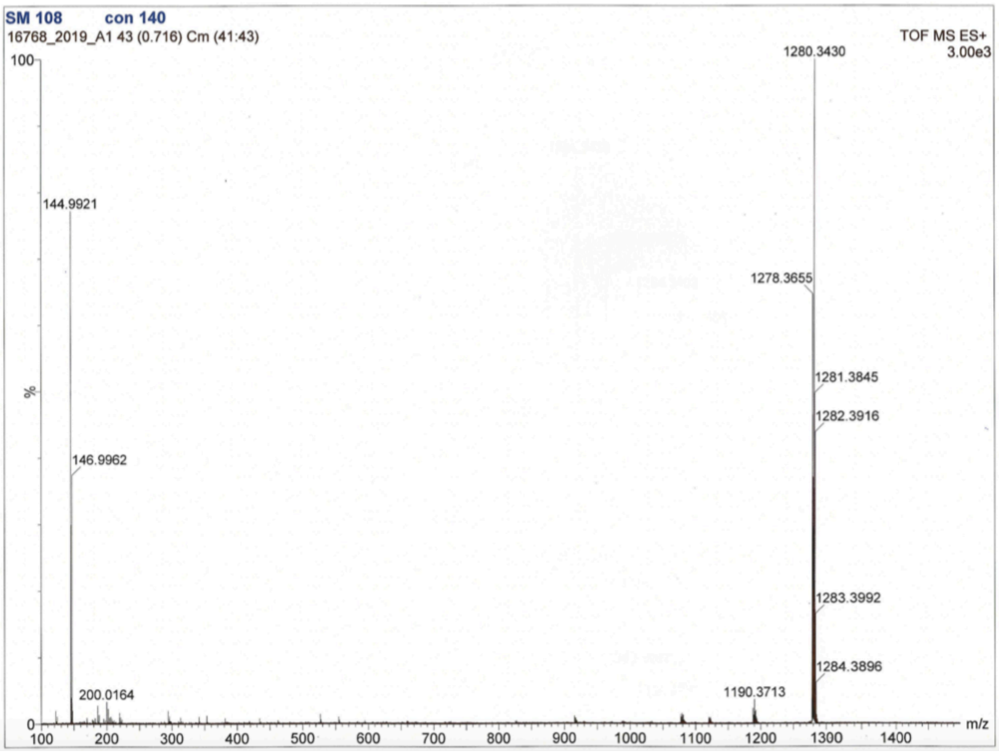

SMXI9, Ac-Tyr(Bzl)-Nle-His(Bzl)-Arg-ACC

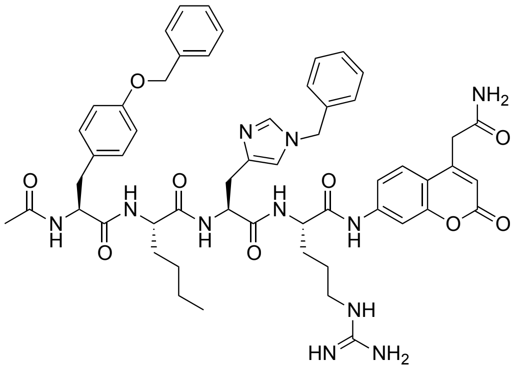

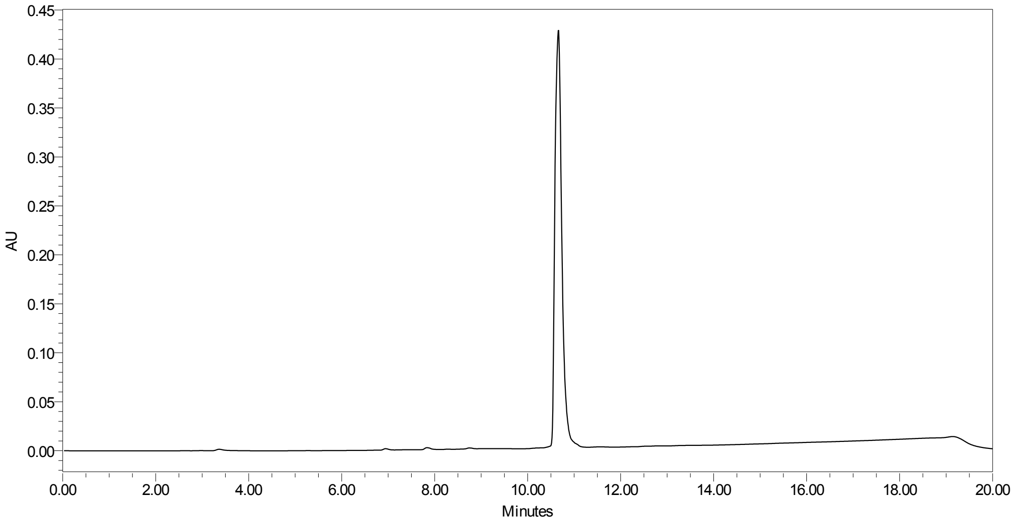

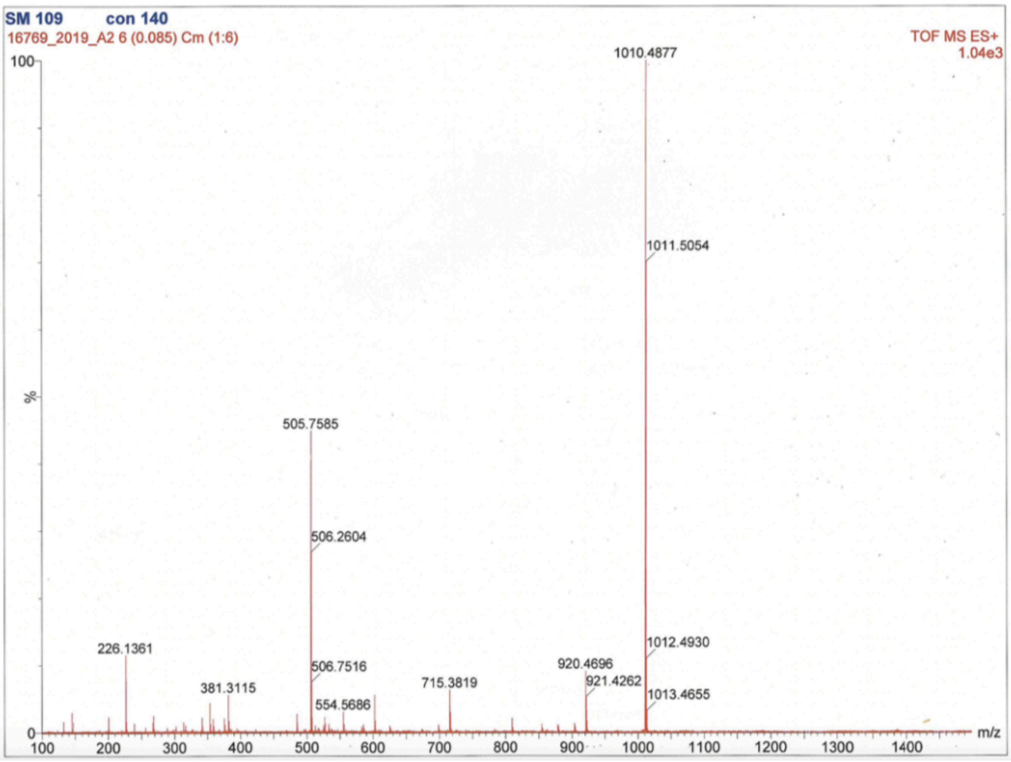

SMXI10, Ac-Tyr(Bzl)-Phe(3-F)-His(Bzl)-Arg-ACC

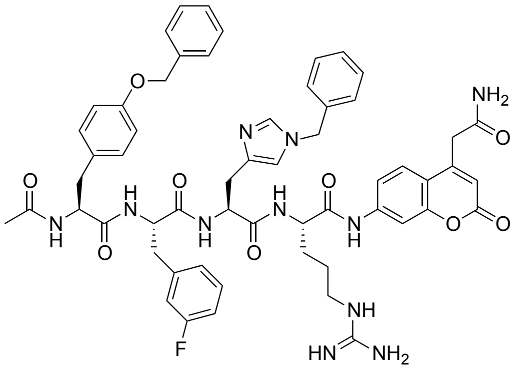

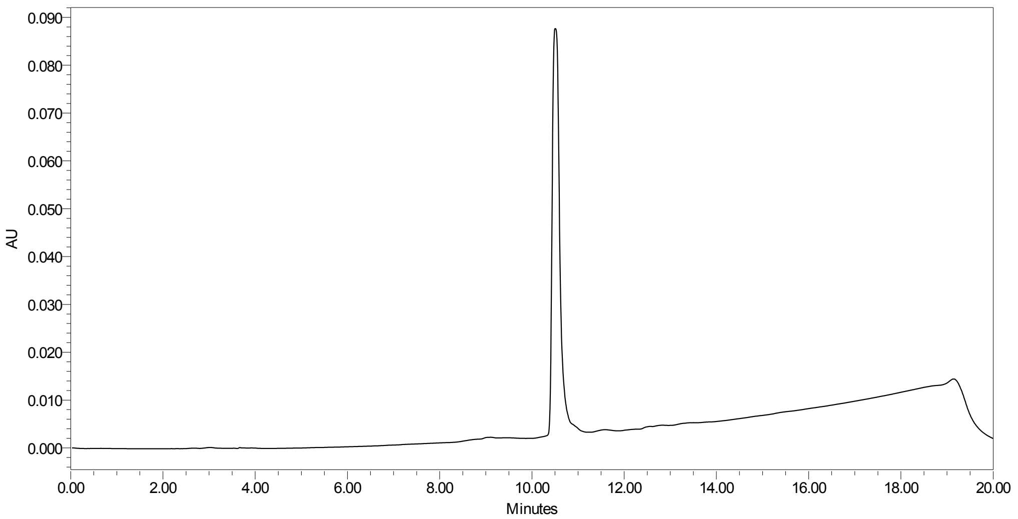

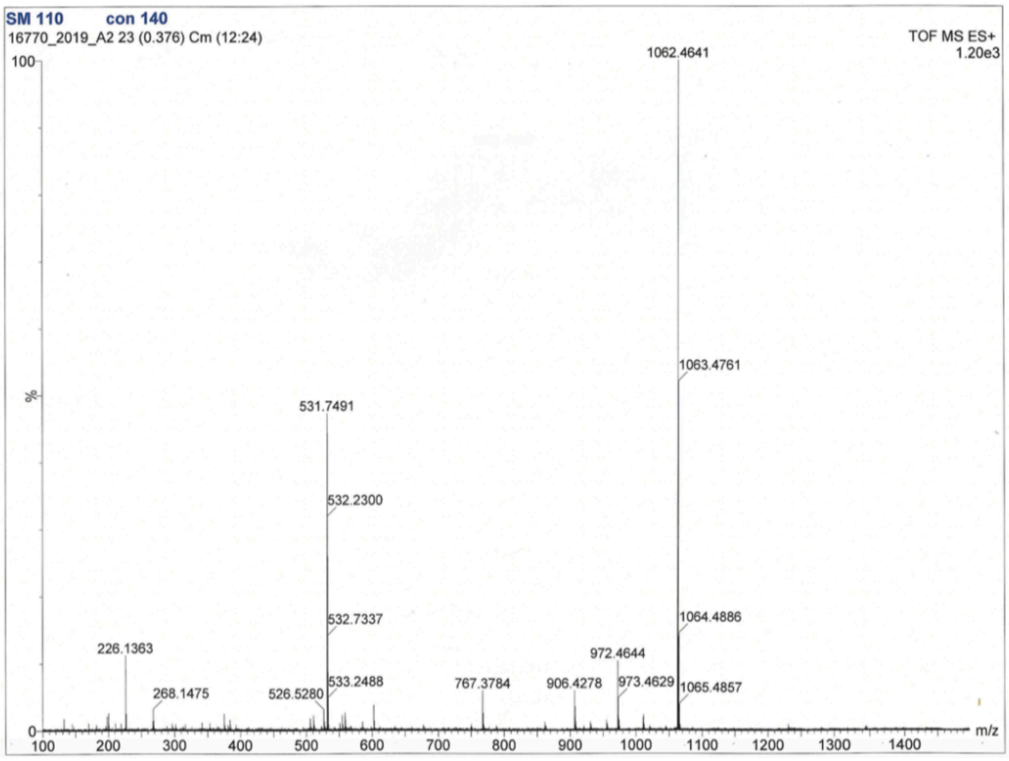

SMXI11, Ac-Tyr(Bzl)-2-Aoc-His(Bzl)-Arg-ACC

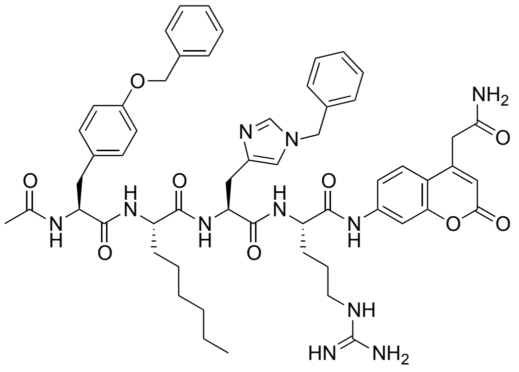

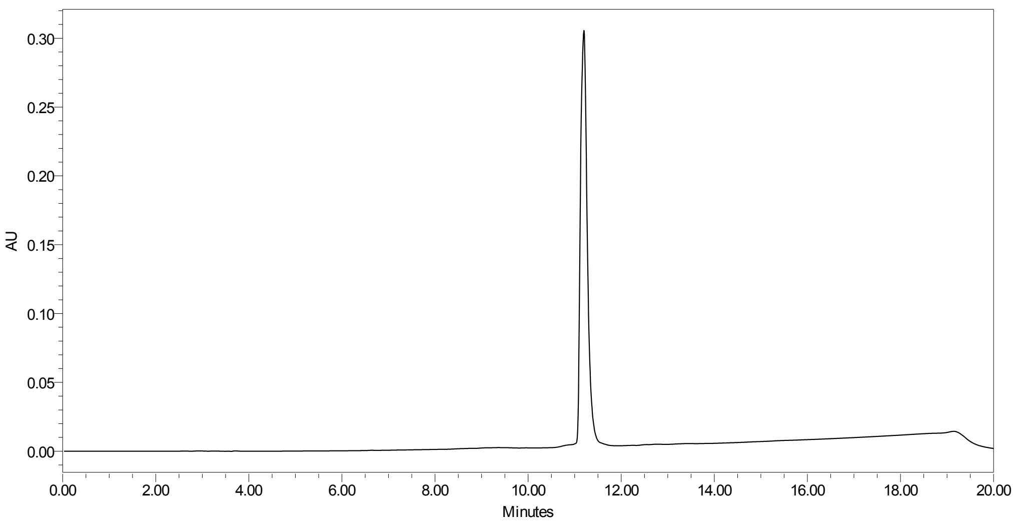

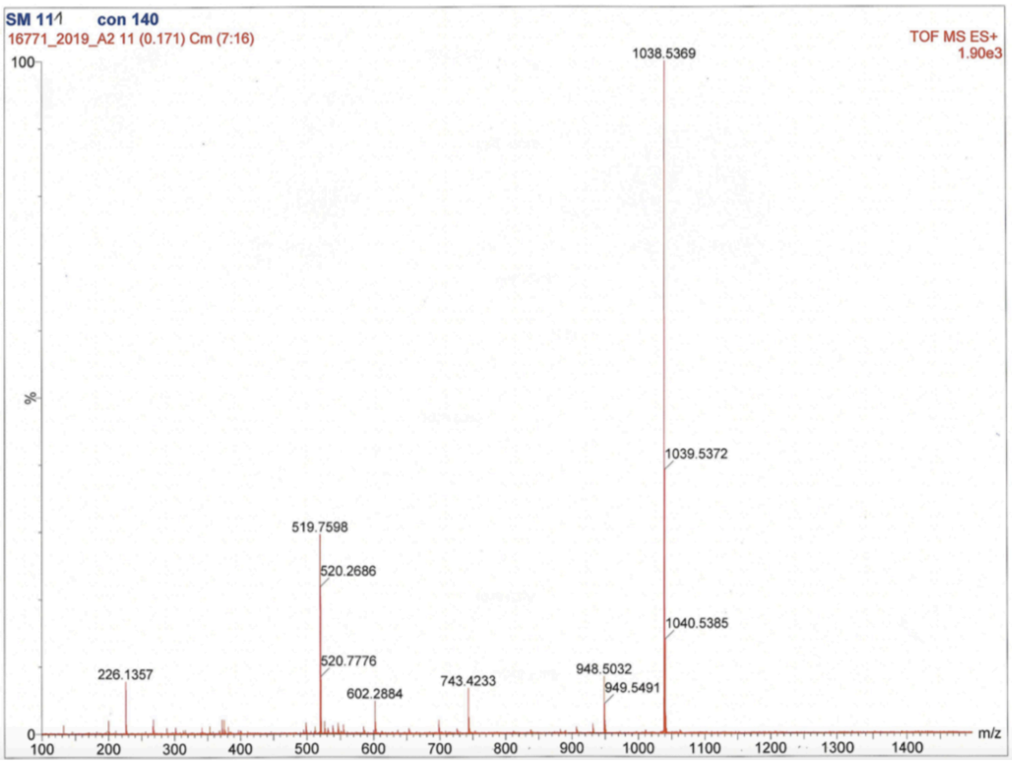

SMXI12, Ac-Tyr(Bzl)-Tyr(2,6-Cl_2-Z)-His(Bzl)-Arg-ACC

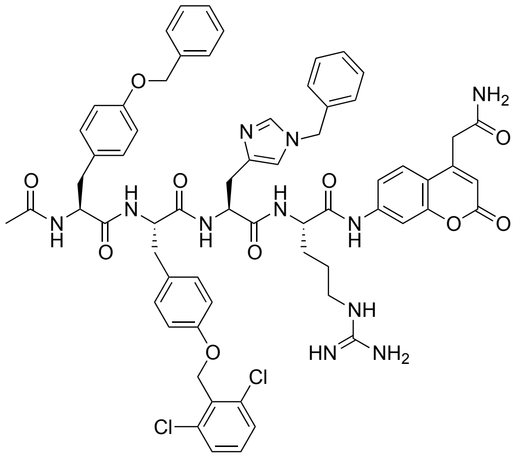

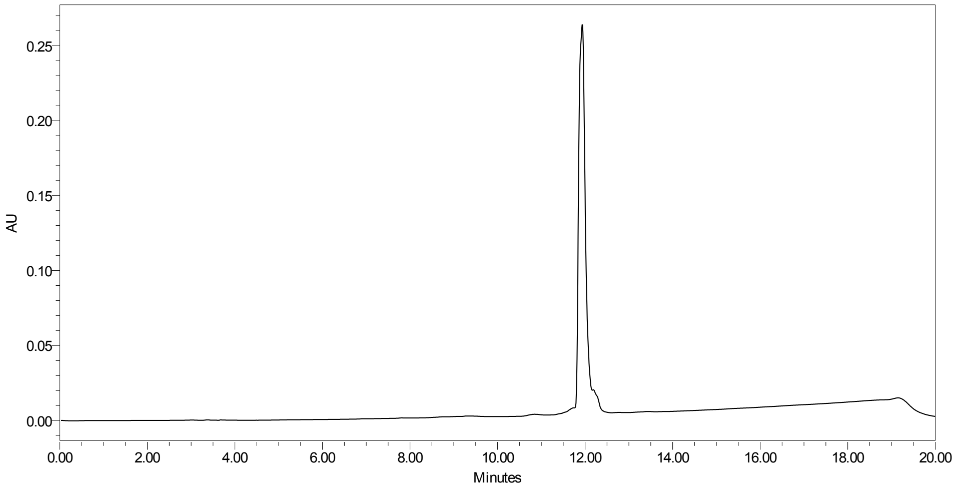

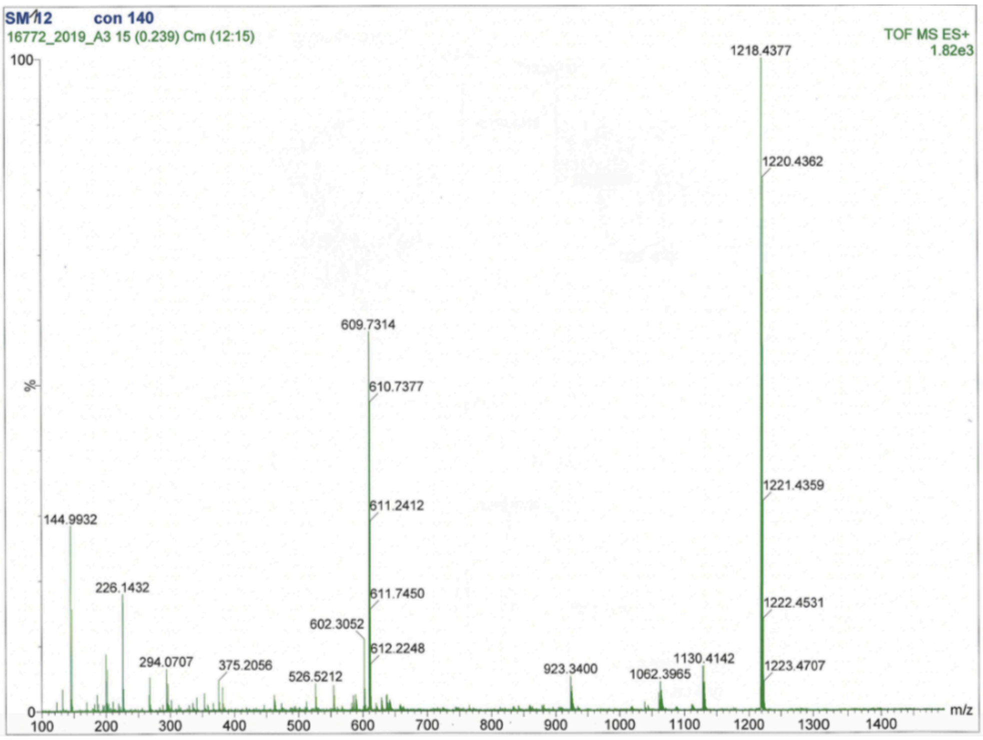

SMXI13, Ac-Tyr(Bzl)-Nle-Glu(Bzl)-Arg-ACC

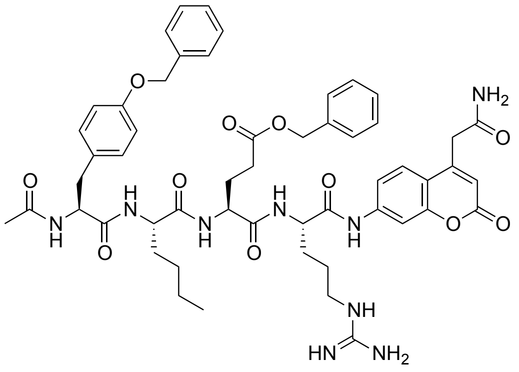

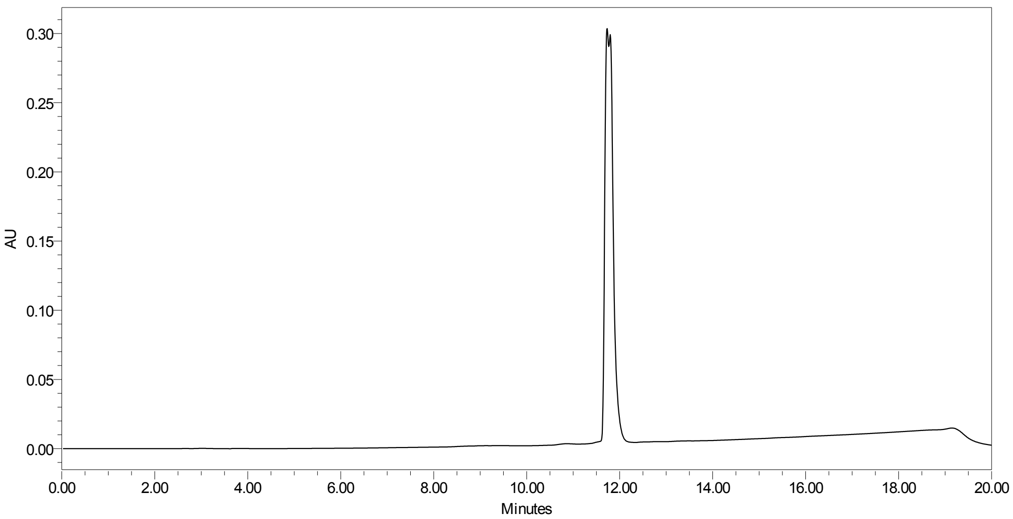

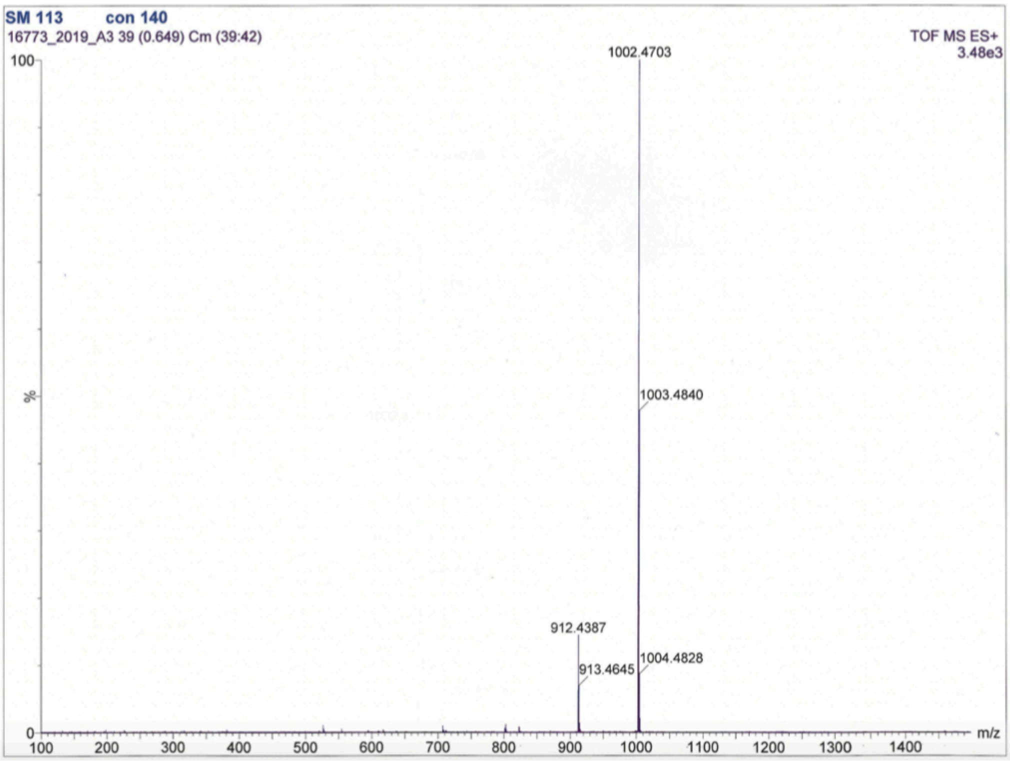

SMXI14, Ac-Tyr(Bzl)-Phe(3-F)-Glu(Bzl)-Arg-ACC

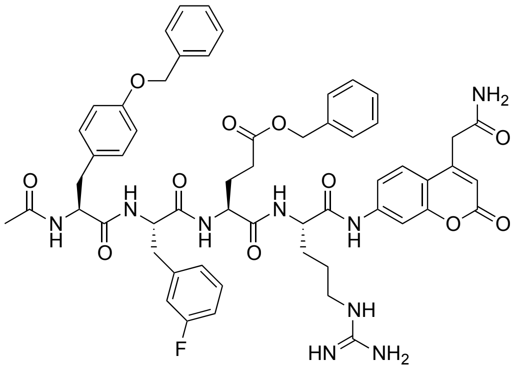

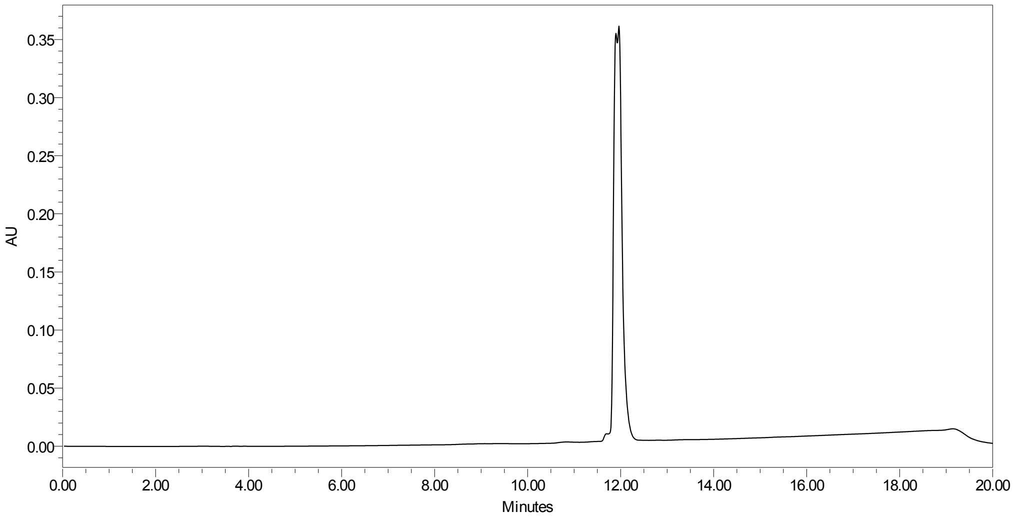

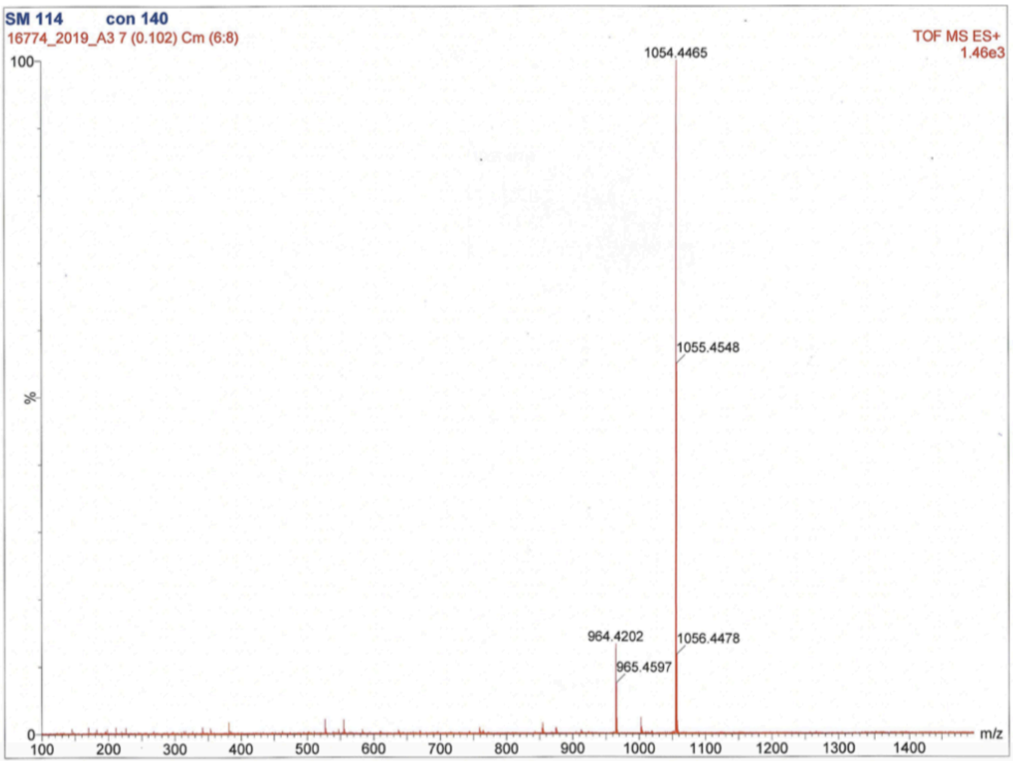

SMXI15, Ac-Tyr(Bzl)-2-Aoc-Glu(Bzl)-Arg-ACC

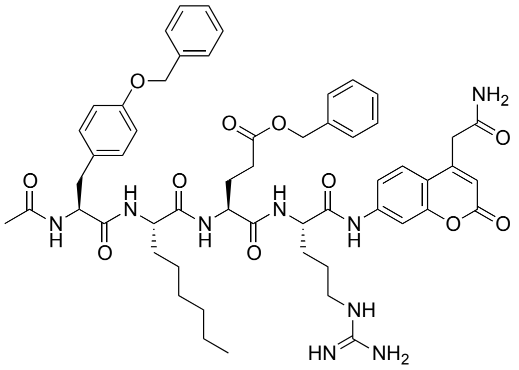

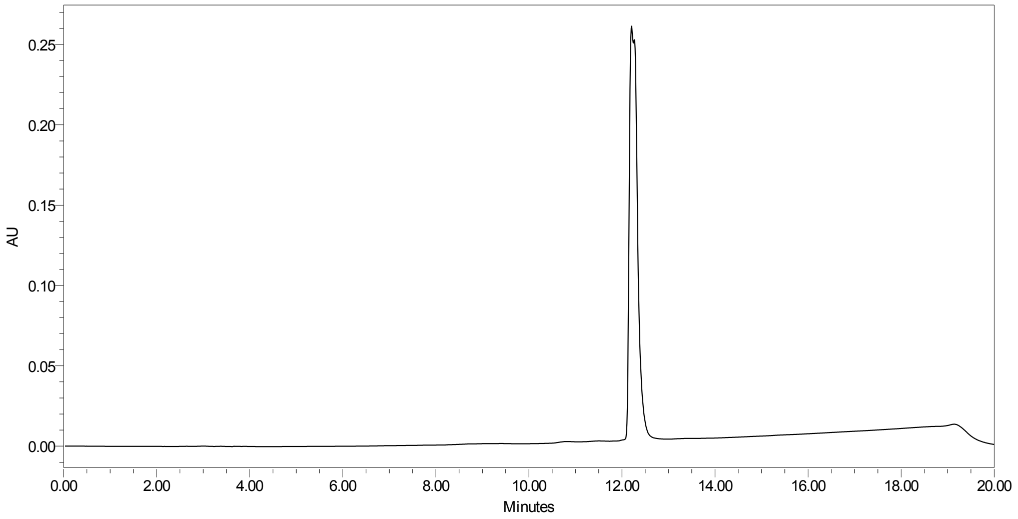

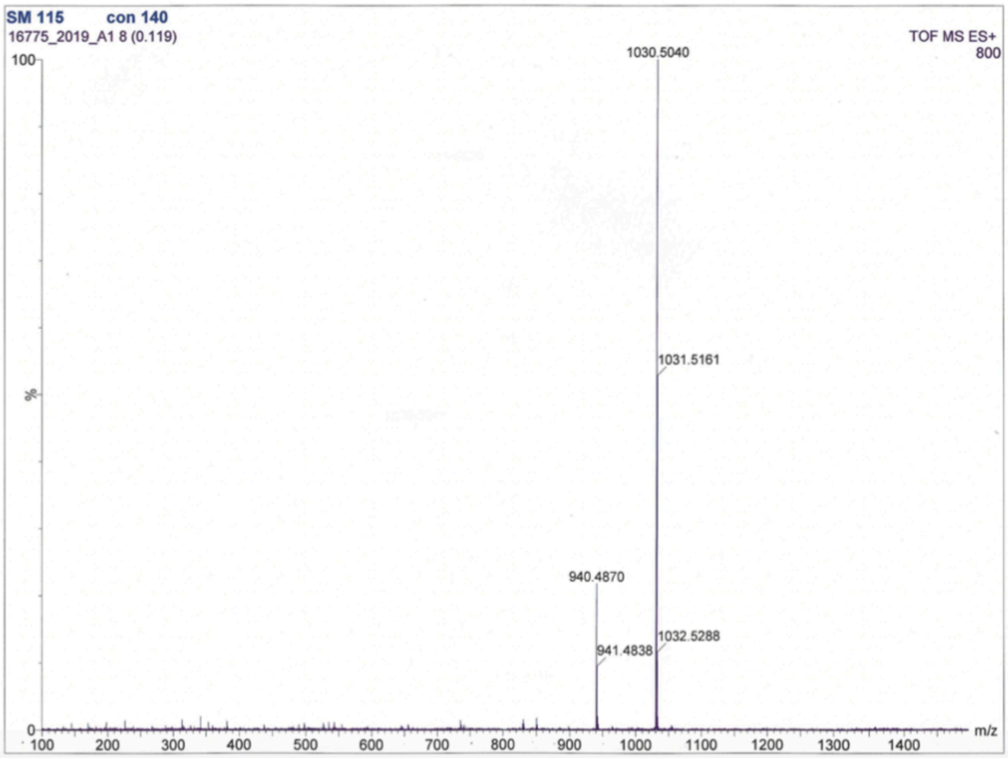

SMXI16, Ac-Tyr(Bzl)-Tyr(2,6-Cl$_2$-Z)-Glu(Bzl)-Arg-ACC

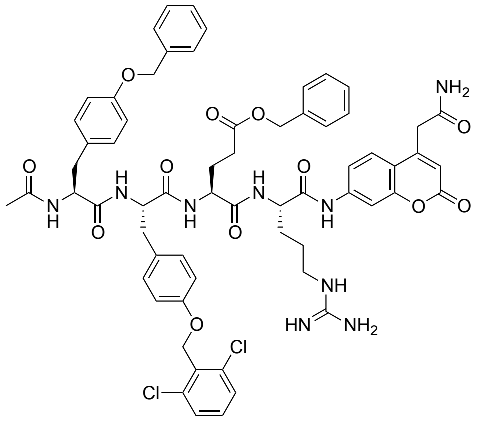

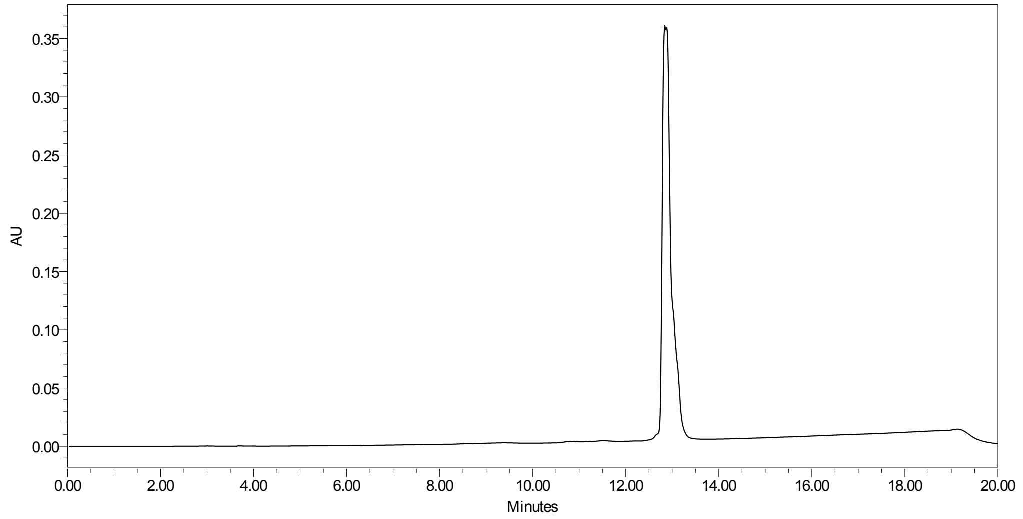

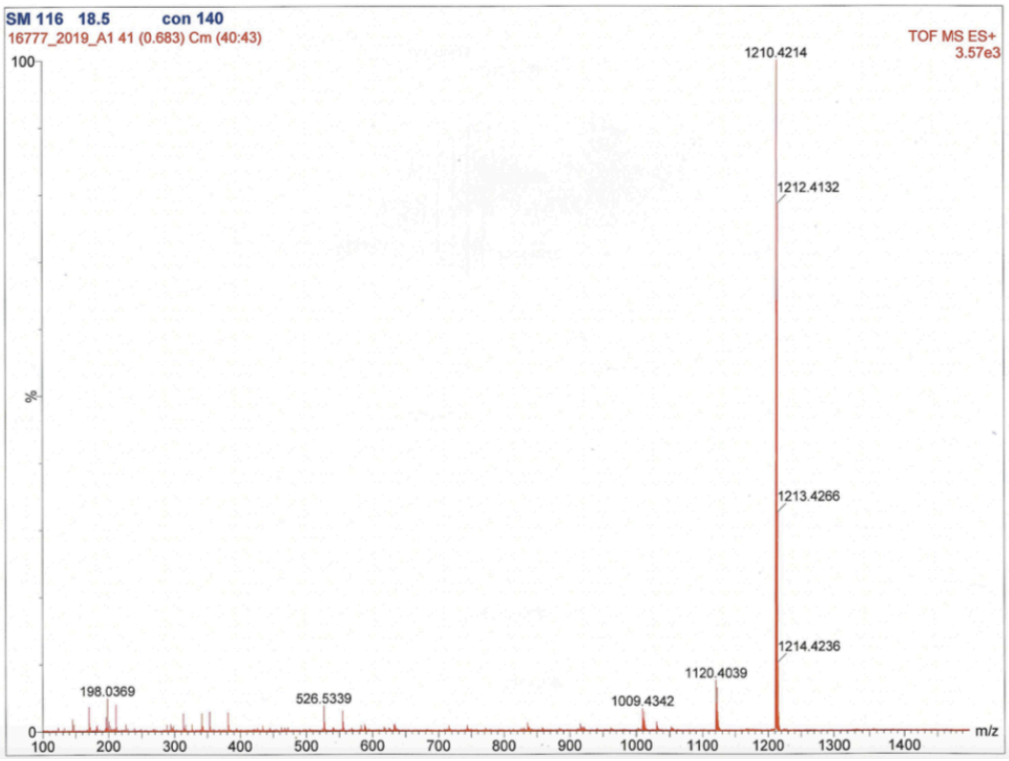

SMXI17, Ac-Bpa-2-Aoc-His(Bzl)-Arg-ACC

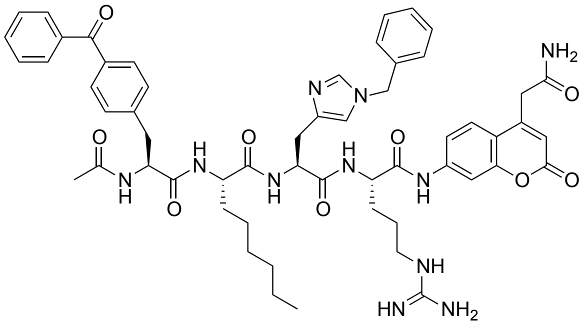

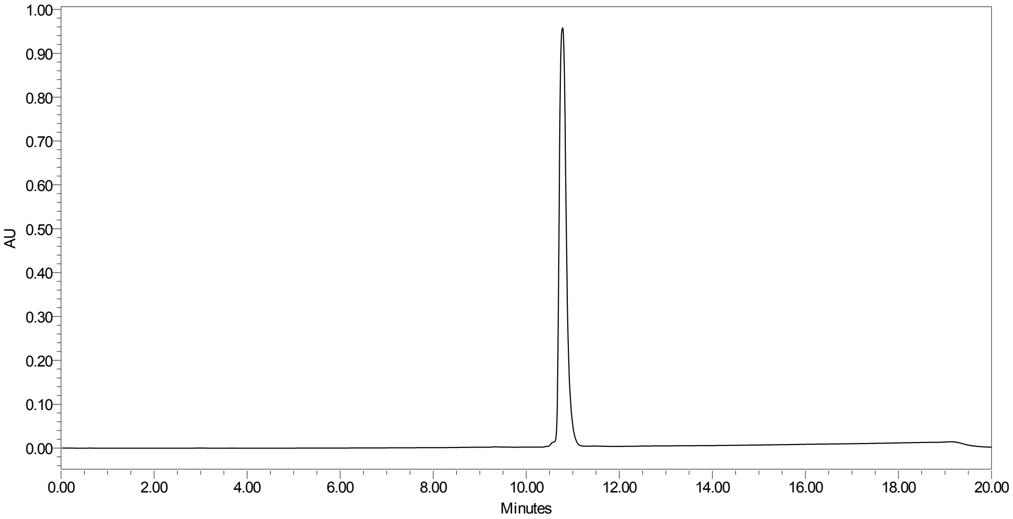

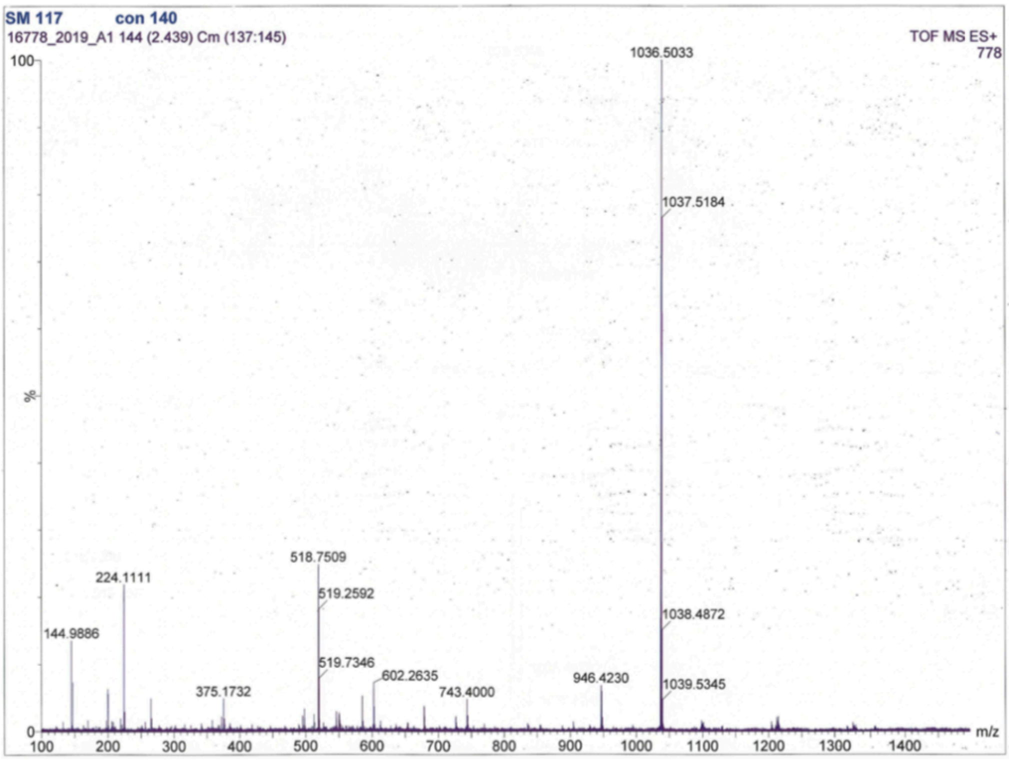

SMXI18, Ac-Lys(2-Cl-Z)-2-Aoc-His(Bzl)-Arg-ACC

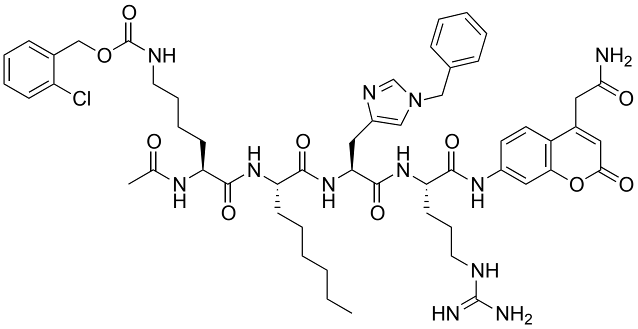

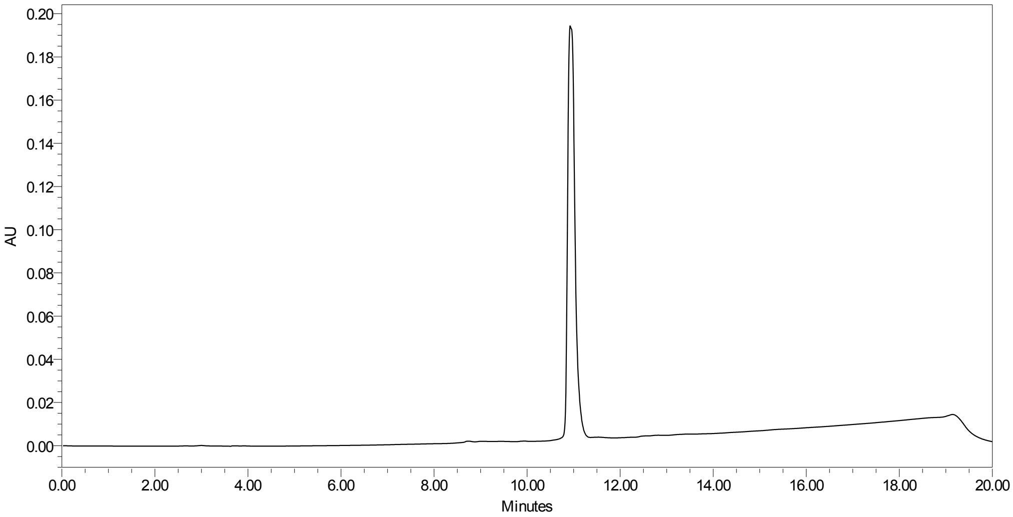

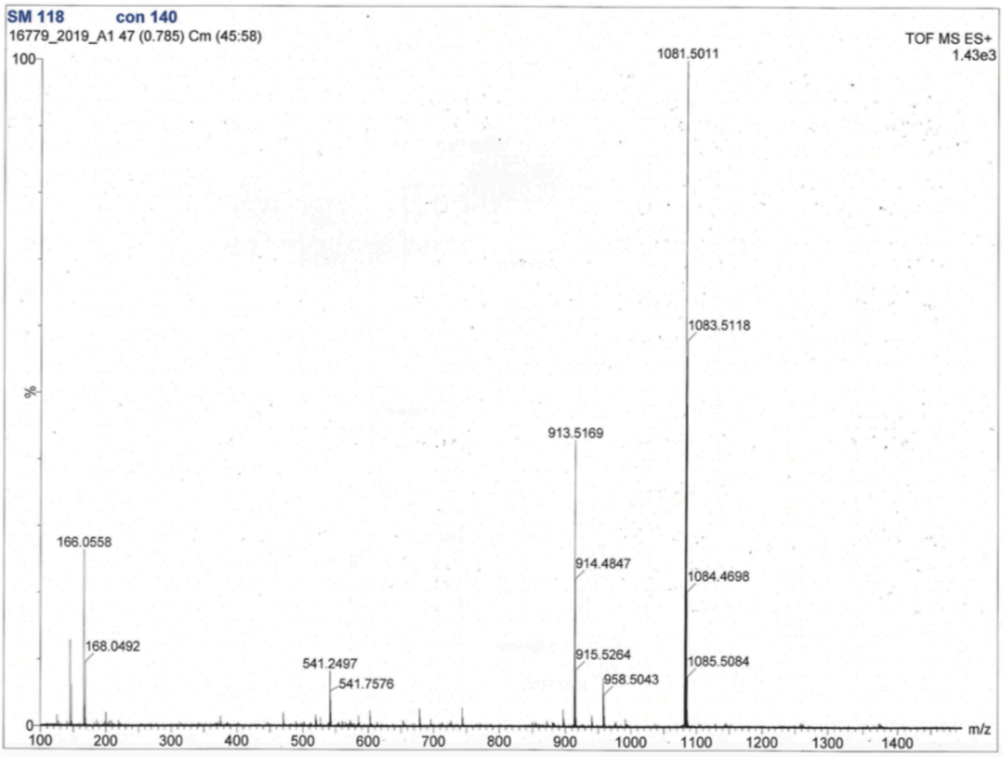

SMXI19, Ac-Tyr(2,6-Cl_2-Z)-Dht-His(Bzl)-Arg-ACC

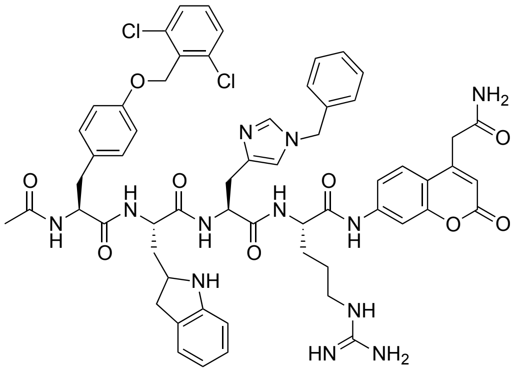

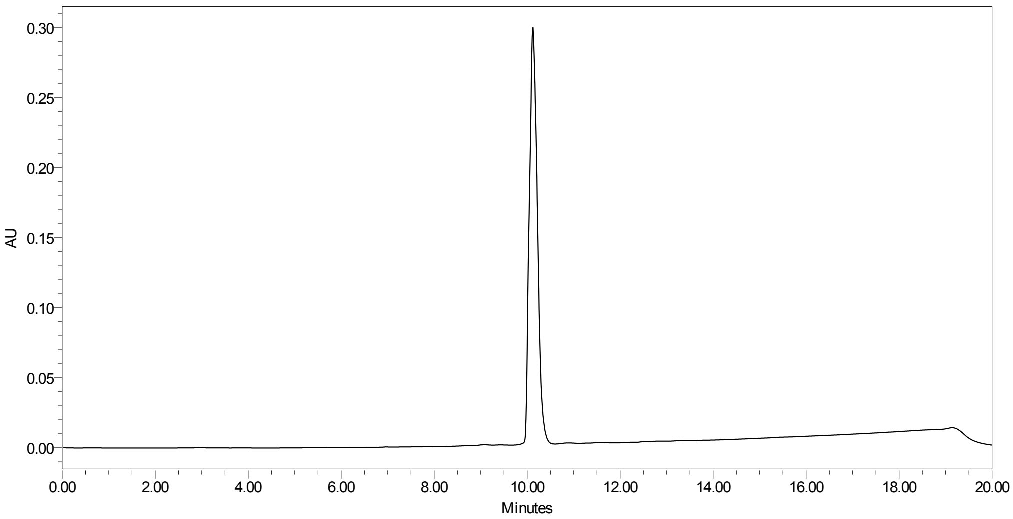

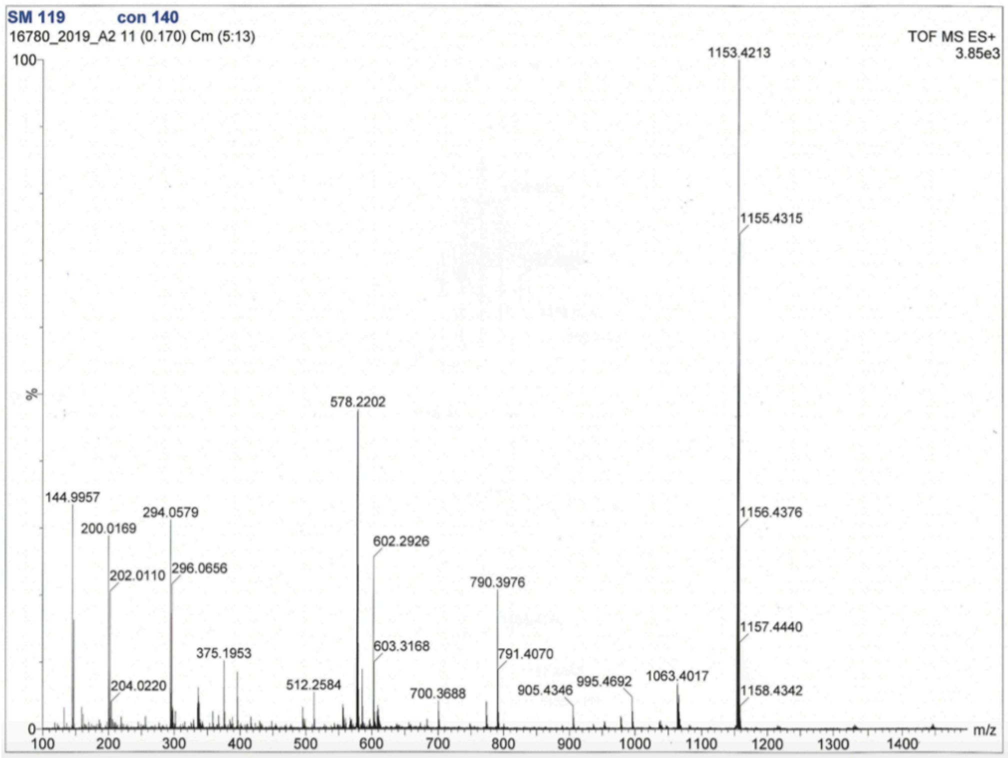

SMXI20, Ac-Tyr(2,6-Cl_2-Z)-Cha-His(Bzl)-Arg-ACC

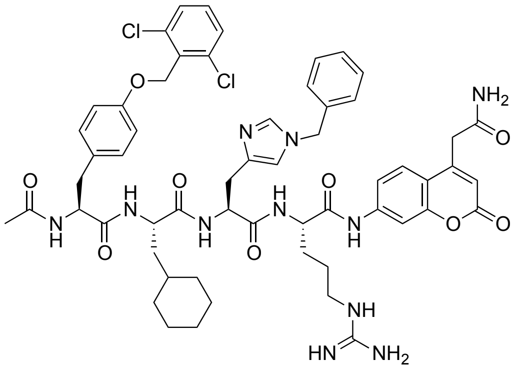

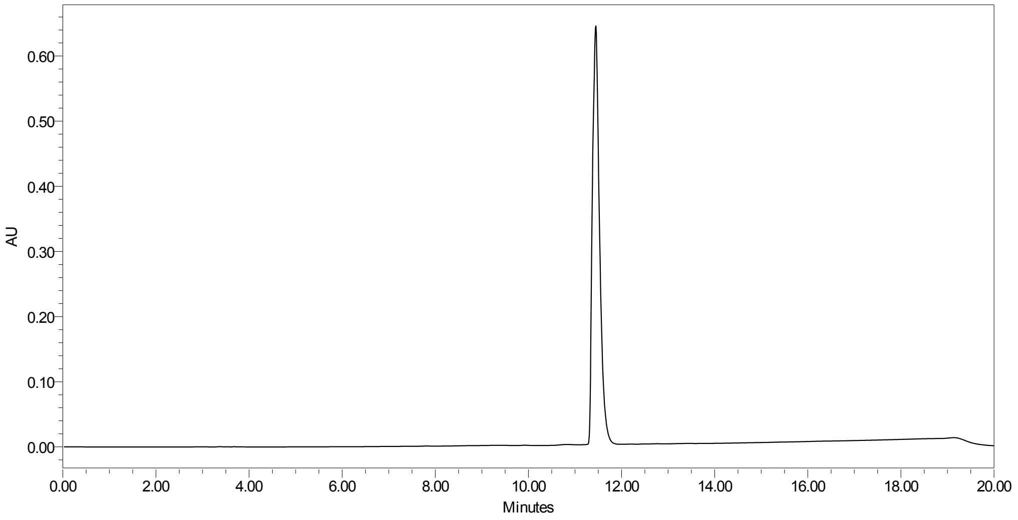

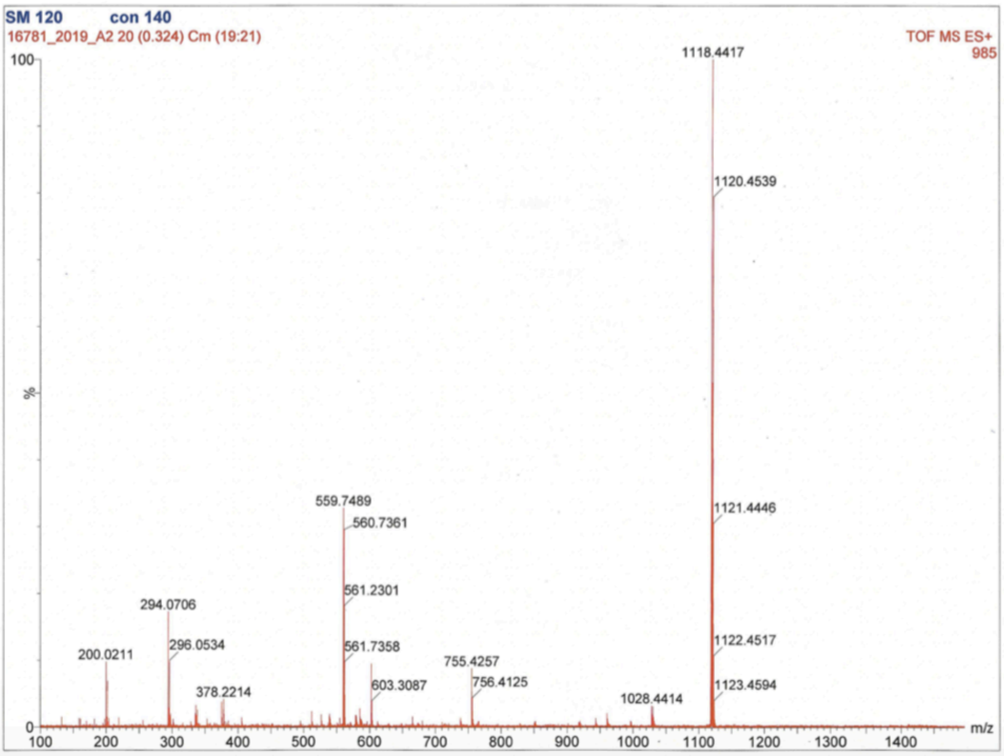

SMXI21, Ac-Tyr(2,6-Cl_2-Z)-hCha-His(Bzl)-Arg-ACC

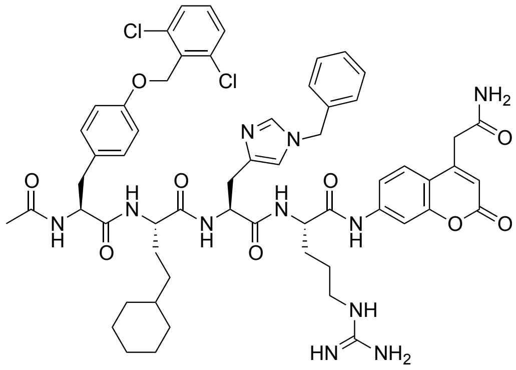

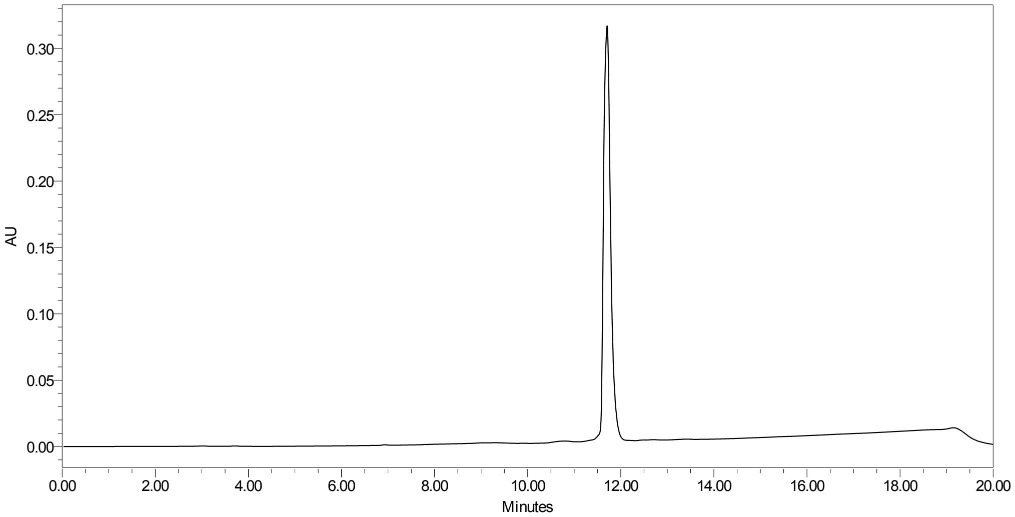

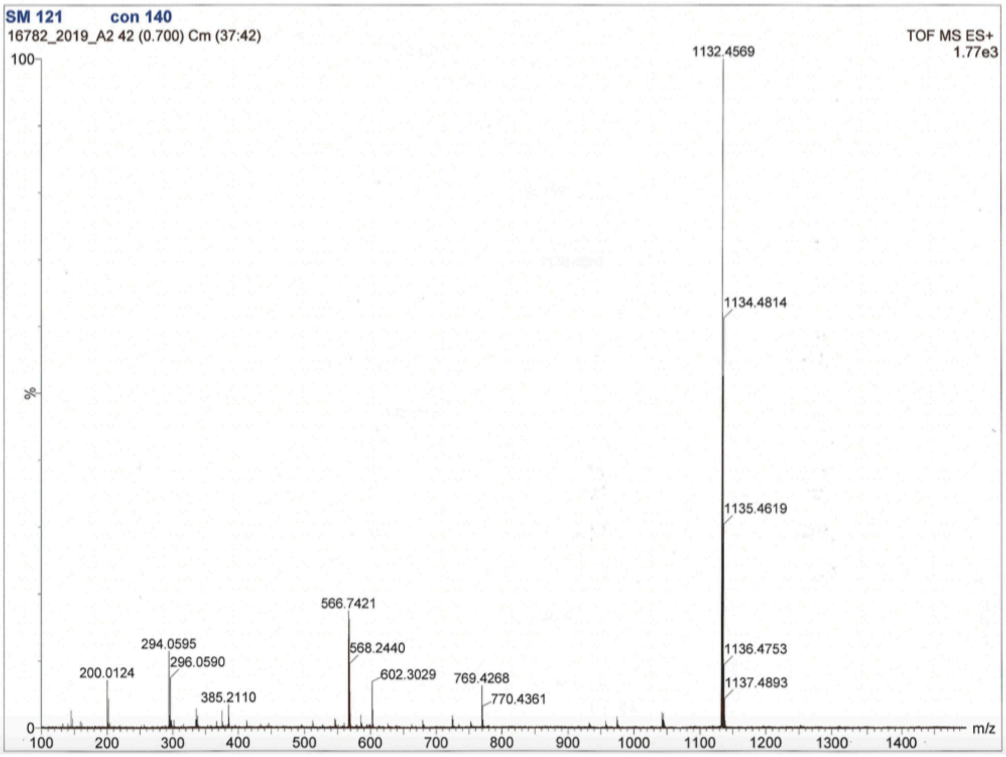

SMXI22, Ac-Tyr(2,6-Cl_2-Z)-2-Aoc-Nle(O-Bzl)-Arg-ACC

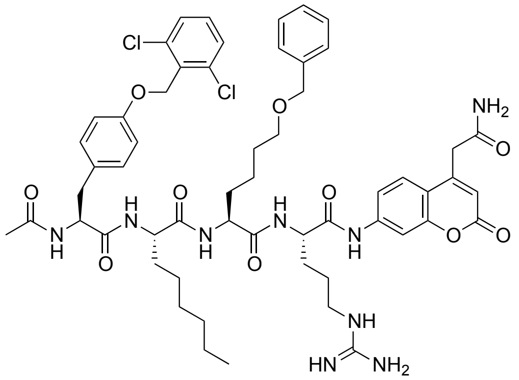

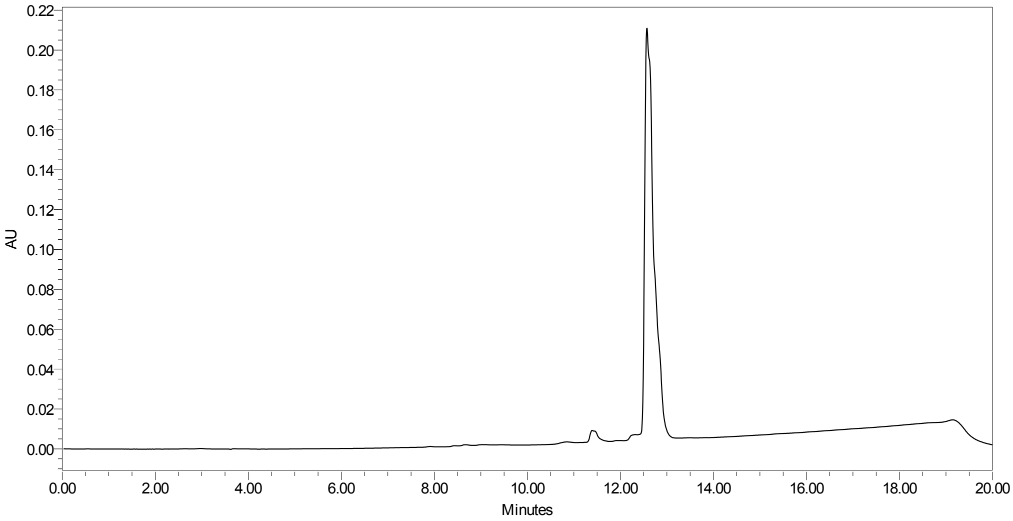

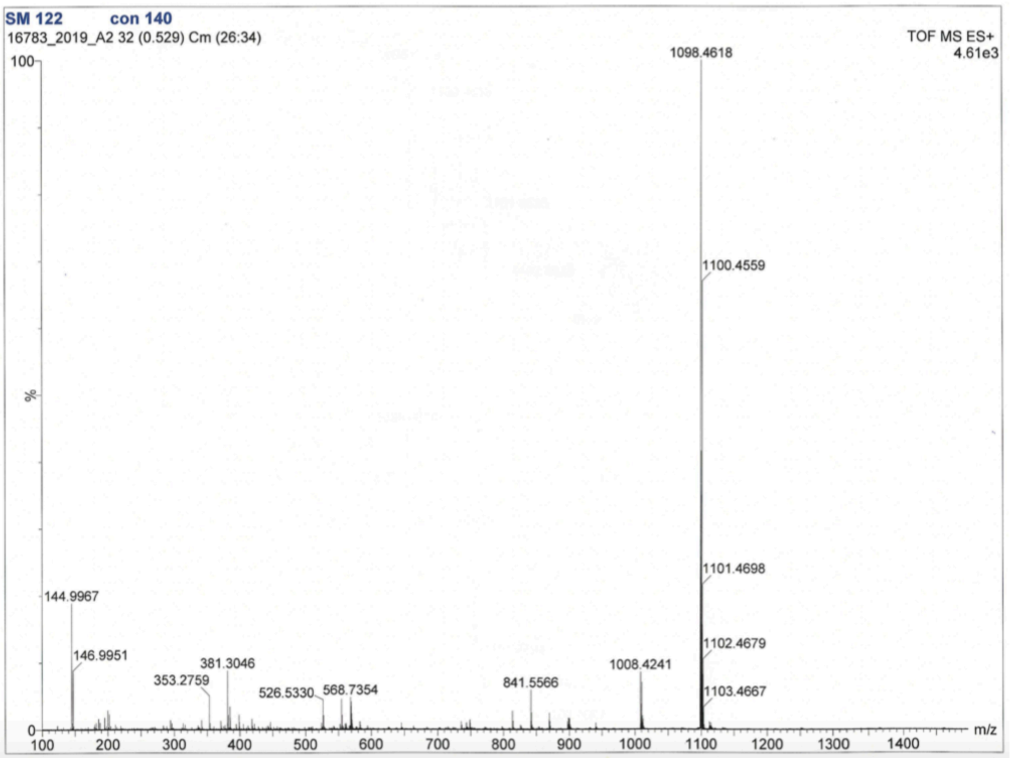

SMXI23, Ac-Tyr(2,6-Cl_2-Z)-2-Aoc-hSer(Bzl)-Arg-ACC

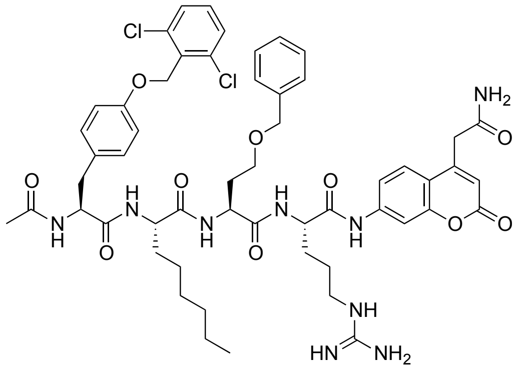

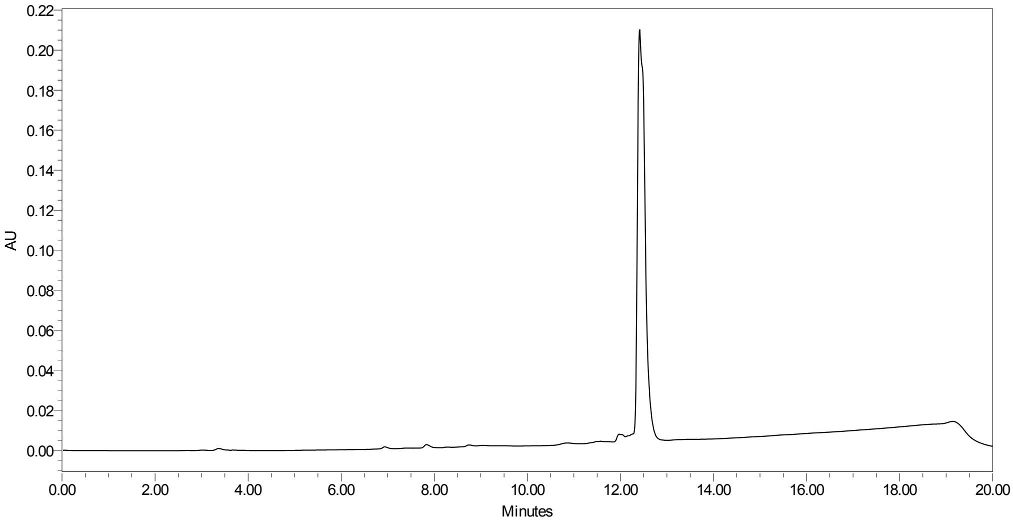

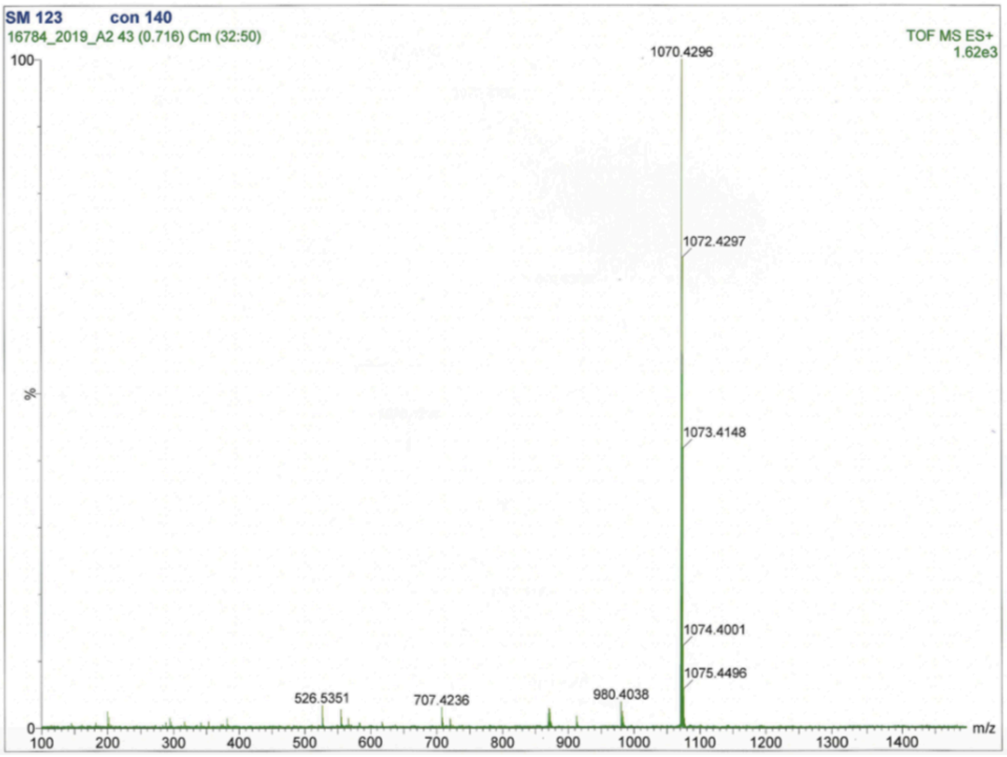

SMXI24, Ac-Ile-Nle-Asn-Arg-ACC

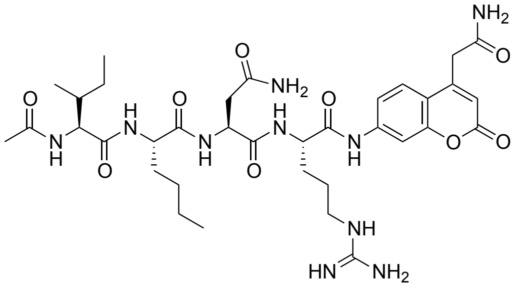

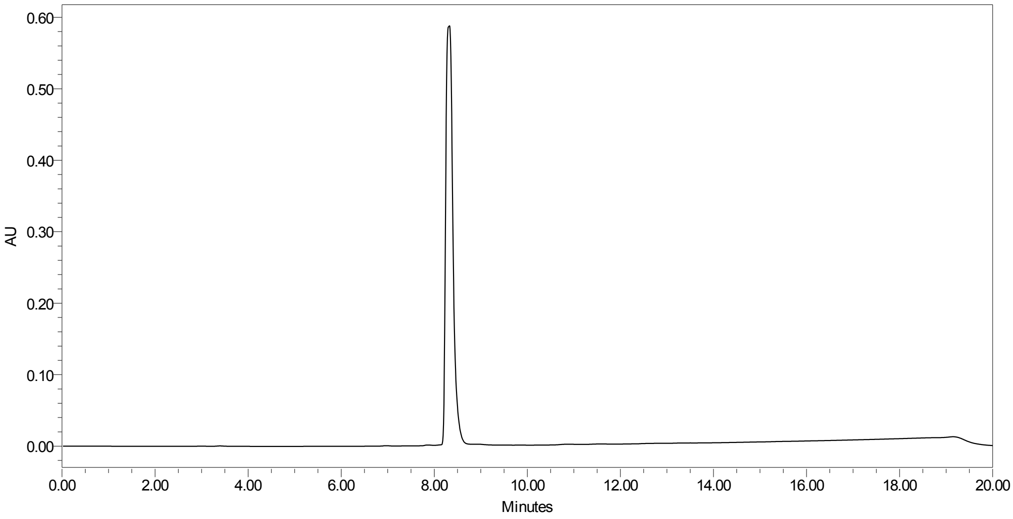

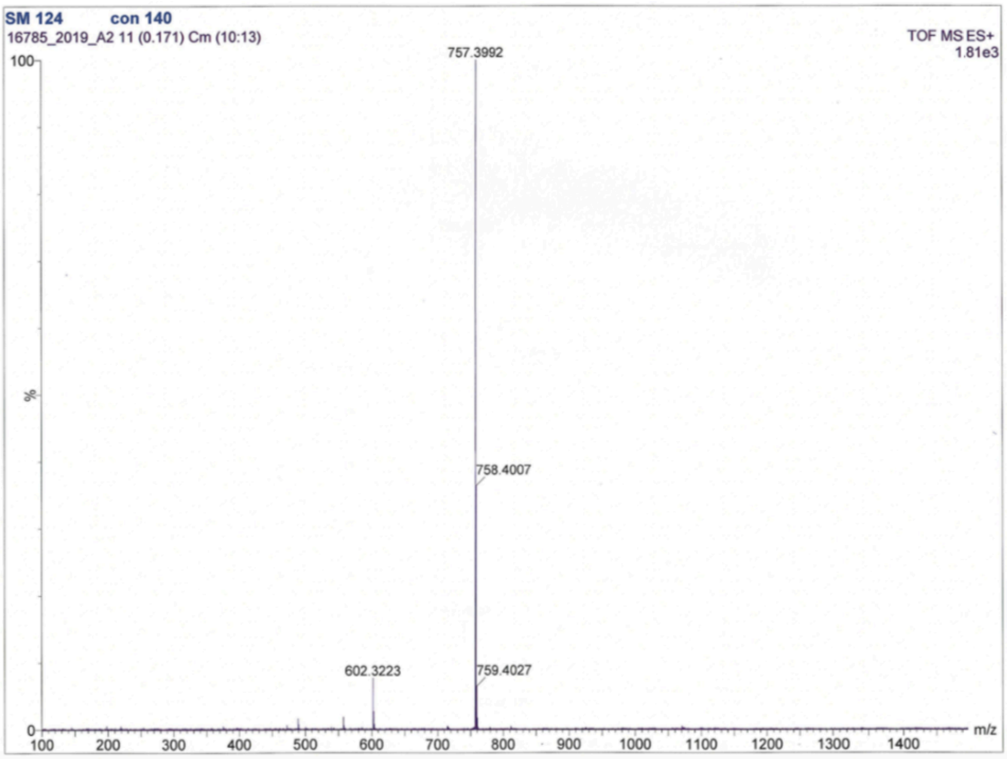

P-SMXI51, biotin-PEG(4)-Tyr(2,6-Cl_2-Z)-Nle-Glu(Bzl)-$Arg^P(OPh)_2$

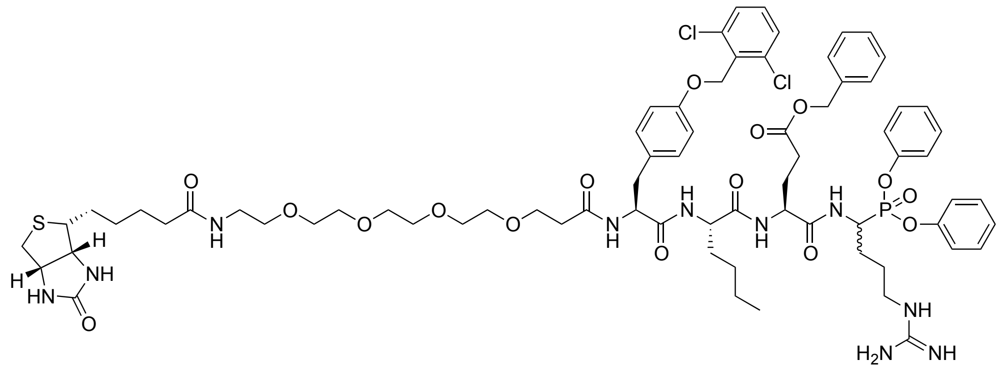

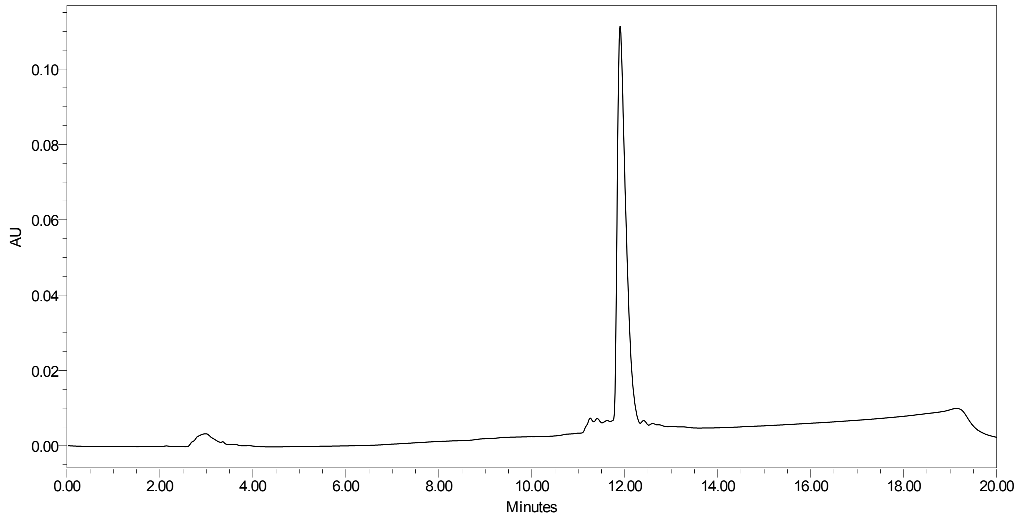

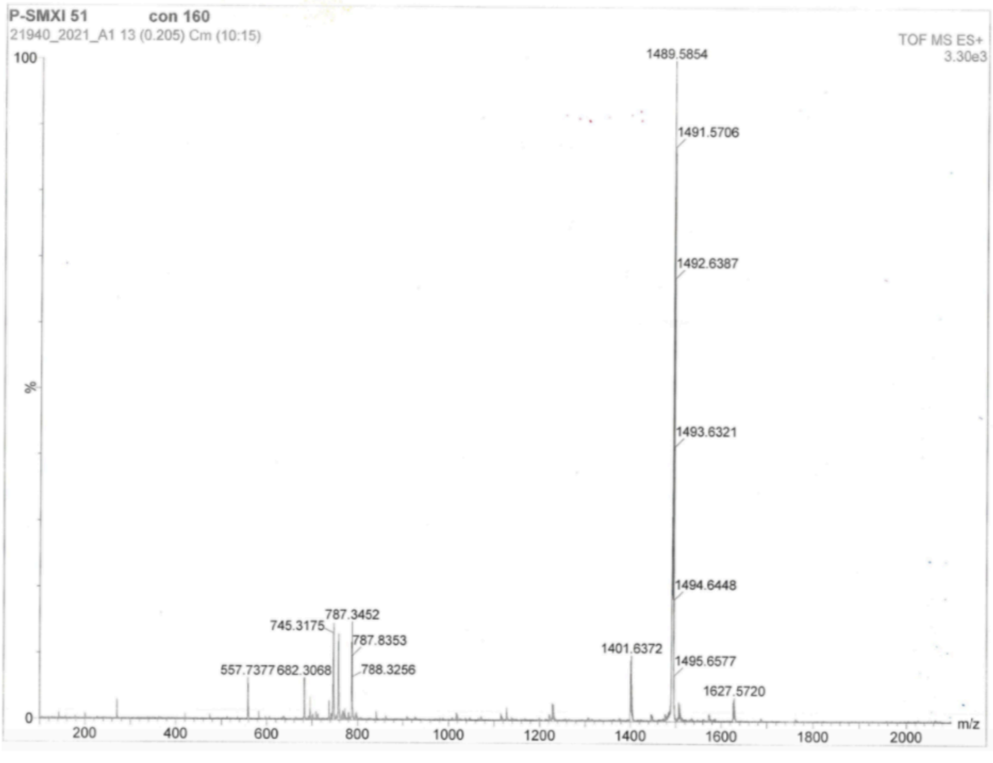

P-SMXI191, biotin-PEG(4)-Tyr(2,6-Cl_2-Z)-Dht-His(Bzl)-Arg^P(OPh)$_2$

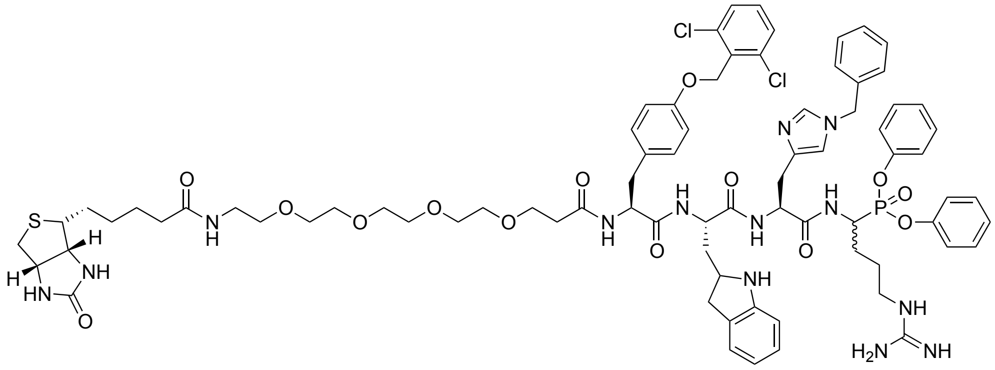

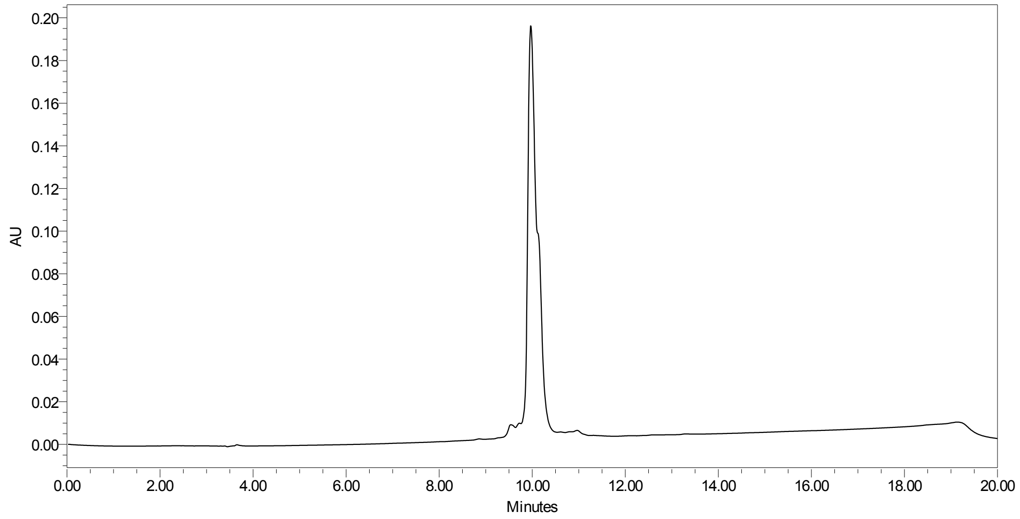

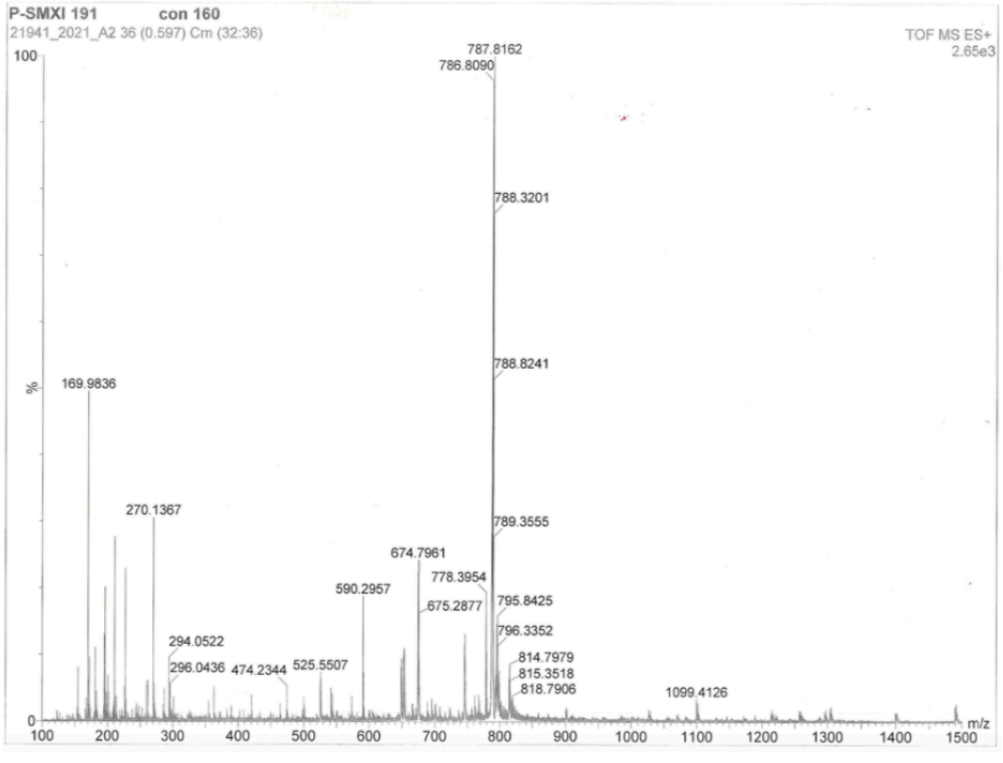

P-SMXI52, BODIPY-PEG(4)-Tyr(2,6-Cl_2-Z)-Nle-Glu(Bzl)-$Arg^P(OPh)_2$

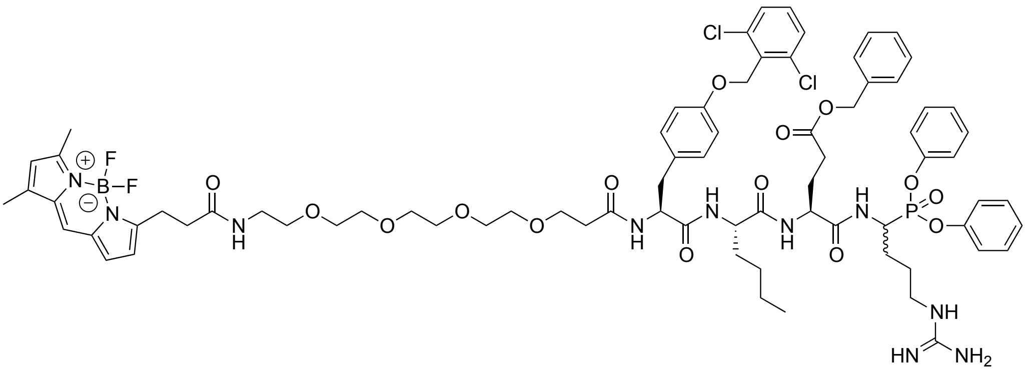

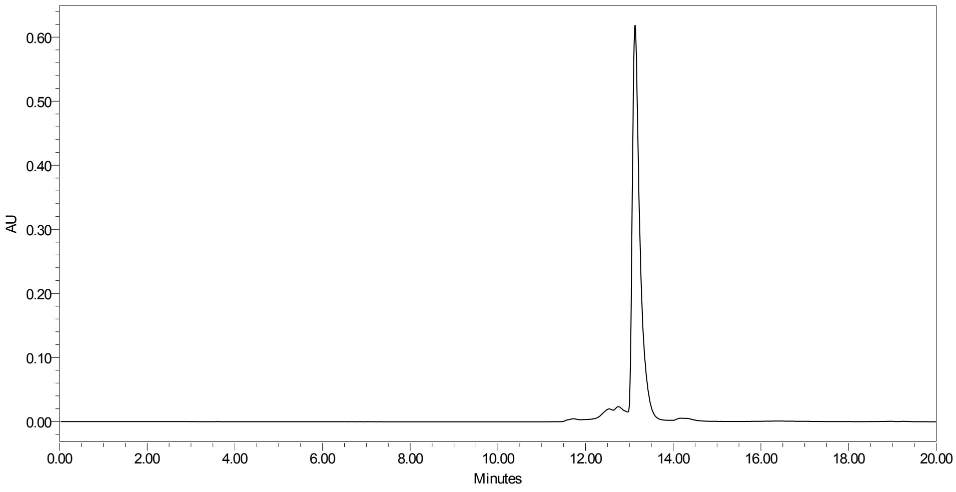

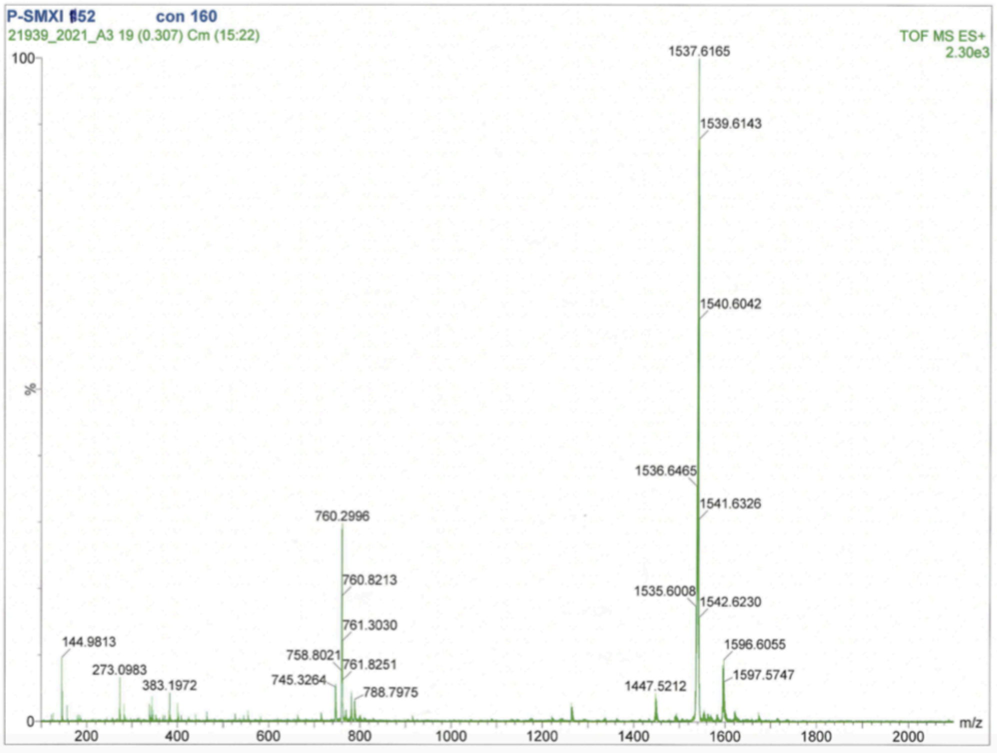

I-SMXI5, Ac-Tyr(2,6-Cl_2-Z)-Nle-Glu(Bzl)-$Arg^P(OPh)_2$

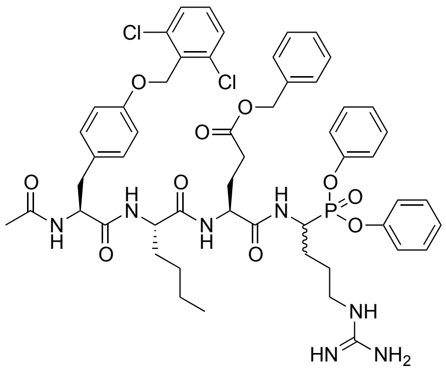

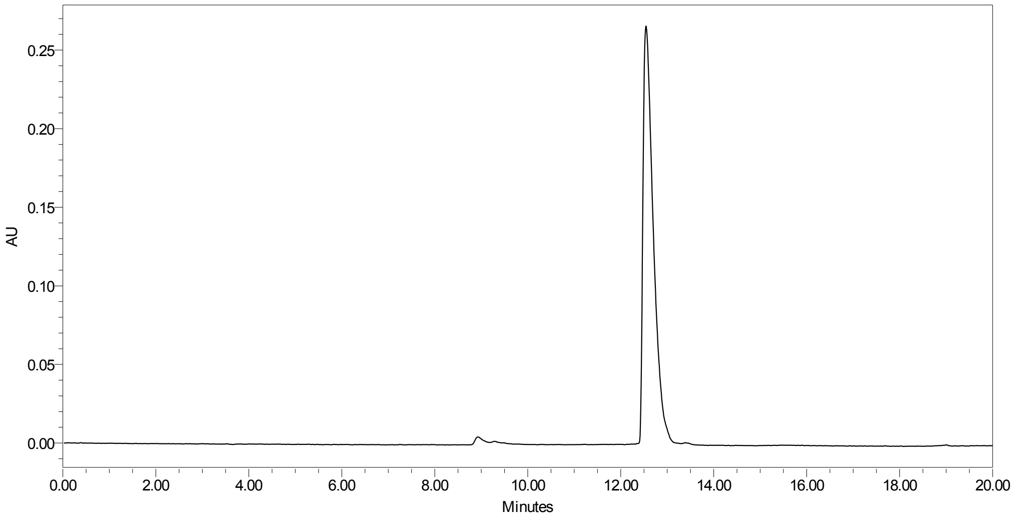

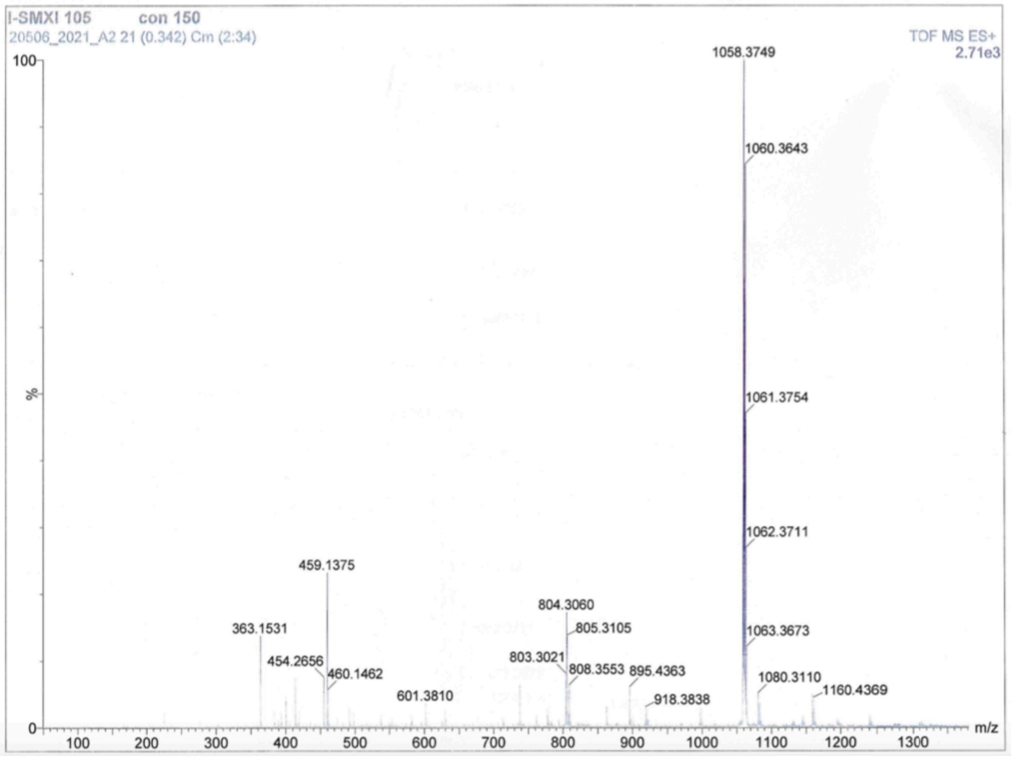

The chromatograms of some substrates are not sharp but appear as two peaks. This was the case with the two sets of substrates listed below in the Table S2. What they have in common is the fact that they all have the amino acid Glu(Bzl) at the P2 position. A series of the same substrates differing only at the P2 position (His(Bzl) instead of Glu(Bzl)) no longer give such a false double peak. We believe that this is due to their structure, namely the interactions of large hydrophobic amino acids at positions P4 and P2 with the stationary phase.

To more closely explain the fact that the double peak does not affect the purity, we have presented the close-up of spectra and the MS result from the beginning and the end of the peak of two exemplary substrates from two series – SMXI6 and SMXI13 (the situation for the rest of the substrates was the same). It can be noticed that the masses of both peaks are the same, which means that the compound is pure, and only its separation on the column gives two separate – although essentially the same peaks. Proper masses were also confirmed by the HRMS analysis.

Table S2. Substrates with Glu(Bzl) at the P2 position.

	**Structure**					
		**P4**	**P3**	**P2**	**P1**	
**SMXI5**	Ac	Tyr(2,6-Cl_2-Z)	Nle	Glu(Bzl)	Arg	ACC
**SMXI6**	Ac	Tyr(2,6-Cl_2-Z)	Phe(3-F)	Glu(Bzl)	Arg	ACC
**SMXI7**	Ac	Tyr(2,6-Cl_2-Z)	2-Aoc	Glu(Bzl)	Arg	ACC
**SMXI8**	Ac	Tyr(2,6-Cl_2-Z)	Tyr(2,6-Cl_2-Z)	Glu(Bzl)	Arg	ACC
**SMXI13**	Ac	Tyr(Bzl)	Nle	Glu(Bzl)	Arg	ACC
**SMXI14**	Ac	Tyr(Bzl)	Phe(3-F)	Glu(Bzl)	Arg	ACC
**SMXI15**	Ac	Tyr(Bzl)	2-Aoc	Glu(Bzl)	Arg	ACC
**SMXI16**	Ac	Tyr(Bzl)	Tyr(2,6-Cl_2-Z)	Glu(Bzl)	Arg	ACC

Close-up of spectra

SMXI6

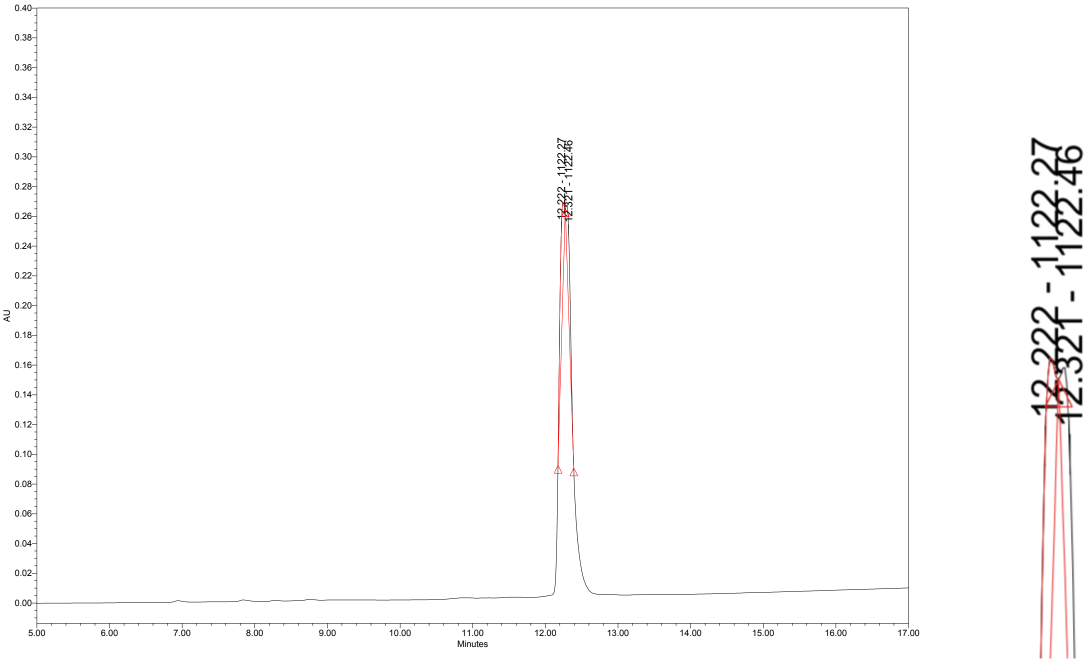

SMXI13

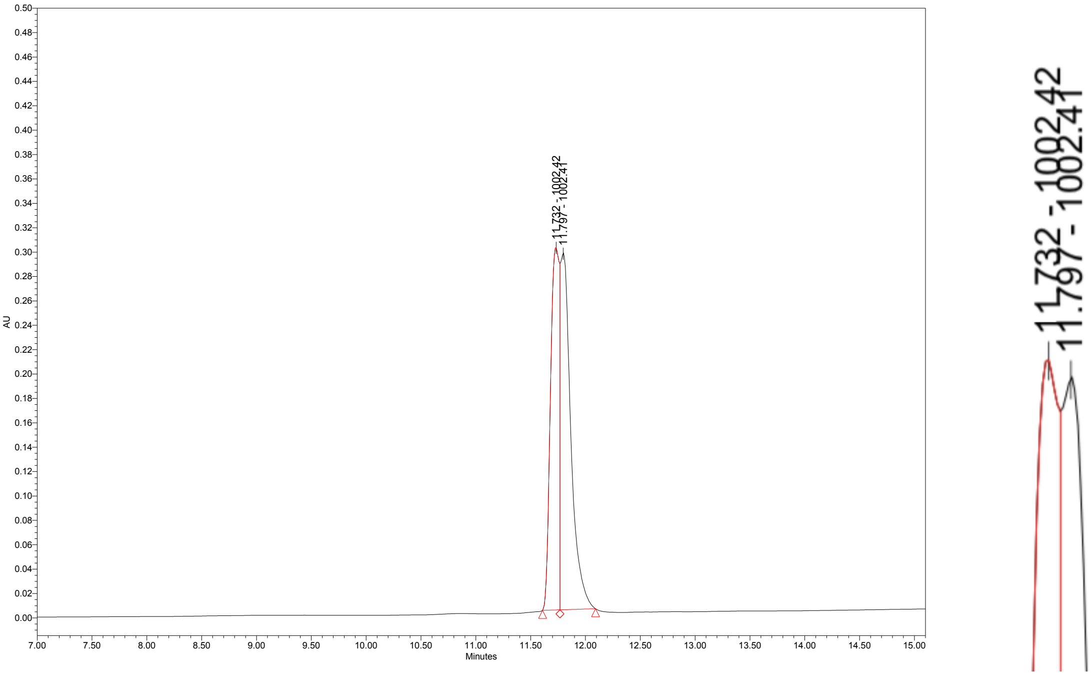

MS results from the beginning and the end of the peak

SMXI6

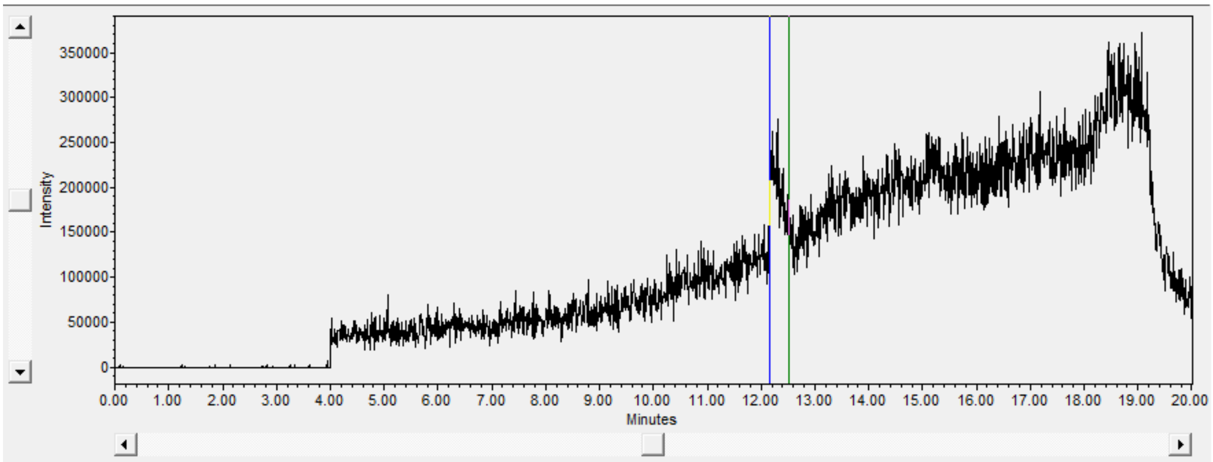

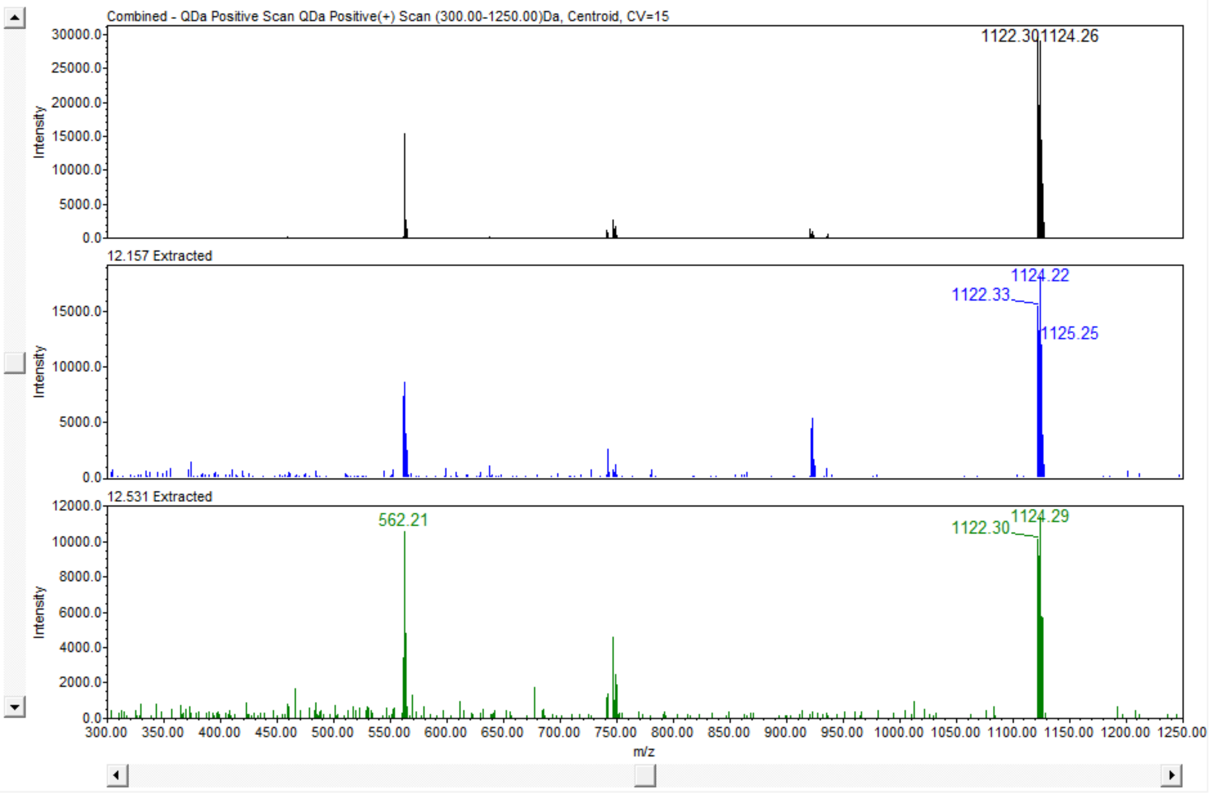

	Image	Select	Retention Time (min)	Scan Number	Name	Info	Description	Combine Type	Baseline Correction	Noise Threshold (%)	Peak Separation (Da)	Max. Intensity (Intensity)	Base Peak (m/z)	Start Mass (m/z)	End Mass (m/z)
1		☑				Combined	Baseline Correction: Off (12.13:12.54)	Average	☐	0.000	1.0000	29763.895391	1122.30	300.00	1250.00
2		☑	12.157	1460		Extracted	Baseline Correction: Off (12.14:12.17)	Average	☐	0.000	1.0000	18261.139844	1124.22	300.00	1250.00
3		☑	12.531	1505		Extracted	Baseline Correction: Off (12.51:12.55)	Average	☐	0.000	1.0000	11468.578125	1124.29	300.00	1250.00

SMXI13

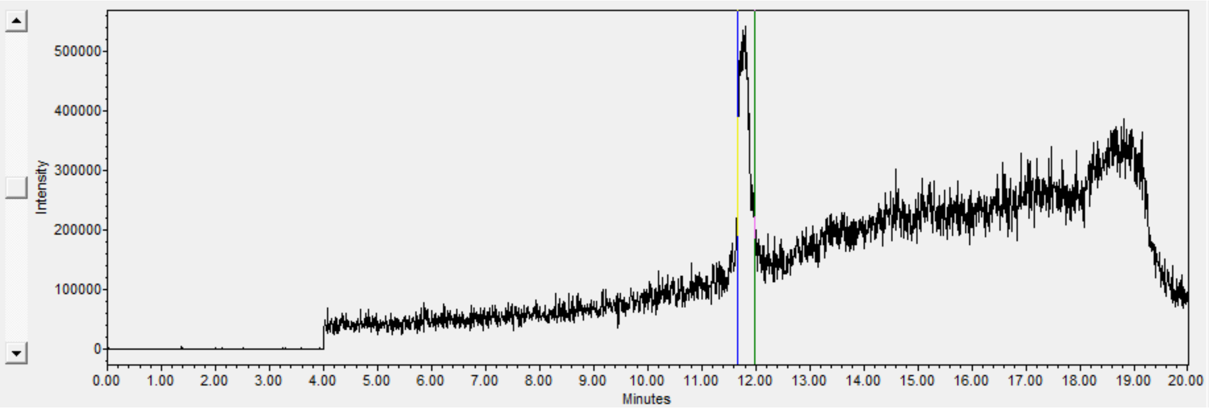

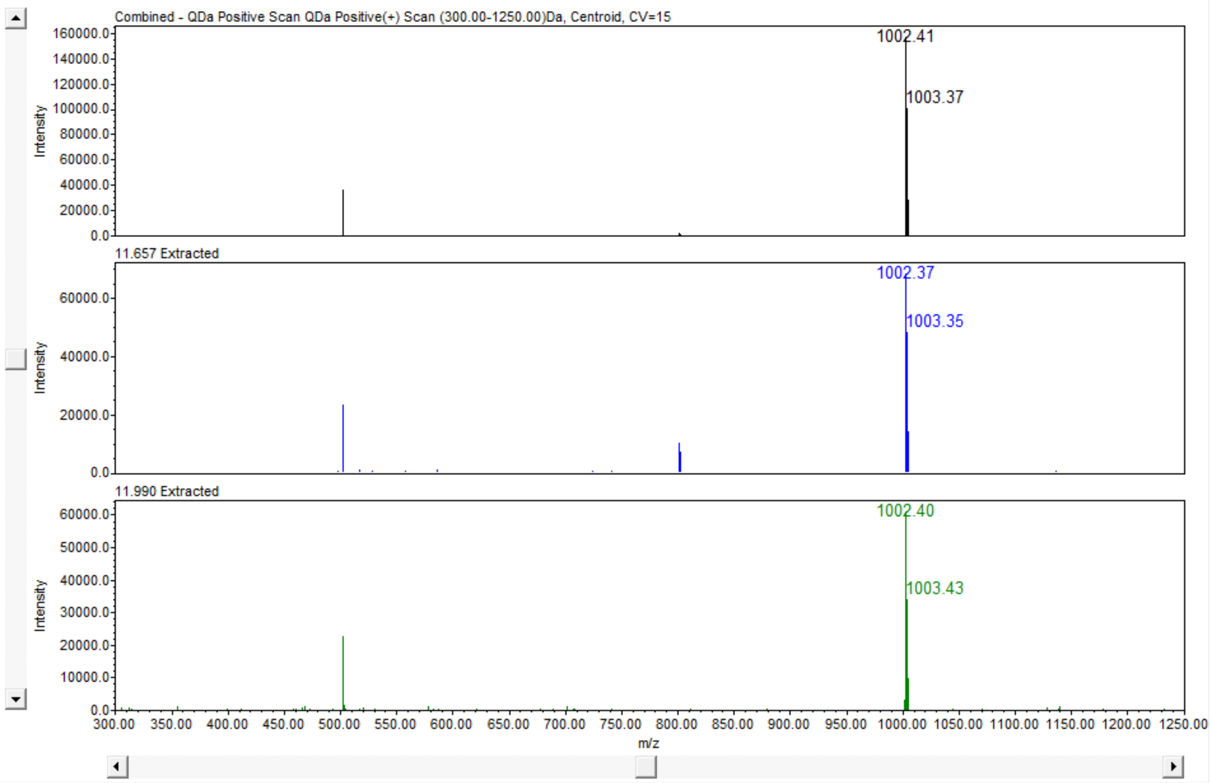

	Image	Select	Retention Time (min)	Scan Number	Name	Info	Description	Combine Type	Baseline Correction	Noise Threshold (%)	Peak Separation (Da)	Max. Intensity (Intensity)	Base Peak (m/z)	Start Mass (m/z)	End Mass (m/z)
1		☑				Combined	Baseline Correction: Off (11.64:11.98)	Average	☐	0.000	1.0000	157756.688988	1002.41	300.00	1250.00
2		☑	11.657	1400		Extracted	Baseline Correction: Off (11.64:11.67)	Average	☐	0.000	1.0000	68610.753125	1002.37	300.00	1250.00
3		☑	11.990	1440		Extracted	Baseline Correction: Off (11.97:12.01)	Average	☐	0.000	1.0000	61505.784375	1002.40	300.00	1250.00

NMR analysis of P-SMXI51, P-SMXI52, and I-SMXI5

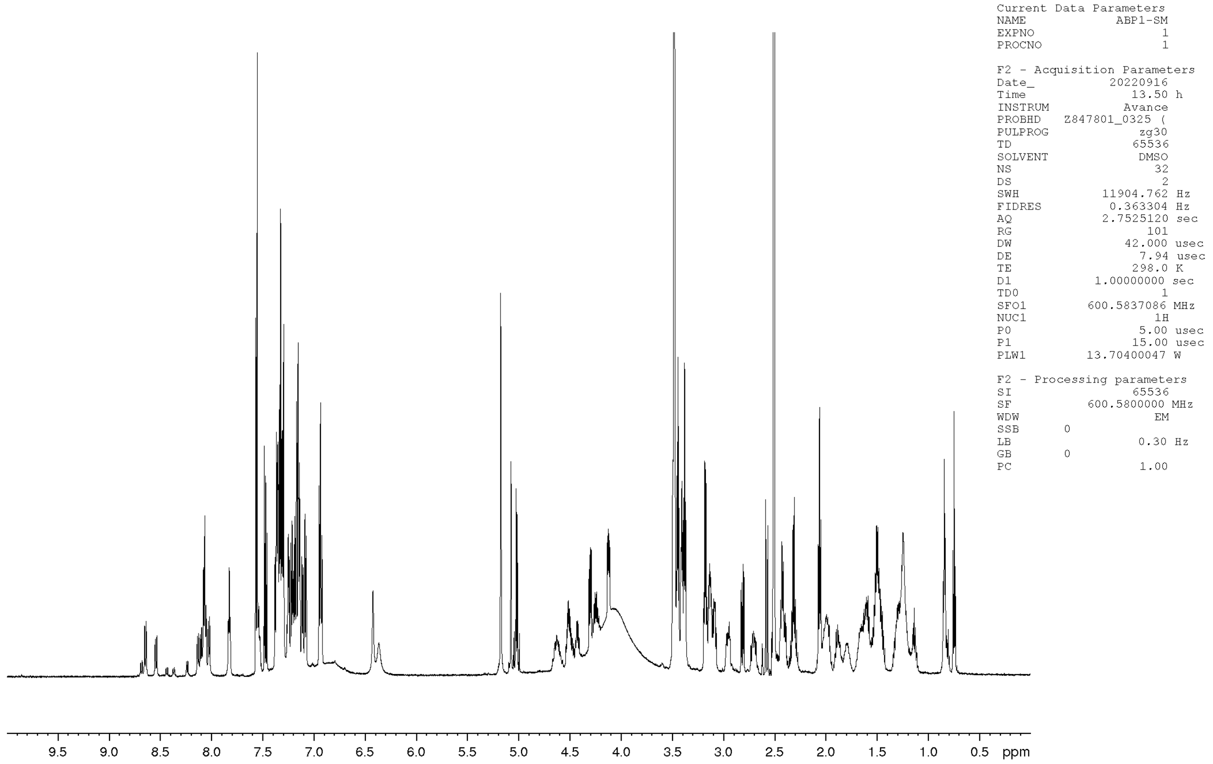

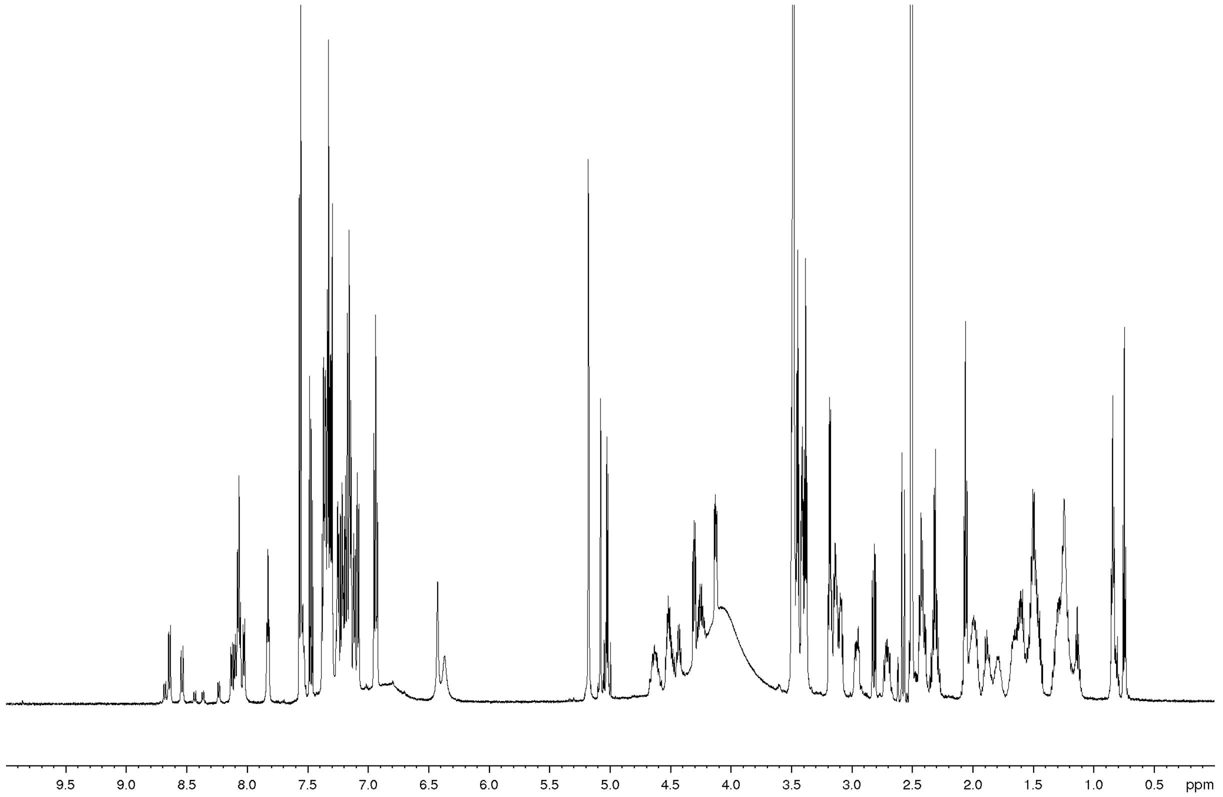

^{1}H NMR spectrum of P-SMXI51 (600 MHz, DMSO-d_6).

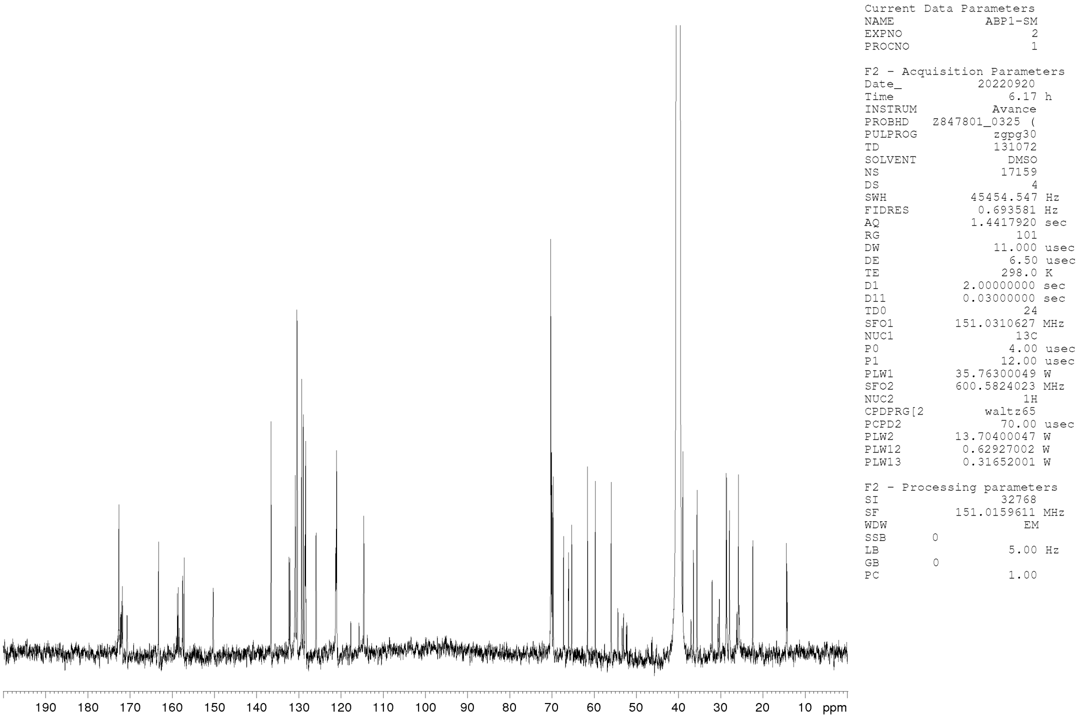

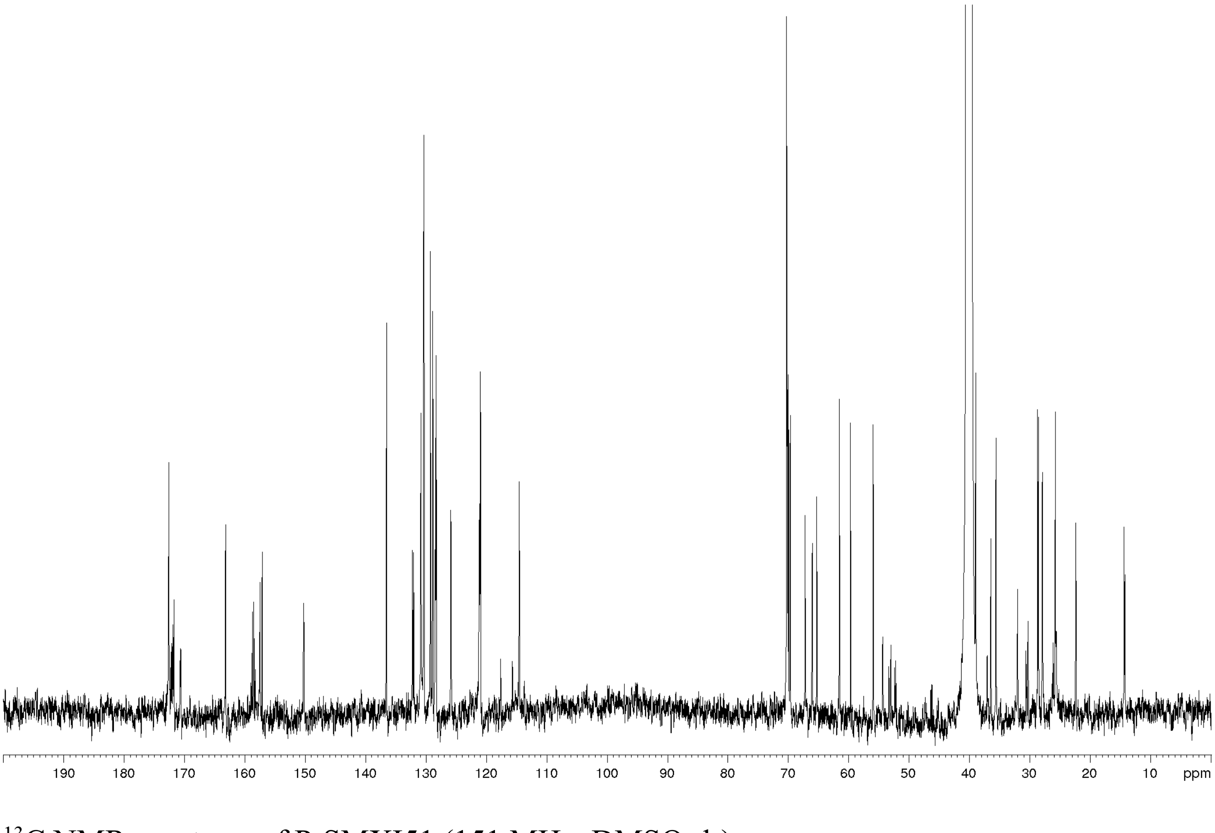

^{13}C NMR spectrum of P-SMXI51 (151 MHz, DMSO-d_6).

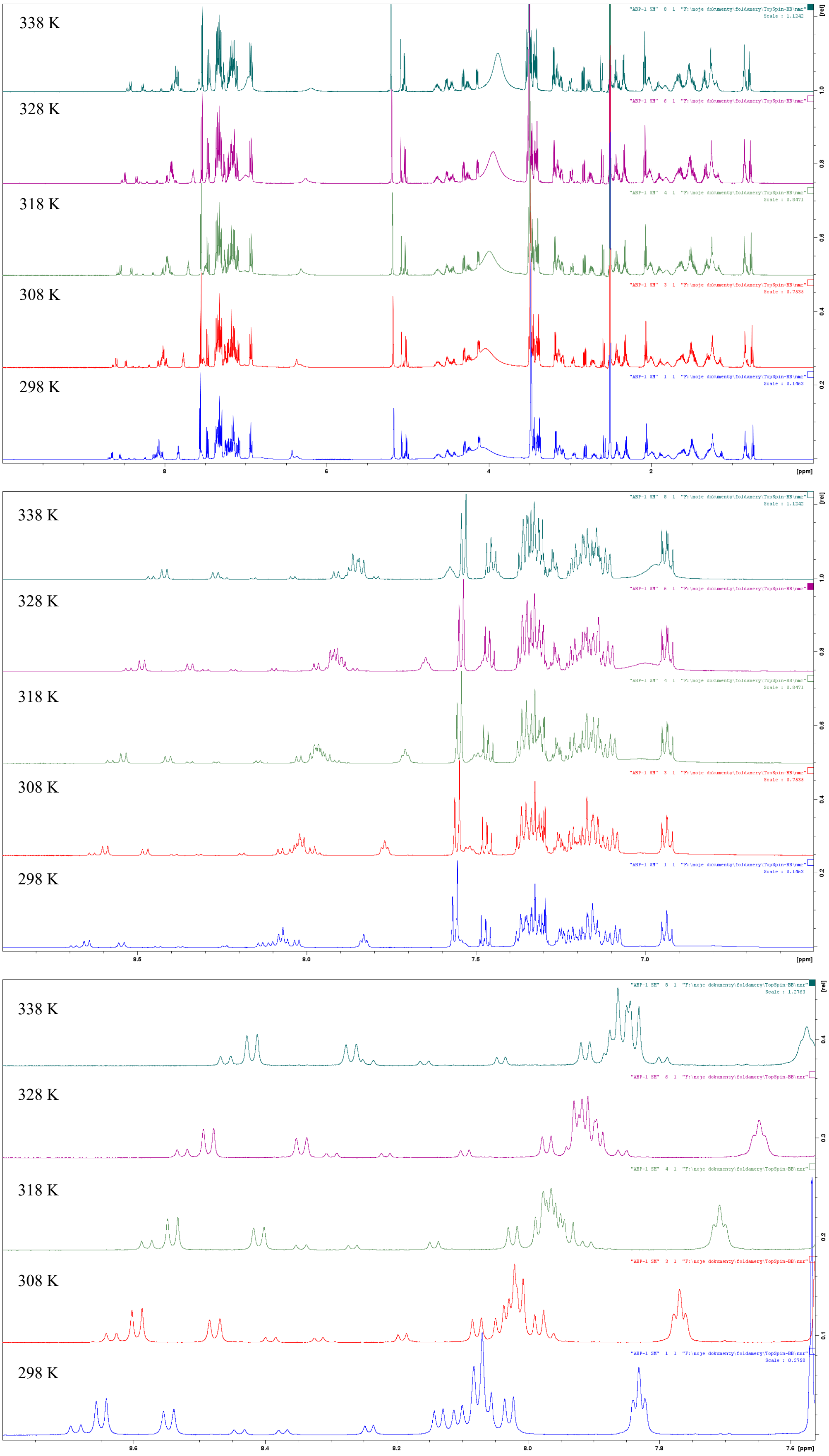

VT NMR spectrum of P-SMXI51 (600 MHz, DMSO-d_6).

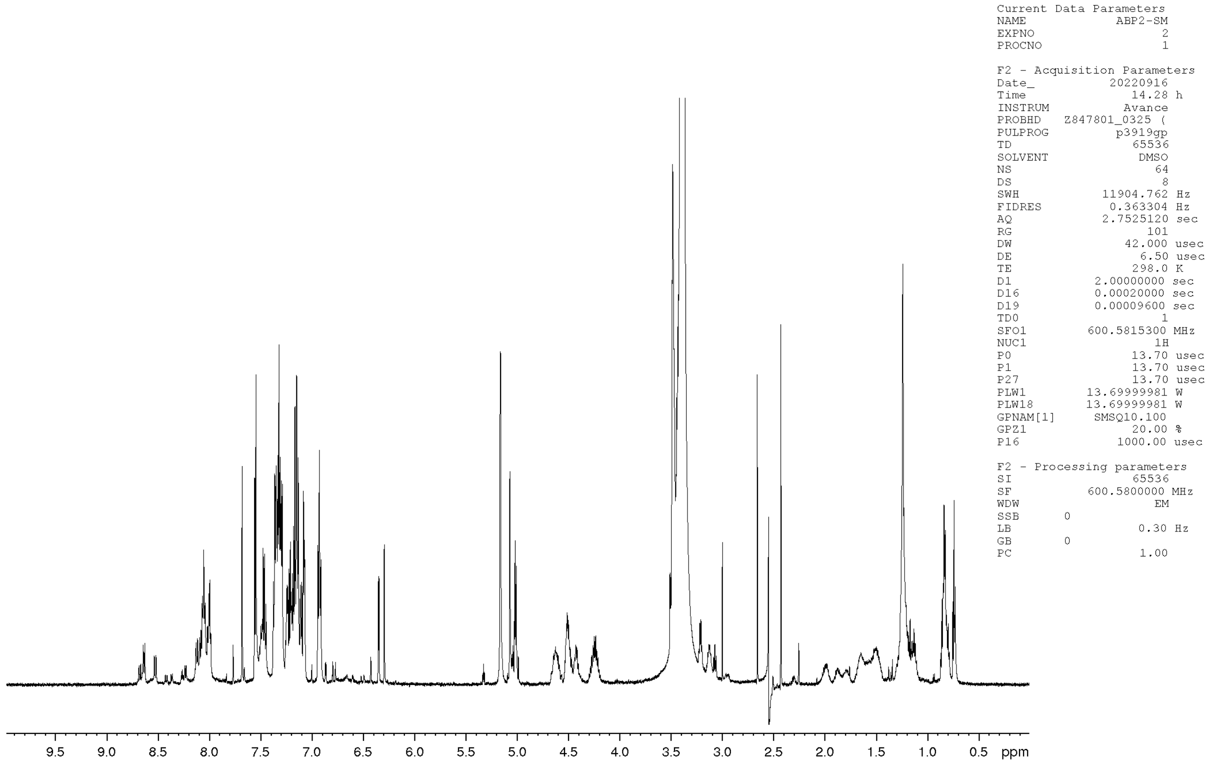

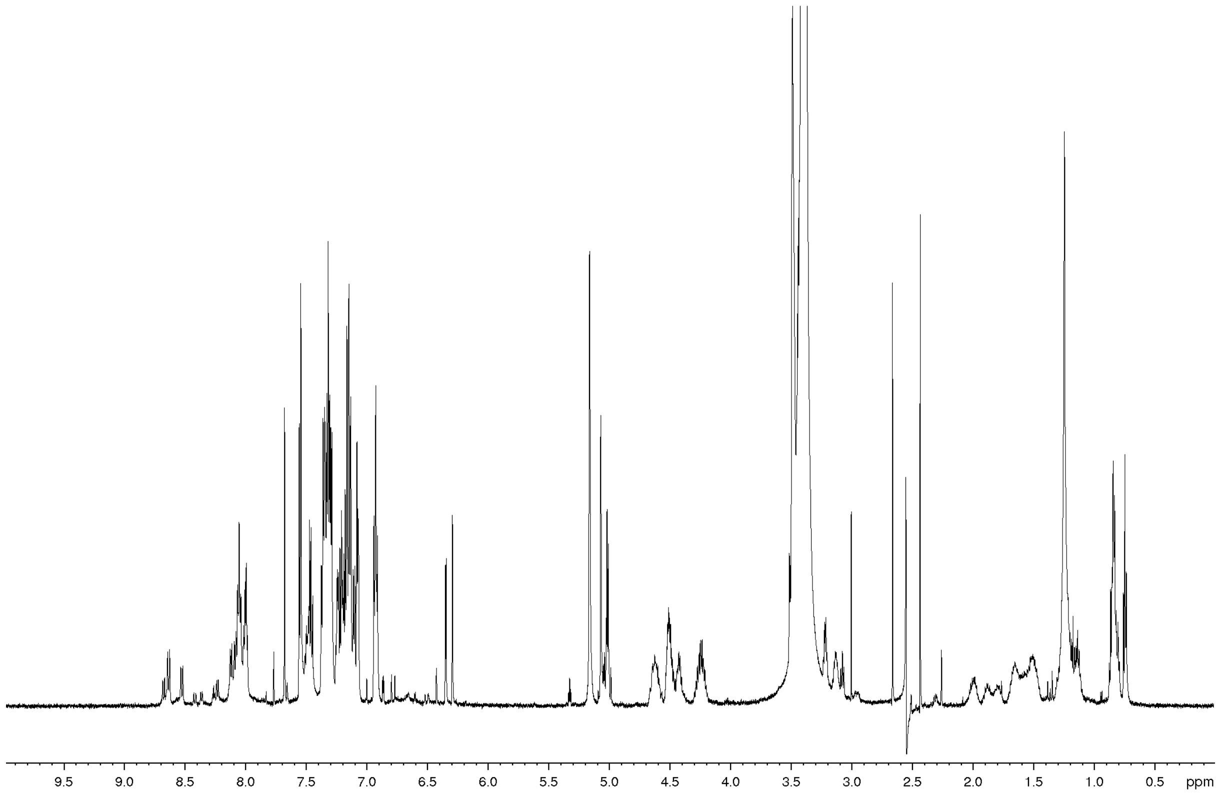

^{1}H NMR spectrum of P-SMXI52 (600 MHz, DMSO-d_6).

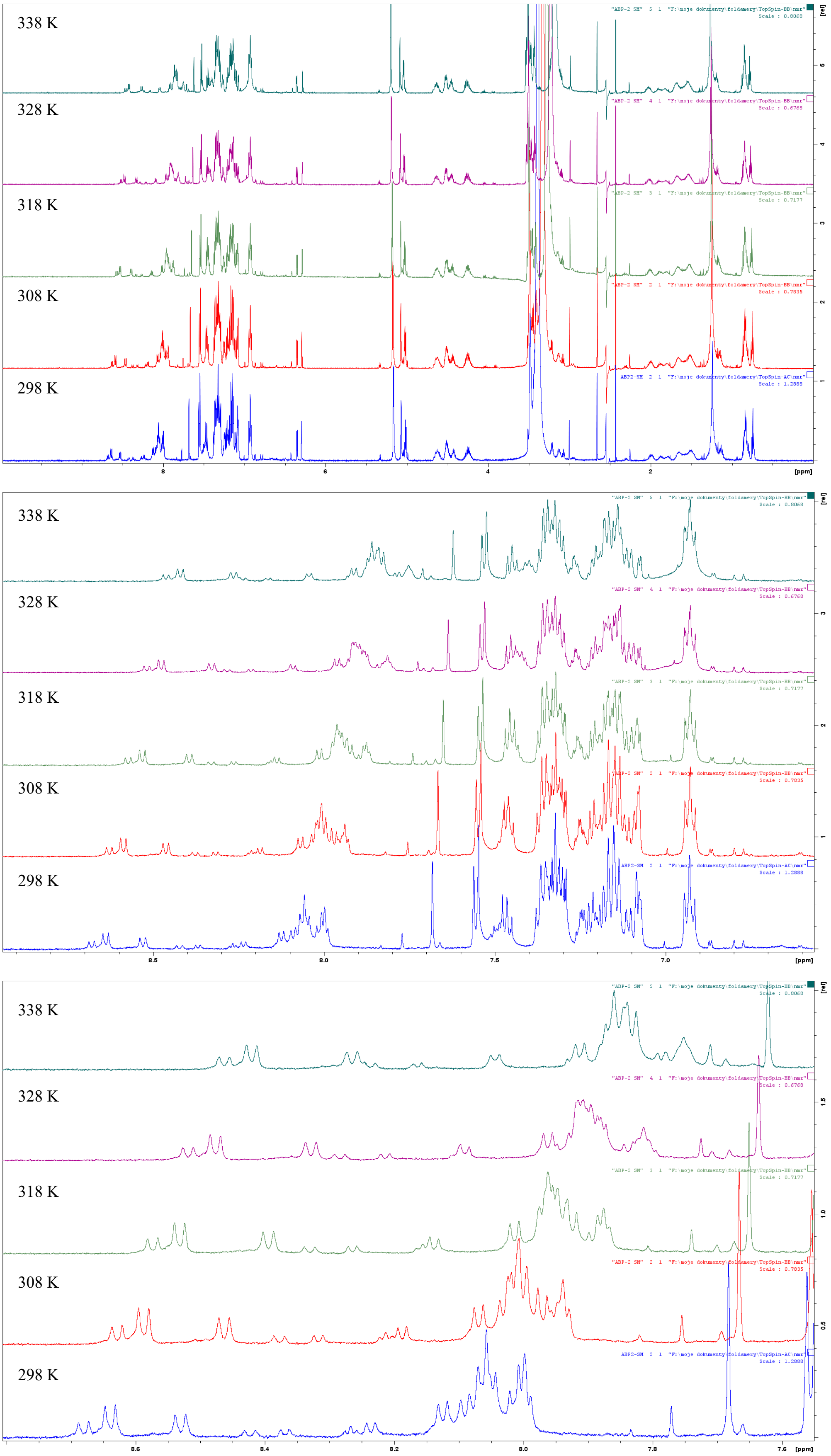

VT NMR spectrum of P-SMXI52 (600 MHz, DMSO-d_6).

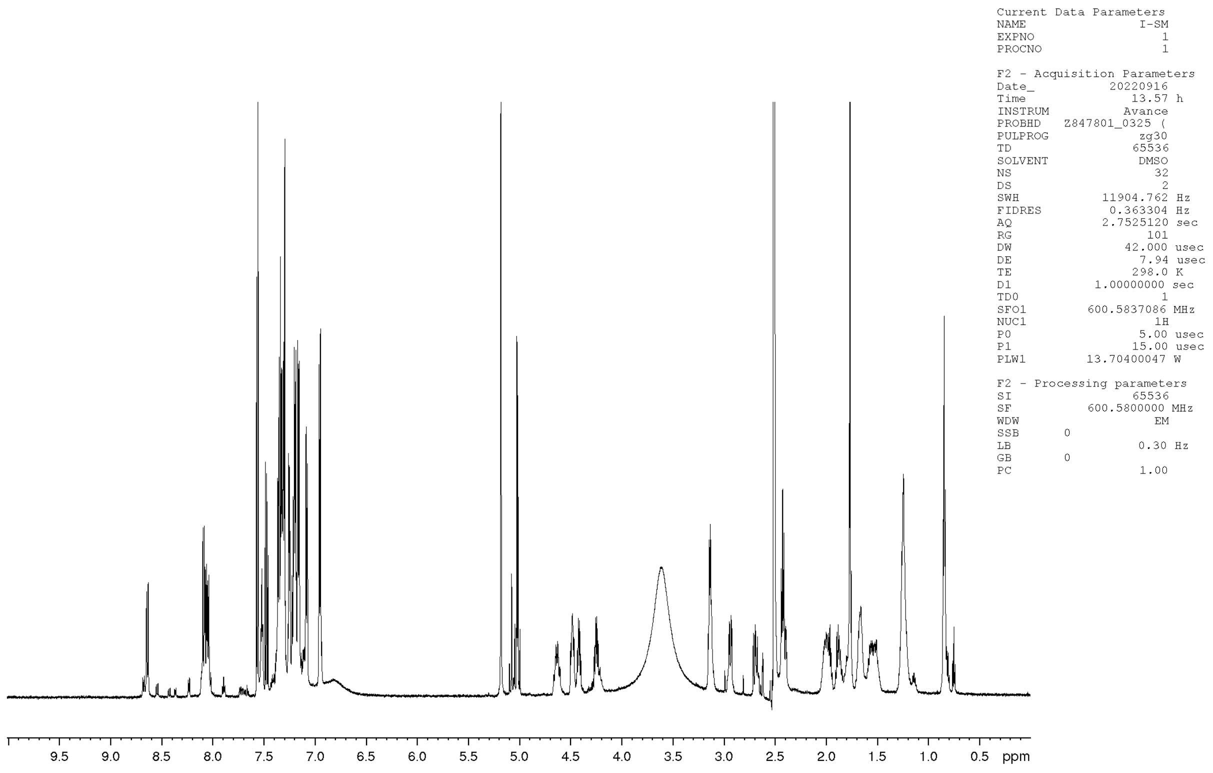

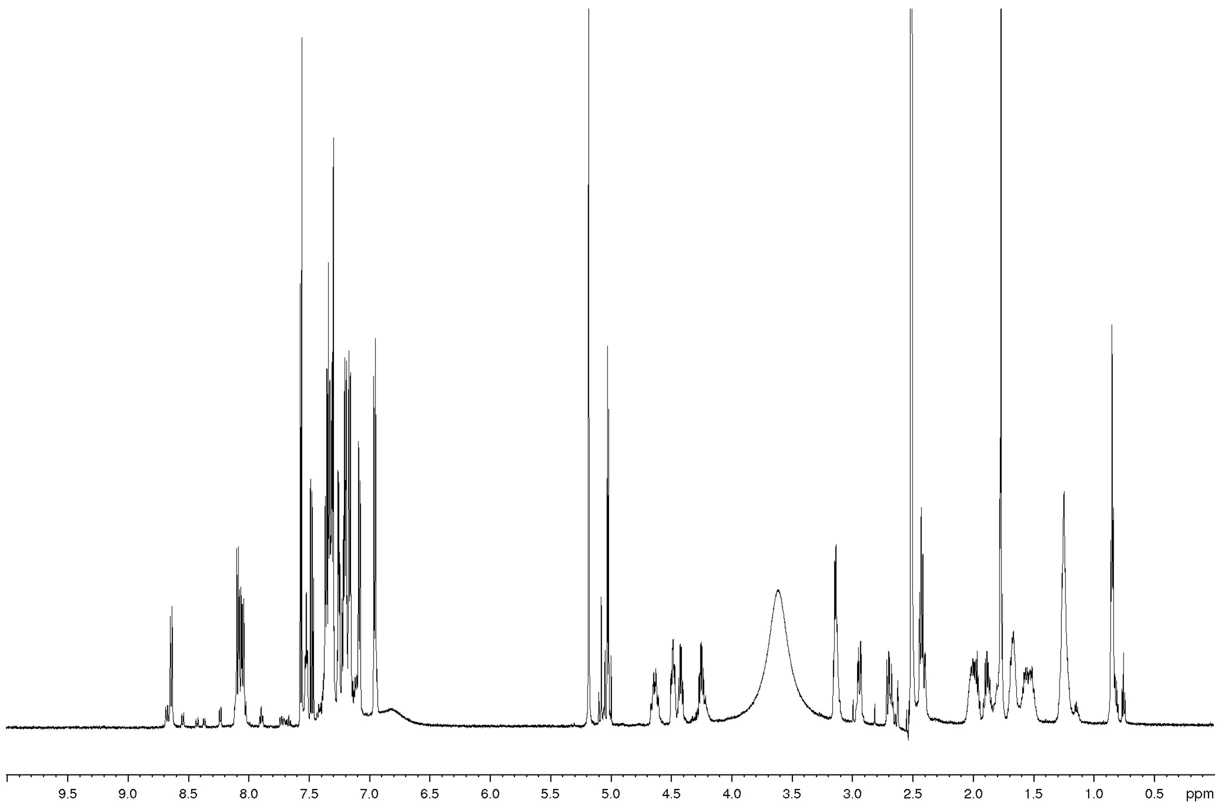

^{1}H NMR spectrum of I-SMXI5 (600 MHz, DMSO-d_6).

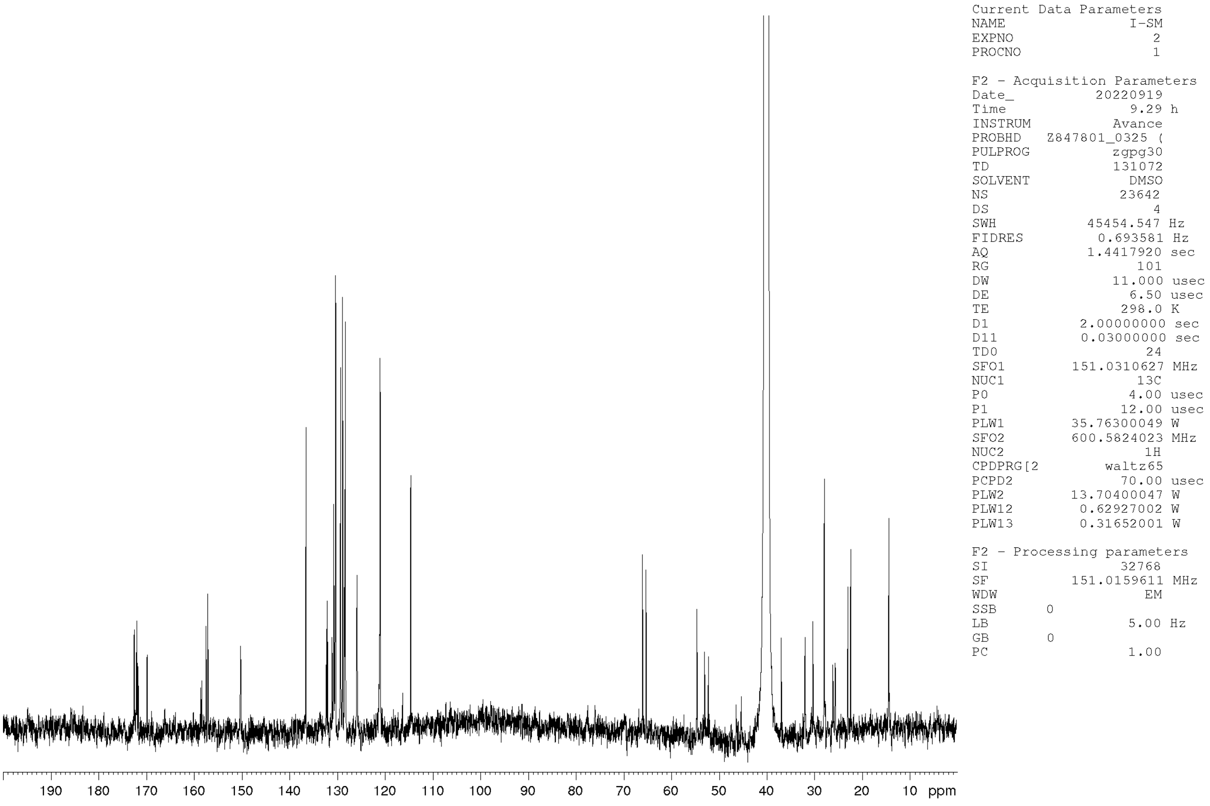

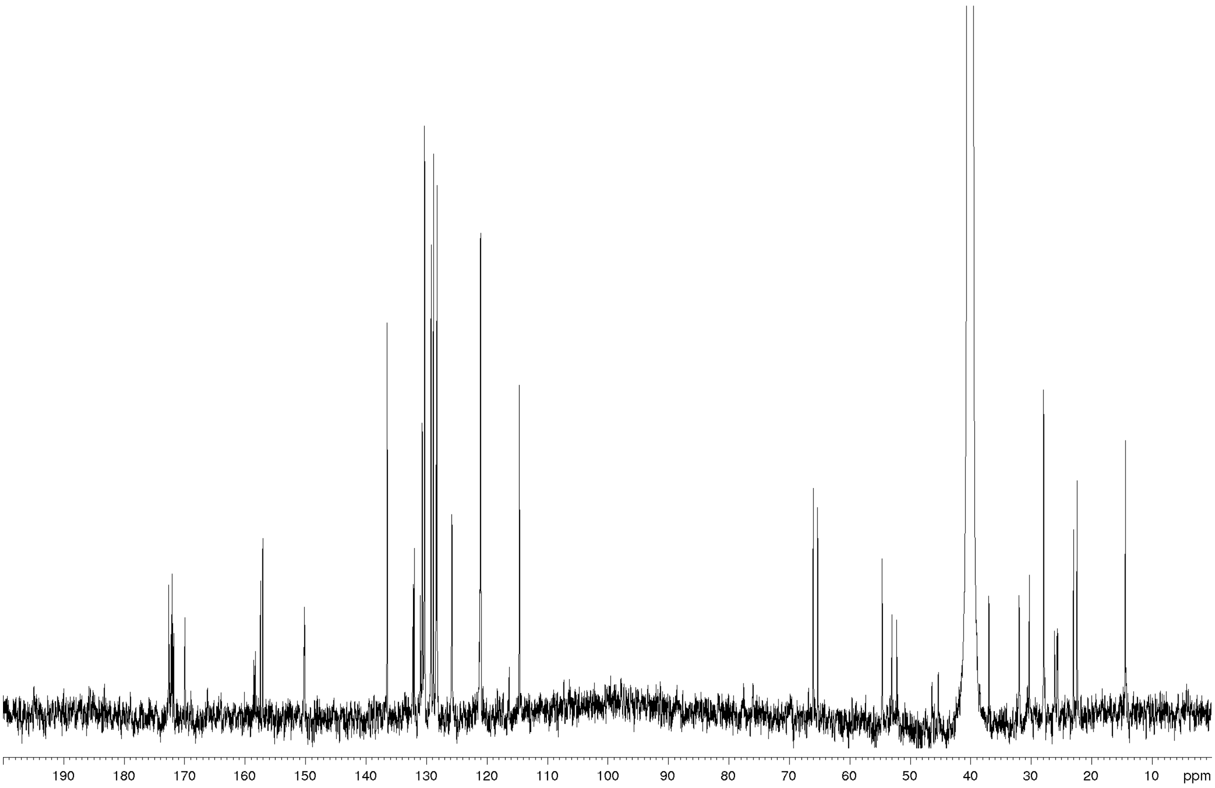

^{13}C NMR spectrum of I-SMXI5 (151 MHz, DMSO-d_6).

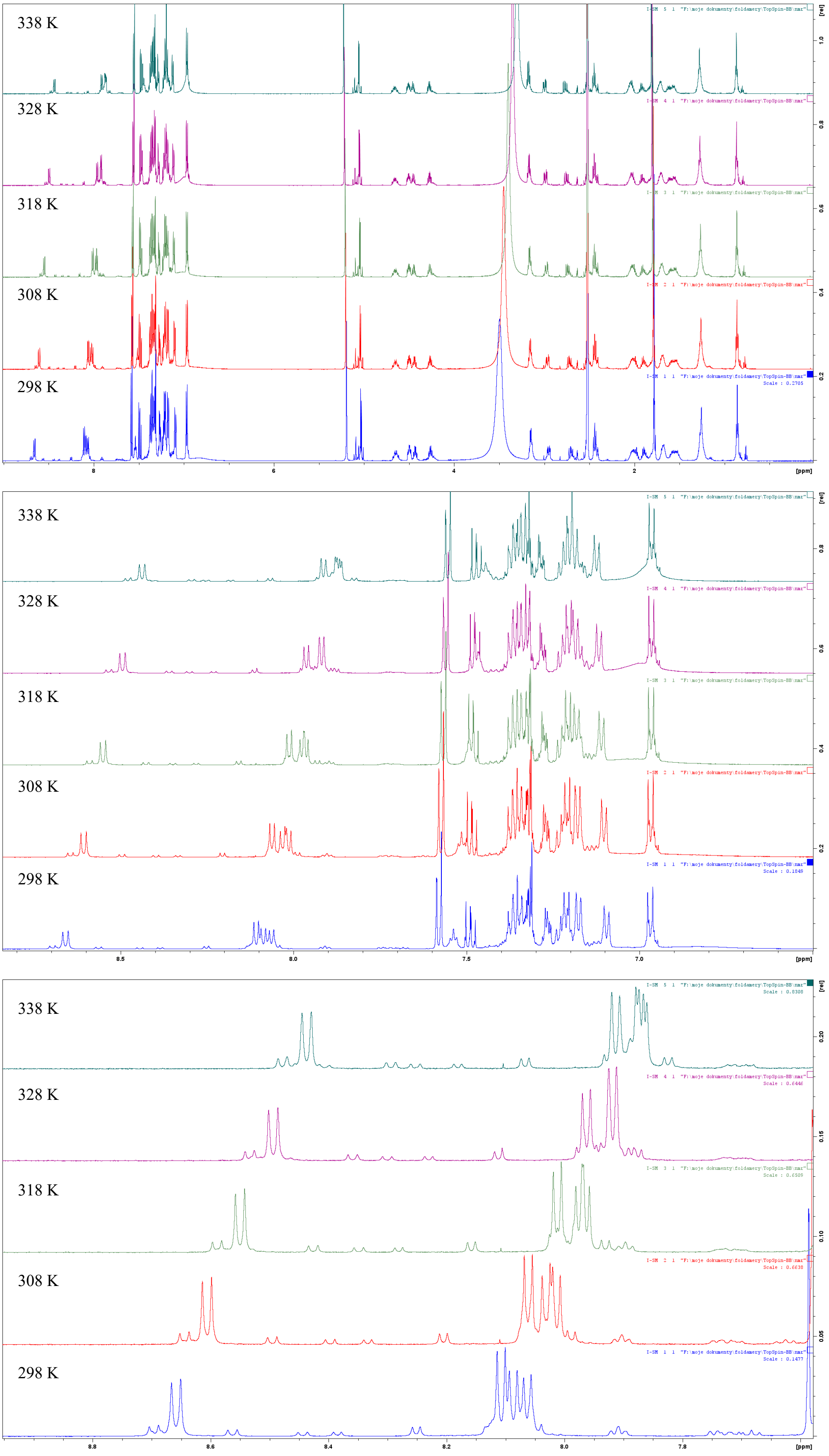

VT NMR spectrum of I-SMXI5 (600 MHz, DMSO-d_6).

To perform the NMR analysis, we used the Bruker Avance Neo spectrometer 600 MHz. P-SMXI51, P-SMXI52, and I-SMXI5 were dissolved in deuterated DMSO. ^{1}H NMR was performed at 600.58 MHz, and ^{13}C NMR was performed at 151.02 MHz. Since the P-SMXI52 sample was already dissolved in DMSO (not deuterated), we could only make ^{1}H NMR analysis by performing selective quenching of methyl groups from the solvent. The spectra provided are: ^{1}H NMR, ^{13}C NMR for P-SMXI51, I-SMXI5, and ^{1}H NMR for P-SMXI52. Variable Temperature NMR (VT NMR) experiments for P-SMXI51, P-SMXI52, and I-SMXI5 were performed at 600.58 MHz and the 298-338 K range every 10 K. Analyses were calibrated for the residual signals from DMSO.

Full-size blots

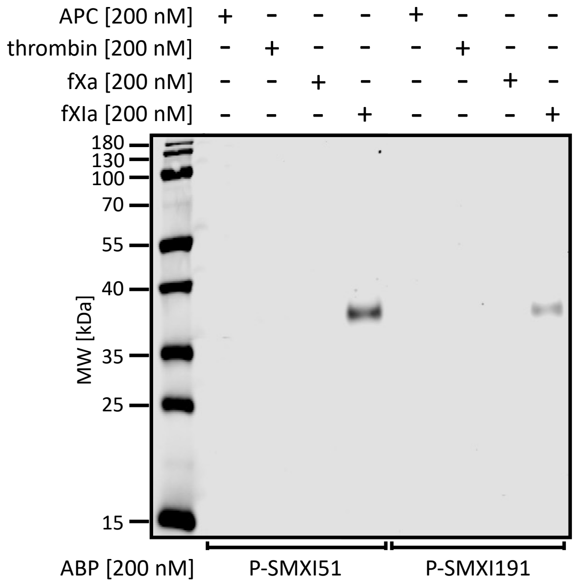

Figure S3. Labeling of purified coagulation factors (APC, thrombin, fXa, fXIa) using biotinylated ABPs.

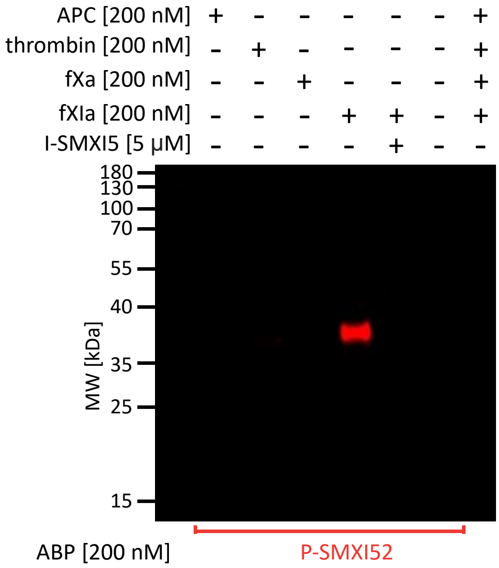

Figure S4. Labeling of purified coagulation factors (APC, thrombin, fXa, fXIa) using fluorescent ABP.

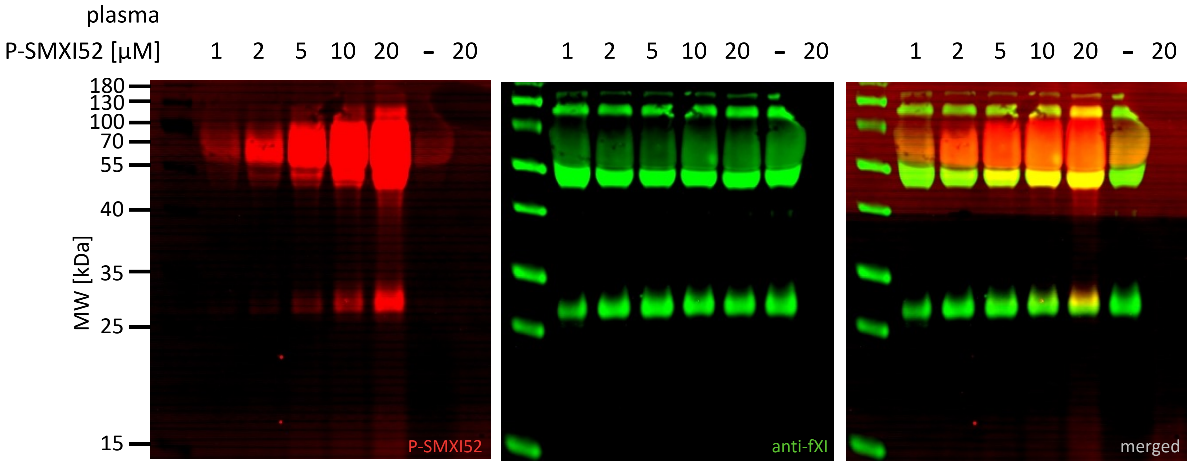

Figure S5. FXIa labeling in human plasma.

References

[1] S. Modrzycka, S. Kołt, S. Polderdijk, T. Adams, S. Potoczek, J. Huntington, P. Kasperkiewicz, M. Drąg; Parallel imaging of coagulation pathway proteases activated protein C, thrombin, and factor Xa in human plasma; *Chemical Science*, 2022, 13, 6813-6829.

[2] J. Oleksyszyn, J.C. Powers; Amino Acid and Peptide Phosphonate Derivatives as Specific Inhibitors of Serine Peptidases; *Methods Enzymol*, 1994, 244, 423-441.
